# Ecological review of Afrotropical *Ixodes* (*Afrixodes*) ticks: Distribution and host diversity, with a focus on micromammals

**DOI:** 10.1016/j.crpvbd.2026.100392

**Published:** 2026-05-27

**Authors:** Camille Lorang, Denis Augot

**Affiliations:** a ANSES, INRAE, Ecole Nationale Vétérinaire d’Alfort, UMR BIPAR, Laboratoire de Santé Animale, Maisons-Alfort, 94700, France; b Oniris, INRAE, BIOEPAR, Nantes, France

**Keywords:** Ticks, Africa, *Ixodes* (*Afrixodes*), Rodents, Shrews, Tick-borne pathogens, Wildlife

## Abstract

Ixodidae (Acari: Ixodoidea) is a hard tick family well represented across the world, particularly the genus *Ixodes* being the most species-rich with more than 285 species described to date. Within this genus, the subgenus *Afrixodes* makes up the largest number of species among the different subgenera recognized and is almost exclusively endemic to the Afrotropical biogeographical region. This systematic review analysed 77 articles, following PRISMA guidelines, and highlighted the distribution of host data for species of the genus *Afrixodes* in Africa, with a focus on micromammals. Our data present the 66 species of this subgenus, classified by developmental stage, highlighting the lack of descriptions of the adult male, nymph and larva stages, geographical distribution, host specificity (over 200 mammal species, 28 bird species and one reptile) and the pathogens they harbour. We emphasize on micromammals, rodents and shrews, because due to their proximity to livestock and humans and their ecological niche common to many wildlife species, they have a very significant potential to spread ticks and tick-borne pathogens and have been under-investigated relative to large domestic or wild ungulates until now. This comprehensive analysis provides insights into the potential role of micromammals (rodents and shrews) in the spread of ticks and tick-borne pathogens.

## Highlights

• A systematic review of Afrotropical *Ixodes* (*Afrixodes*) revealed data for 66 tick species.
• The distribution range of 64 species includes Eastern, Western and Southern tropical Africa and Madagascar.
• Two species are endemic to Sri Lanka and India.
• *Afrixodes* spp. were found to parasitize more than 200 mammal species and 28 bird species.
• Shrews and rodents represent 46% of host species recorded in the literature.

## Graphical abstract

**Figure:**
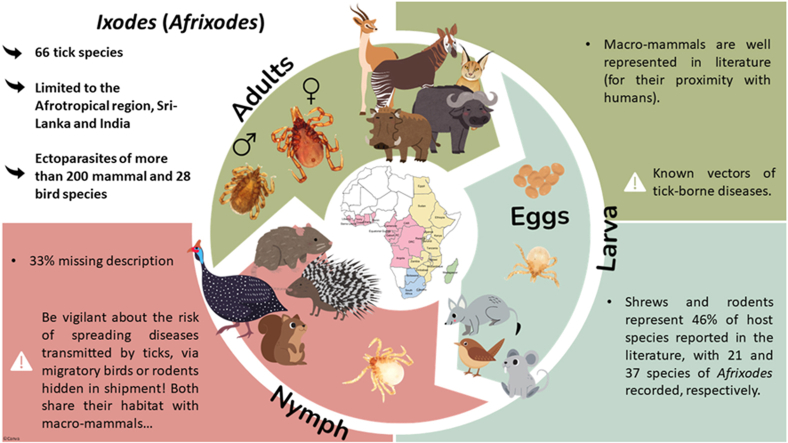

## 1 Introduction

Ticks are blood-feeding ectoparasites, known to be involved in a large variety of diseases in humans and animals. With 285 out of 772 hard tick species considered valid (Guglielmone et al., 2020; Mumcuoglu et al., 2025), *Ixodes* is the most diverse genus of the family Ixodidae. Within this genus, 14 subgenera (Clifford et al., 1973; Hornok et al., 2023) have been recognized based on morphological characters, with *Afrixodes* Morel, 1966 being the most species-rich with more than 60 species.

Catalogues are a vital resource not only for systematists revising groups and taxa, but also for ecologists, conservation biologists and many others who study tick diversity. There are many catalogues and studies (about 14) that focus on Ixodidae, including details on their distribution, ecology and host diversity, as well as checklists of species and their classification into subgenera with information on type-specimens, synonyms, nomenclatural status (e.g. *nomina dubia*), etc. (e.g. Camicas et al., 1998; Robbins et al., 2025). In contrast, there is relatively little information in the literature explicitly addressing species of *Afrixodes* as a whole. First, the number of species in the subgenus *Afrixodes* has increased since the 1970s; from the 42 described species in the late 1960s (Morel, 1969), this figure has increased to 44 (Clifford et al., 1973), then to 60 (Camicas et al., 1998; Guglielmone et al., 2020), and to date, 65 species have been classified as belonging to this subgenus (Robbins et al., 2025). Secondly, the distribution of *Afrixodes* spp. is limited to Africa and Madagascar (Morel, 1969), with only two species outside this area and being endemic to India and Sri Lanka (Kohls, 1948; Morel, 1966; Rajagopalan et al., 1968; Yamathramullage et al., 2018; Guglielmone et al., 2023). *Afrixodes* spp. were found in Western, Eastern and Southern Africa (Guglielmone et al., 2023), but only in tropical climates in the Afrotropical biogeographical region (sub-Saharan, equatorial and southern tropics) (Camicas et al., 1998; Guglielmone et al., 2023). They seem to be more frequently associated with mountain forests (Morel, 1969; Kolonin, 2007). Recently, Guglielmone et al. (2023) reviewed and listed the geographical distribution of these ticks.

The greatest body of work on the subgenus *Afrixodes* corresponds to the studies undertaken by Morel in the 1960s, which focused on all life cycle stages and both sexes of the adult stage, with notes on the distribution area and host diversity (Morel, 1965, 1966, 1969; Morel and Mouchet, 1965). *Afrixodes* spp. have a diverse panel of potential hosts, from macromammals to micromammals (Kolonin, 2007), but also include birds (Morel and Mouchet, 1965; Morel, 1966; Hoogstraal and Wassef, 1983; Guglielmone et al., 2020). Host distribution clearly influences tick species distribution. For example, a species of *Afrixodes*, *I*. *cumulatimpunctatus*, has already been reported in Europe, on a migratory bird in Malta (Hornok et al., 2022). In addition to birds, micromammals, such as rodents, can be passively dispersed *via* human activities such as international trade, notably *via* cargo shipments (Song et al., 2003; Heuser et al., 2025) and pose a biosecurity risk through the presence of their ectoparasites, such as ticks (particularly females or nymphs). The adult stages of *Afrixodes* spp. are usually found on large mammals such as carnivores (order Carnivora) and even-toed ungulates (order Artiodactyla), including domestic or wild bovids (Aeschlimann, 1967; Cumming, 1998; Kolonin, 2007). Little is known about the immature stages, nymphs and larvae, of *Afrixodes* spp., which are not often described. Immature stages are mostly present on small animals such as rodents (order Rodentia) and shrews (orders Afrosoricida, Soricomorpha, Macroscelidea) (Horak et al., 2005); micromammals are difficult to catch, making them harder to study.

Regarding tick-borne diseases, it is important to investigate micromammals, particularly to assess the connection between ticks, their hosts and pathogens (Ledwaba et al., 2022). For instance, large wild mammals hold great potential to spread tick-borne pathogens (TBPs) among livestock (Tonetti et al., 2009). However, rodents and shrews may also play a role in the parasite-pathogen cycles, because they share the ecological niches of all potential hosts of *Afrixodes* spp. (cattle and birds) and may thus act as a bridge between wild and domestic fauna. Known to be reservoirs of TBPs (Mlera and Bloom, 2018; Yamathramullage et al., 2018; Dumas et al., 2022; Rasoamalala et al., 2022; Veinović et al., 2024), micromammals can play a significant role in the eco-epidemiology of these diseases.

In a One Health context, it is important to identify the different tick species that pose a risk to human and animal health and to the environment, at all stages and all sexes. A better knowledge of their biology, including a list of host species, may also help identify at-risk hosts and potential pathogens, thereby facilitating the development of preventive measures.

This study, therefore, aims to provide a comprehensive overview of the species belonging to the subgenus *Afrixodes*, following the PRISMA (Preferred Reporting Items for Systematic Reviews and Meta-Analyses) guidelines (Page et al., 2021). The compilation of the data reviewed provides a complete list of *Afrixodes* spp., highlighting their host preferences and distribution, as well as any associated pathogens currently reported in the literature, with a focus on micromammals, due to their status as feeding hosts for ectoparasites and notably ticks.

## 2 Materials and methods

### 2.1 Literature search

**Fig. 1:**
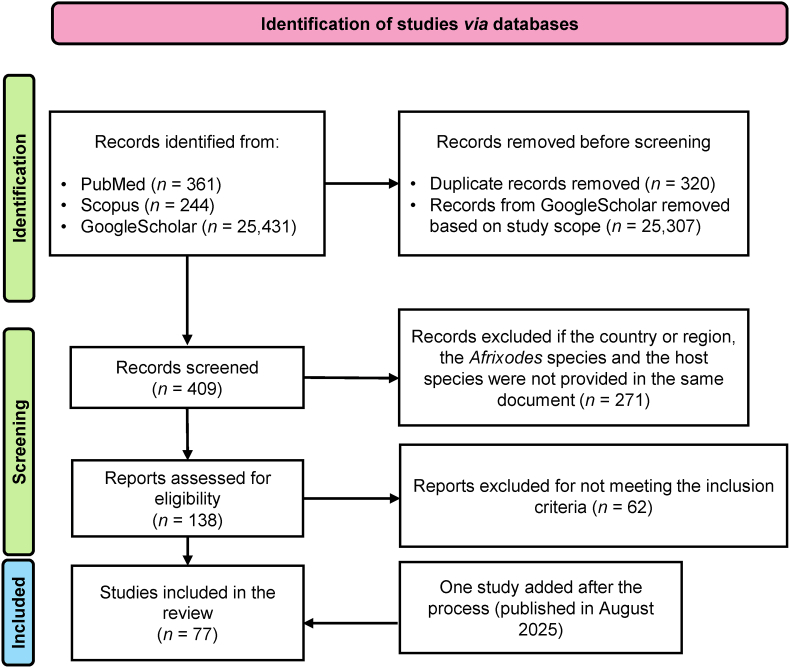
PRISMA workflow diagram.

The study was conducted according to the PRISMA guidelines (Page et al., 2021) to conduct a systematic literature review (Fig. 1, Supplementary Table S1). PubMed, Scopus, and Google Scholar databases were searched using search strings containing a combination of terms that included: “*Afrixodes*”; “*Afrixodes*” AND “host”; “Africa” AND “*Ixodes*”; “*Ixodes*” AND “African country name (ancient and actual)”; “*Ixodes*” AND “species name (valid and synonym)” (Fig. 1). The research covered publications from 1916 to July 2025 in French or English languages and all duplicates have been removed manually (Fig. 1). The title and abstract of each of the 409 articles selected for screening were read to identify whether they mentioned Africa, Sri Lanka or India, *Afrixodes* species and the host species (Fig. 1).

### 2.2 Inclusion and exclusion criteria

Full-text documents (9 monographs and 129 journal articles) were read. The collected information was screened and extracted by CL. The selection was made by verifying whether the articles and books include the following criteria: (i) Does the article mention a species of *Afrixodes*?; (ii) Does the article associate it with a host from which the specimen was collected (scientific or common name)?; and (iii) Does the geographical area correspond to Africa, Sri Lanka, or India?

Exclusion criteria were defined as: (i) publications not concerning species of *Afrixodes*; (ii) publications without indication of life cycle stage; (iii) research not based on ticks collected from animals; (iv) publications with no host associated with the tick record; and (v) publications in a language other than English or French. After this literature search, we added one article published on 27 August 2025, on a new *Ixodes* (*Afrixodes*) species, *Ixodes* (*A*.) *hyracis* (Apanaskevich et al., 2025a). Thus, the systematic review ultimately used data from 77 articles (Fig. 1).

A careful review of the selected articles (including the bibliographical references) enabled us to search for any missing documents. The original descriptions of all *Afrixodes* spp. were also considered.

### 2.3 Data synthesis

To update data from publications that used former country names, current country names were used. Similarly, the scientific names of hosts considered to be synonymous after the initial publication were updated to the currently valid names. All of this information was summarized to obtain a concise dataset. Two tables were prepared, collating the data from the reviewed publications for *Afrixodes* spp. as follows: host order; host family; host species (scientific name); host species (common name); associated *Ixodes* (*Afrixodes*) species; life cycle stage; locality, reference (Supplementary Tables S3 and S4). With these data, pie charts and bar charts were plotted in Excel to summarize data for mammal, bird, rodent and shrew hosts and *Afrixodes* spp. life stages. Records with undetermined (UD) host species were excluded from these graphs but are available in Supplementary Tables S3 and S4. Undetermined tick sex and/or life cycle stage were retained.

## 3 Results

### 3.1 Search outcomes

The full search retrieved 26,036 documents (25,431 articles identified in Google Scholar, 361 in PubMed, and 244 in Scopus) (Fig. 1). A total of 320 duplicate records were removed manually and a total of 25,307 records from Google Scholar were removed based on study scope. Of the 409 remaining articles, 271 were excluded due to of the lack of host-tick association identification (Fig. 1). A total of 138 articles were full-text read and 62 were excluded for not meeting the inclusion criteria (Fig. 1). A total of 77 publications (9 monographs and 68 journal articles) were used in this review (Fig. 1); of these, one was published before 1930, 10 were published during 1930–1959, 16 were published during 1960–1979, 11 were published during 1980–1999, and 39 were published during 2000–2025. The first publication used here is that of Nuttall (1916), and the most recent one is that of Apanaskevich et al. (2025a).

### 3.2 Species of *Afrixodes*

The three databases returned different but complementary results. Google Scholar was useful to find some books, paper manuscripts or museum series, unavailable on PubMed or Scopus (e.g. Nuttall, 1916; Hoogstraal, 1956; Arthur and Burrow, 1957). In PubMed and Scopus searches, studies on only 17 species of *Afrixodes* were found, compared with 35 species found in the Google Scholar search. All 66 species are listed in Supplementary Table S2 with data about the life stage or sex of the type-material deposited in museum collections. According to Apanaskevich et al. (2025a), the number of species may increase from 66 to 67, with a new species description coming soon. A list of the species reported in the reviewed papers and their regional distribution is provided in Table 1.

**Table 1:** Distribution of the species of *Ixodes* (*Afrixodes*) reported in the reviewed publications.

No.	Species of *Ixodes* (*Afrixodes*)	Region A (Eastern Africa)	Region B (Western Africa)	Region C (Southern Africa)	Region D (Madagascar)	Other	Reference
1	*I.* (*A.*) *albignaci* Uilenberg & Hoogstraal, 1970				Madagascar		Uilenberg et al. (1970, 1979); Kolonin (2007); Apanaskevich and Goodman (2020)
2	*I.* (*A.*) *ambohitantelensis* Englert, Goodman & Apanaskevich, 2023				Madagascar		Englert et al. (2023)
3	*I.* (*A.*) *ampullaceus* Warburton, 1933	Malawi, Mozambique, Tanzania, Uganda, Zimbabwe		South Africa			Warburton (1933); Apanaskevich et al. (2025b)
4	*I.* (*A.*) *arebiensis* Arthur, 1956					UD (Africa)	Arthur (1956); Cumming (1998)
5	*I.* (*A.*) *aulacodi* Arthur, 1956		Benin, Cameroon, CAR, Côte d’Ivoire, DRC, Gabon, Ghana, RC, Zambia	South Africa			Arthur (1956); Clifford and Anastos (1962); Morel and Mouchet (1965); Elbl and Anastos (1966); Morel (1966); Aeschliman (1967); Walker (1991); Cumming (1998); Ntiamoa-Baidu et al. (2004, 2005); Uilenberg et al. (2013); Chitimia-Dobler et al. (2016); Yessinou et al. (2022); Apanaskevich et al. (2025b)
6	*I.* (*A.*) *auriculaelongae* Arthur, 1958	Tanzania, Zambia	DRC				Arthur (1958); Elbl and Anastos (1966); Colbo and MacLeod (1976); Cumming (1998)
7	*I.* (*A.*) *bakeri* Arthur & Clifford, 1961			South Africa			Atthur and Clifford (1961); Walker (1991); Petney et al. (2004); Matthee et al. (2007, 2010)
8	*I.* (*A.*) *bedfordi* Arthur, 1959			Lesotho			Arthur (1959); Walker (1991); Cumming (1998)
9	*I.* (*A.*) *brewsterae* Keirans, Clifford & Walker, 1982	Uganda	DRC, Liberia				Keirans et al. (1982); Kolonin (2007)
10	*I.* (*A.*) *browningi* Arthur, 1956	Rwanda	DRC				Arthur (1956); Elbl and Anastos (1966); Cumming (1998)
11	*I.* (*A.*) *brumpti* Morel, 1965	Ethiopia					Morel (1965); Kolonin (2007)
12	*I.* (*A.*) *calcarhebes* Arthur & Zulu, 1980	Zambia					Arthur and Zulu (1980)
13	*I.* (*A.*) *catherinei* Keirans, Clifford & Walker, 1982			South Africa			Keirans et al. (1982); Walker (1991)
14	*I.* (*A.*) *cavipalpus* Nuttall & Warburton, 1908	Sudan, Tanzania	DRC	Botswana, South Africa			Nuttall et al. (1908); Hoogstraal (1956); Hoogstraal and Theiler (1959); Clifford and Anastos (1962); Elbl and Anastos (1966); Clifford et al. (1975); Cumming (1998)
15	*I.* (*A.*) *ceylonensis* Kohls, 1950					India, Sri Lanka (Asia)	Kohls (1950); Rajagopalan et al. (1968); Bhat and Sreenivasan (1981); Kolonin (2007); Yamathramullage et al. (2014); Muraleedharan (2017); Shobana and Gunasekaran (2018); Yamathramullage et al. (2018); Guglielmone et al. (2020)
16	*I.* (*A.*) *colasbelcouri* Arthur, 1957				Madagascar		Arthur (1957c); Uilenberg et al. (1979); Apanaskevich and Goodman (2020)
17	*I.* (*A.*) *corwini* Keirans, Clifford & Walker, 1982			South Africa			Keirans et al. (1982); Horak et al. (1987); Walker (1991)
18	*I.* (*A.*) *cumulatimpinctatus* Schulze, 1943	Kenya, Malawi, Rwanda, Uganda, Zambia, Zimbabwe	Cameroon, DRC, CAR, Equatorial Guinea, Gabon, Ghana, RC			Malta (Europe)	Schulze (1943); Arthur and Burrow (1957); Morel and Mouchet (1965); Elbl and Anastos (1966); Morel (1966); Aeschliman (1967); Colbo and MacLeod (1976); Cumming (1998); Ntiamoa-Baidu et al. (2004, 2005); Pourrut et al. (2011); Uilenberg et al. (2013); Ngoy et al. (2021); Hornok et al. (2022)
19	*I.* (*A.*) *dawesi* Arthur, 1956		DRC				Arthur (1956); Elbl and Anastos (1966); Kolonin (2007)
20	*I.* (*A.*) *djaronensis* Neumann, 1907	Tanzania					Neumann (1907); Morel (1965); Kolonin (2007); Guglielmone et al. (2020)
21	*I.* (*A.*) *drakensbergensis* Clifford, Theiler & Baker, 1975			South Africa			Clifford et al. (1975); Kolonin (2007)
22	*I.* (*A.*) *elongatus* Bedford, 1929			South Africa			Bedford (1929); Arthur (1959); Walker (1991); Cumming (1998); Matthee et al. (2010)
23	*I.* (*A.*) *euplecti* Arthur, 1958	Egypt, Ethiopia, Zambia	RC				Arthur (1958); Colbo and MacLeod (1976); Hoogstraal and Wassef (1983)
24	*I.* (*A.*) *evansi* Arthur, 1956		DRC				Arthur (1956); Cumming (1998)
25	*I.* (*A.*) *fynbosensis* Apanaskevich, Horak, Matthee & Matthee, 2011			South Africa			Matthee et al. (2010); Apanaskevich et al. (2011)
26	*I.* (*A.*) *heinrichi* Arthur, 1962		Angola				Arthur (1962); Kolonin (2007); Guglielmone et al. (2020)
27	*I.* (*A.*) *hyracis* Apanaskevich, Drew & Pienaar, 2025			South Africa			Apanaskevich et al. (2025a)
28	*I.* (*A.*) *latus* Arthur, 1958	Malawi					Arthur (1958); Cumming (1998); Guglielmone et al. (2020)
29	*I.* (*A.*) *lemuris* Arthur, 1957				Madagascar		Arthur (1956, 1957c); Uilenberg et al. (1979); Kolonin (2007); Blanco et al. (2013)
30	*I.* (*A.*) *lewisi* Arthur, 1965	Zambia					Arthur (1965); Cumming (1998); Guglielmone et al. (2020)
31	*I.* (*A.*) *loveridgei* Arthur, 1958	Malawi	Ghana				Arthur (1958); Cumming (1998); Ntiamoa-Baidu et al. (2004); Kolonin (2007)
32	*I.* (*A.*) *lunatus* Neumann, 1907				Madagascar		Neumann (1907); Arthur (1957a); Uilenberg et al. (1979); Apanaskevich and Goodman (2020)
33	*I.* (*A.*) *macfarlanei* Keirans, Clifford & Walker, 1982	Uganda	DRC				Keirans et al. (1982); Guglielmone et al. (2020)
34	*I.* (*A.*) *matopi* Spickett, Keirans, Norval & Clifford, 1981	Zimbabwe					Spickett et al. (1981); Colborne et al. (1981); Cumming (1998); Kolonin (2007)
35	*I.* (*A.*) *microgalei* Apanaskevich, Soarimalala & Goodman, 2013				Madagascar		Apanaskevich et al. (2013); Apanaskevich and Goodman (2020)
36	*I.* (*A.*) *minutae* Arthur, 1959					UD (Africa)	Arthur (1959); Kolonin (2007)
37	*I.* (*A.*) *moreli* Arthur, 1957		Ghana, Côte d’Ivoire				Arthur (1957b); Elbl and Anastos (1966); Aeschliman (1967); Cumming (1998, 1999); Ntiamoa-Baidu et al. (2004, 2005)
38	*I.* (*A.*) *muniensis* Arthur & Burrow, 1957	Kenya, Rwanda	Cameroon, DRC, Equatorial Guinea, Gabon, Ghana				Arthur and Burrow (1957); Morel and Mouchet (1965); Elbl and Anastos (1966); Aeschliman (1967); Cumming (1998); Ntiamoa-Baidu et al. (2004, 2005); Oguge et al. (2009); Lacroux et al. (2023)
39	*I.* (*A.*) *myotomys* Clifford & Hoogstraal, 1970			South Africa			Clifford and Hoogstraal (1970); Walker (1991); Cumming (1998); Kolonin (2007); Guglielmone et al. (2020)
40	*I.* (*A.*) *nairobiensis* Nuttall, 1916	Kenya, Uganda, Sudan	DRC	South Africa			Nuttall (1916); Hoogstraal (1956); Arthur (1959); Clifford and Anastos (1962); Elbl and Anastos (1966); Cumming (1998); Kolonin (2007)
41	*I.* (*A.*) *nchisiensis* Arthur, 1958	Malawi	CAR, DRC, Côte d’Ivoire				Arthur (1958); Theiler (1962); Elbl and Anastos (1966); Morel (1966); Keirans et al. (1982); Cumming (1998); Kolonin (2007)
42	*I.* (*A.*) *neitzi* Clifford, Walker & Keirans, 1977	Zimbabwe		South Africa			Clifford et al. (1977); Rechav et al. (1978); Walker (1991); Cumming (1998); Kolonin (2007); Baauw et al. (2019); Ledwaba et al. (2022)
43	*I.* (*A.*) *nesomys* Uilenberg & Hoogstraal, 1969				Madagascar		Uilenberg and Hoogstraal (1969a); Uilenberg et al. (1979); Kolonin (2007); Apanaskevich and Goodman (2020)
44	*I.* (*A.*) *nicolasi* Santos Dias, 1982	Mozambique					Santos Dias (1982); Guglielmone et al. (2023)
45	*I.* (*A.*) *okapiae* Arthur, 1956		DRC				Arthur (1956); Elbl and Anastos (1966); Kolonin (2007)
46	*I.* (*A.*) *oldi* Nuttall, 1913	Kenya, Tanzania, Zambia	DRC, Ghana, Côte d’Ivoire, Liberia				Nuttall (1913); Arthur (1958); Elbl and Anastos (1966); Aeschliman (1967); Keirans et al. (1982); Cumming (1998, 1999); Ntiamoa-Baidu et al. (2004, 2005); Kolonin (2007)
47	*I.* (*A.*) *pilosus* Koch, 1844			South Africa			Koch (1844); Bedford (1936); Clifford et al. (1975); Horak et al. (1987); Walker (1991); Cumming (1998, 1999)
48	*I.* (*A.*) *procaviae* Arthur & Burrow, 1957	Burundi, Rwanda	DRC	South Africa			Arthur and Burrow (1957); Morel (1965); Elbl and Anastos (1966); Horak et al. (1987); Walker (1991); Cumming (1998); Horak et al. (2000); Golezardy and Horak (2007); Horak et al. (2007); Kolonin (2007); Hasle et al. (2009); Matthee et al. (2010); Viljoen et al. (2020); Ledwaba et al. (2022)
49	*I.* (*A.*) *radfordi* Kohls, 1948					India (Asia)	Kohls (1948); Bhat and Sreenivasan (1981); Muraleedharan (2017); Guglielmone et al. (2020)
50	*I.* (*A.*) *rageaui* Arthur, 1957	Rwanda	Cameroon, CAR, Gabon				Arthur (1957c); Hoogstraal and Theiler (1959); Morel and Mouchet (1965); Elbl and Anastos (1966); Cumming (1998, 1999); Kolonin (2007); Pourrut et al. (2011); Uilenberg et al. (2013)
51	*I.* (*A.*) *randrianasoloi* Uilenberg & Hoogstraal, 1969				Madagascar		Uilenberg and Hoogstraal (1969b); Uilenberg et al. (1979); Kolonin (2007); Apanaskevich and Goodman (2020); Guglielmone et al. (2020)
52	*I.* (*A.*) *rasus* Neumann, 1899	Malawi, Rwanda, Uganda, Zambia	Cameroon, CAR, DRC, Equatorial Guinea, Gabon, Ghana				Neumann (1899); Arthur and Burrow (1957); Hoogstraal and Theiler (1959); Morel and Mouchet (1965); Elbl and Anastos (1966); Aeschliman (1967); Colbo and MacLeod (1976); Cumming (1998); Ntiamoa-Baidu et al. (2004, 2005); Pourrut et al. (2011); Uilenberg et al. (2013)
53	*I.* (*A.*) *rhabdomysae* Arthur, 1959	Zambia		South Africa			Arthur (1959); Colbo and MacLeod (1976); Walker (1991); Cumming (1998)
54	*I.* (*A.*) *rotundatus* Arthur, 1958	Kenya, Uganda	DRC				Arthur (1958); Elbl and Anastos (1966); Cumming (1998, 1999)
55	*I.* (*A.*) *rubicundus* Neumann, 1904			South Africa			Neumann (1904); Clifford et al. (1975); Horak et al. (1987); Walker (1991); Cumming (1998, 1999); Horak et al. (2000, 2005, 2007); Kolonin (2007); Matthee et al. (2007); Tonetti et al. (2009); Berggoetz et al. (2014); Horak et al. (2015); Viljoen et al. (2020); Ledwaba et al. (2022)
56	*I.* (*A.*) *schillingsi* Neumann, 1901	Burundi, Kenya, Sudan, Tanzania, Zanzibar					Neumann (1901); Arthur (1957c); Hoogstraal (1956); Hoogstraal and Theiler (1959); Kolonin (2007); Guglielmone et al. (2020)
57	*I.* (*A.*) *soarimalalae* Apanaskevich & Goodman, 2020				Madagascar		Apanaskevich and Goodman (2020)
58	*I.* (*A.*) *spinae* Arthur, 1958		DRC	South Africa			Arthur (1958); Clifford and Anastos (1962); Cumming (1998); Kolonin (2007); Hasle et al. (2009)
59	*I.* (*A.*) *thomasae* Arthur & Burrow, 1957	Kenya, Tanzania	DRC				Arthur and Burrow (1957); Elbl and Anastos (1966); Colbo and MacLeod (1976); Cumming (1998); Kolonin (2007); Guglielmone et al. (2020)
60	*I.* (*A.*) *transvaalensis* Clifford & Hoogstraal, 1966			South Africa			Clifford and Hoogstraal (1966); Walker (1991); Kolonin (2007)
61	*I.* (*A.*) *ugandanus* Neumann, 1906	Ethiopia, Uganda		South Africa			Neumann (1906); Clifford et al. (1977); Walker (1991); Cumming (1998); Kolonin (2007); Guglielmone et al. (2020); Apanaskevich et al. (2025b)
62	*I.* (*A.*) *uilenbergi* Apanaskevich & Goodman, 2020				Madagascar		Apanaskevich and Goodman (2020)
63	*I.* (*A.*) *uncus* Apanaskevich & Goodman, 2020				Madagascar		Apanaskevich and Goodman (2020)
64	*I.* (*A.*) *vanidicus* Schulze, 1943	Mozambique	Cameroon				Schulze (1943); Morel and Mouchet (1965); Keirans et al. (1982); Cumming (1998); Guglielmone et al. (2020)
65	*I.* (*A.*) *walkerae* Clifford, Kohls & Hoogstraal, 1968	Kenya					Clifford et al. (1968); Guglielmone et al. (2020)
66	*I.* (*A.*) *zairensis* Keirans, Clifford & Walker, 1982		DRC				Keirans et al. (1982); Kolonin (2007)

*Note*: References comprising the original descriptions of 28 species were added after completing the species list and citation search of the publications considered in the review.

*Abbreviations*: CAR, Central African Republic; DRC, Democratic Republic of the Congo; RC, Republic of Congo; UD, undetermined.

### 3.3 Geographical distribution

**Fig. 2:**
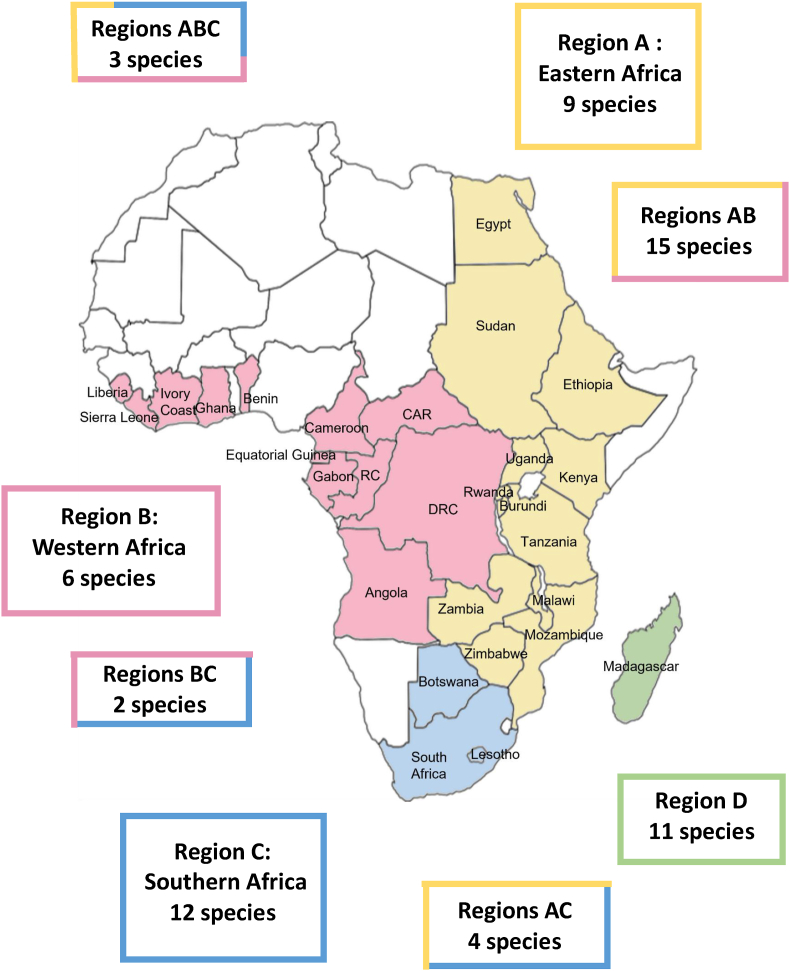
Map illustrating the regions and countries where species of the subgenus *Afrixodes* have been recorded on a host animal. Numbers represent the number of species reported only in a single region and the number of species shared between the regions. Details about the species distribution are given in Table 1. Two species endemic to India and Sri Lanka (*I. ceylonensis* and *I. radfordi*) and two species (*I. arebiensis* and *I. minutae*) for which no specific African locality was indicated are not considered in Fig. 2. Uncolored areas indicate countries with no data retrieved. *Abbreviations*: CAR, Central African Republic; DRC, Democratic Republic of the Congo; RC, Republic of Congo.

Based on this review, the distribution of *Afrixodes* species, linked to wildlife, spans from Western to Eastern Africa, and includes Southern Africa (Table 1, Fig. 2). Totals of 31, 26 and 21 species were recorded in Eastern, Western and Southern Africa, respectively, and 11 species were endemic to Madagascar (Regions A, B, C, and D in Fig. 2, respectively; see Table 1).

A total of 27 species were reported in a single region: Eastern (*n* = 9 spp.; *I. brumpti*, *I. calcarhebes*, *I. djaronensis*, *I. latus*, *I. lewisi*, *I. matopi*, *I. nicolasi*, *I. schillingsi*, and *I. walkerae*); Western (*n* = 6 spp.; *I. dawesi*, *I. evansi*, *I. heinrichi*, *I. moreli*, *I. okapiae*, and *I. zairensis*); and Southern Africa (*n* = 12 spp.; *I. bakeri*, *I. bedfordi*, *I. catherinei*, *I. corwini*, *I. drakensbergensis*, *I. elongatus*, *I. fynbosensis*, *I. hyracis*, *I. myotomys*, *I. pilosus*, *I. rubicundus* and *I. transvaalensis*) (Fig. 2, Table 1).

Twenty-one species had a wider distribution: 15 species were recorded in both Eastern and Western Africa (*I. auriculaelongae*, *I. brewsterae*, *I. browningi*, *I. cumulatimpunctatus*, *I. euplecti*, *I. loveridgei*, *I. macfarlanei*, *I. muniensis*, *I. nchisiensis*, *I. oldi*, *I. rageaui*, *I. rasus*, *I. rotundatus*, *I. thomasae*, and *I. vanidicus*); four species were recorded in both Eastern and Southern Africa (*I. ampullaceus*, *I. neitzi*, *I. rhabdomysae*, and *I. ugandanus*), and two species were recorded in both Western and Southern Africa (*I. aulacodi* and *I. spinae*) (Fig. 2, Table 1). Only three species (*I. cavipalpus, I. nairobiensis* and *I. procaviae*) were reported in all three regions (Fig. 2, Table 1).

Records for some species are restricted to a specific country. For example, 10 species were only found in South Africa, four in the Democratic Republic of the Congo (DRC) and two in Zambia (Table 1). There are also six species reported only in a single country: *I. bedfordi* in Lesotho; *I. brumpti* in Ethiopia; *I. djaronensis* in Tanzania; *I. latus* in Malawi; *I. matopi* in Zimbabwe; and *I. nicolasei* in Mozambique (Table 1, Supplementary Table S3). Interestingly, five species have been reported only once: *I. cavipalpus* in Botswana; *I. heinrichi* in Angola; *I. nchisiensis* in the Central African Republic; *I. oldi* in Sierra Leone; and *I. schillingsi* on Zanzibar Island (Supplementary Table S3). One observation of *I. euplecti* was reported in Egypt on a bird. Finally, although *I. arebiensis* and *I. minutae* are noted in Africa, no specific area was mentioned (Supplementary Table S3); these two species were therefore not included in Fig. 2.

### 3.4 Hosts of *Afrixodes* spp.: Mammals and birds

#### 3.4.1 Mammals

**Fig. 3:**
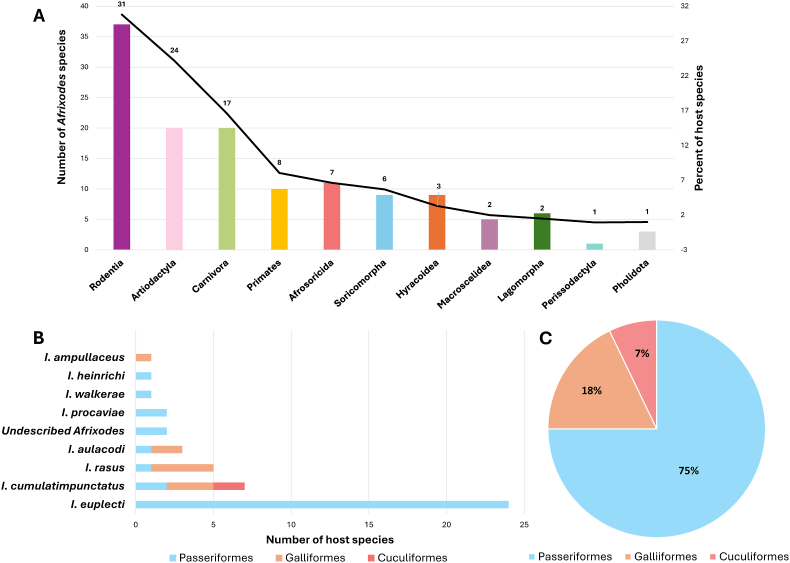
Diversity of vertebrates, by taxonomic order, parasitized by at least one tick species of the subgenus *Afrixodes* based on the studies included in the review. All details and references are available in Supplementary Table S3. **A** Bar chart for mammals, with the number of *Afrixodes* spp. found in a given animal order (left y-axis) and the percentage of animal species by host order (right y-axis). **B** Bar chart for birds, with the number of specimens per species found in a given bird order. **C** Pie chart representing the percentage of bird species (by taxonomic order) parasitized by a species of the genus *Afrixodes*.

In the publications selected for the review, 65 species (except *I. euplecti*) were reported for hosts of 11 mammal orders, representing 207 species (see Supplementary Table S4 for details). Rodentia was the most represented host group taxon (31% of all host species), followed by Artiodactyla (24%) and Carnivora (17%) (Fig. 3A). Rodentia was also the order with the largest number of *Afrixodes* species reported (37 species), followed by Artiodactyla and Carnivora (20 species each) (Fig. 3A).

Eight *Afrixodes* species appeared restricted to a single host species (Supplementary Table S4): *I. corwini* on *Aonyx capensis* (family Mustelidae); *I. djaronensis* on *Genetta tigrine* (Viverridae); *I. okapiae* on *Okapia johnstoni* (Giraffidae); and *I. evansi* on *Tragelaphus scriptus* (Bovidae). *Ixodes brumpti* was reported on *Heterohyrax brucei* (Hyracoidea: Procaviidae) and *I. spinae* and *I. hyracis* on *Procavia capensis* (Hyracoidea: Procaviidae). Similarly, *I. catherinei* was found on *Lepus saxatilis* (Lagomorpha: Leporidae) only once (Keirans et al., 1982).

Several tick species appear to be more generalist and were reported in a wider range of hosts (Supplementary Table S4): *I. drakensbergensis* on hosts of the Bovidae; *I. lewisi* on hosts of the Hyracoidea; *I. moreli* and *I. browningi* on hosts of the Rodentia; *I. pilosus* on hosts of the Lagomorpha; *I. brewsterae* and *I. corwini* on hosts of the Carnivora; *I. vanidicus* on hosts of the Carnivora and humans; *I. matopi* on hosts of the Bovidae, Procaviidae and Leporidae. Finally, nine species (*I. aulacodi*, *I. cavipalpus*, *I. cumulatimpunctatus*, *I. muniensis*, *I. nairobiensis*, *I. oldi*, *I. procaviae*, *I. rasus*, and *I. rubicundus*) were reported on many species of mammals, and three species (*I. ceylonensis*, *I. ampullaceus*, and *I. fynbosensis*) were found on both hosts of the Carnivora and micromammals. Finally, *I. rubicundus* is the only species found in hosts of the Equidae and Rhinocerotidae (Perissodactyla), as reported in two publications (Horak et al., 2007; Tonetti et al., 2009).

Interestingly, except for Malagasy primates, *I. rageaui* is the only parasite exclusive to primates on the African continent, along with *I. schillingsi*, for which there is nevertheless one report on *Lophiomys imhausi* (Rodentia: Cricetidae) (Supplementary Tables S3 and S4). Focusing on the family Galagidae, *I. schillingsi* and *I. rhabdomysae* are the only species to be reported on *Otolemur crassicaudatus* (Supplementary Tables S3 and S4).

In Madagascar, *I. lemuris* is the only species known to parasitize primates (*Cheirogaleus major*, *Microcebus* sp., and *Eulemur rufifrons*). Four species (*I*. *microgalei*, *I. soarimalale*, *I. uilenbergi*, and *I. uncus*) were found on endemic species of the Tenrecidae (Afrosoricida). *Ixodes nesomys* was found exclusively on species of Rodentia (Muridae and Nesomyidae). Regarding Muridae, the black rat (*Rattus rattus*) was reported as host for four tick species, *I. albignaci*, *I. colasbelcouri*, *I. lemuris*, and *I. randrianasoloi* (Table 1, Supplementary Tables S3 and S4).

#### 3.4.2 Birds

Species of three bird orders (Cuculiformes, Galliformes and Passeriformes) have been reported as hosts of *Afrixodes* tick species in the literature. Order Passeriformes (Fig. 3B) is the most represented in terms of the number of bird species hosting *Afrixodes* species (21 species of a total of 28 found, i.e. 75%) (Fig. 3C). Eight species have been reported on birds in the literature (Fig. 3B), which is equivalent to 12% of the species of the subgenus *Afrixodes*. Described as *I. spinae* by Hasle et al. (2009), the specimen found on *Euplectes orix* was subsequently identified as an undescribed species of *Afrixodes* by Apanaskevich et al. (2025a). *Ixodes euplecti* was exclusively found on birds (Supplementary Tables S3 and S4) and is specific to Passeriformes (Fig. 3B). Only four species were hosted on galliform birds, *I. ampullaceus*, *I. aulacodi*, *I. rasus*, and *I. cumulatimpunctatus*; the latter species appears to be the only one also to parasitize cuckoos (Fig. 3B). Finally, 11 species (*I. ampullaceus*, *I. arebiensis*, *I. aulacodi*, *I. ceylonensis*, *I. cumulatimpunctatus*, *I. euplecti*, *I. heinrichi*, *I. procaviae*, *I. rasus*, *I. thomasae*, and *I. walkerae)* appear to be highly generalist and share a host range that spans both micromammals and birds (Supplementary Table S4).

### 3.5 Hosts of *Afrixodes* spp.: Focus on rodents and shrews

Rodents (Rodentia) and shrews (Afrosoricida, Soricomorpha, and Macroscelidea) represent 46% of the host species mentioned in the literature (Fig. 3A). A total of 37 tick species were reported to parasitize rodents of eight families, while 21 tick species were reported to parasitize shrews of three families (Fig. 3A), with 13 tick species common to both groups of host taxa (Supplementary Table S4).

#### 3.5.1 Rodents

Our data show: (i) 27 tick species found on hosts of the Muridae (*I*. *albignaci*, *I. aulacodi*, *I. auriculaelongae*, *I. bakeri*, *I. bedfordi*, *I. browningi*, *I. calcarhebes*, *I. ceylonensis*, *I. colasbelcouri*, *I. cumulatimpunctatus*, *I. elongatus*, *I. fynbosensis*, *I. heinrichi*, *I. lemuris*, *I. muniensis*, *I. myotomys*, *I. nairobiensis*, *I. nchisiensis*, *I. oldi*, *I. procaviae*, *I. radfordi*, *I. randrianosoloi*, *I. rasus*, *I. rhabdomysae*, *I. rubicundus*, *I. thomasae*, and *I. transvaalensis*); (ii) ten species reported on hosts of the Nesomyidae (*I*. *aulacodi*, *I. cumulatimpunctatus*, *I. colasbelcouri*, *I. loveridgei*, *I. muniensis*, *I. oldi*, *I. rasus*, *I. nesomys*, *I. rhabdomysae*, and *I. rubicundus*); (iii) six species reported on hosts of the Thryonomyidae (*I*. *browningi*, *I. ceylonensis*, *I. cumulatimpunctatus*, *I. latus*, *I. muniensis*, and *I. oldi*); (iv) six species reported on hosts of the Sciuridae (*I*. *aulacodi*, *I. cumulatimpunctatus*, *I. moreli*, *I. muniensis*, *I. rasus*, and *I. ugandanus*); (v) four species reported on hosts of the Hystricidae (*I*. *aulacodi*, *I. ceylonensis*, *I. cumulatimpunctatus*, and *I. rasus*); (vi) two species reported on hosts of the Spalacidae (*I*. *oldi* and *I. rotundatus*); (vii) one species reported on hosts of the Cricetidae (*I*. *schillingsi*); and (viii) one species reported on hosts of the Gliridae (*I*. *muniensis*) (Supplementary Tables S3 and S4).

**Fig. 4:**
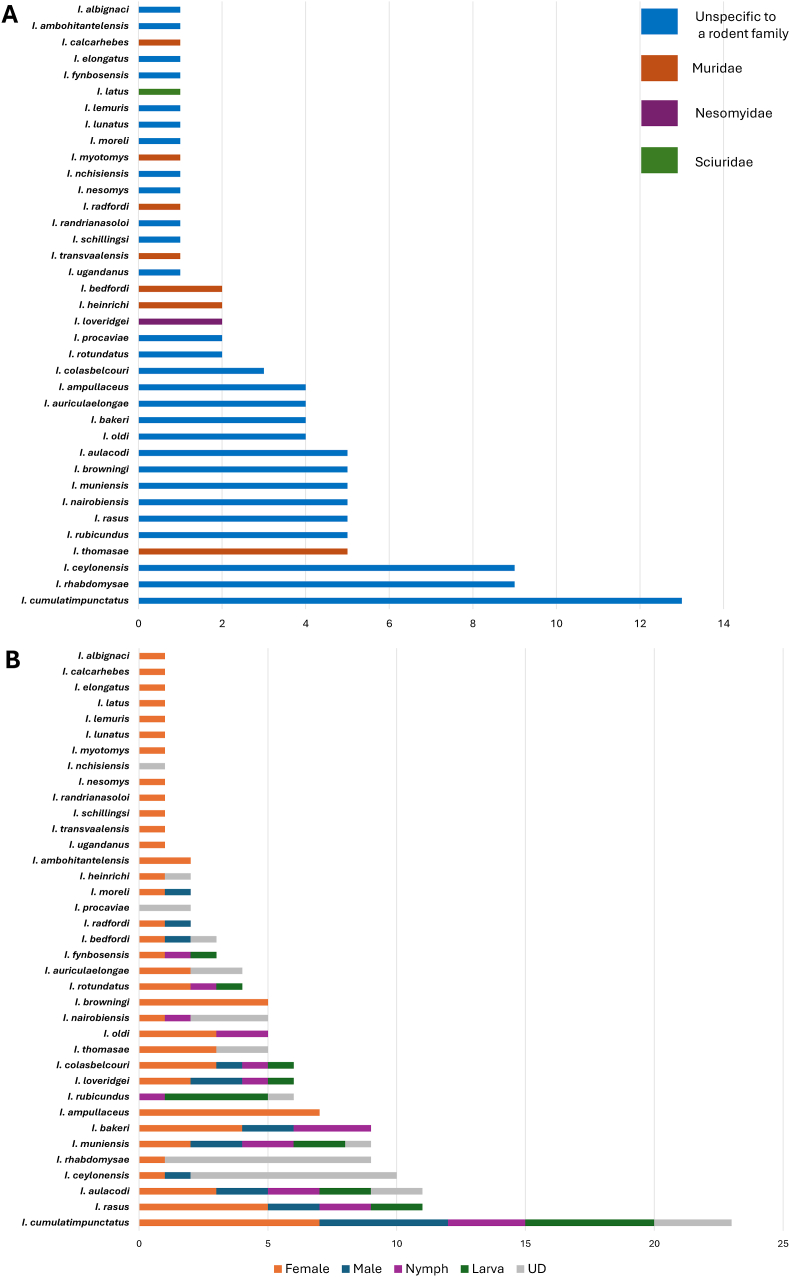
**A** Bar chart showing the number of rodent species parasitized by *Afrixodes* spp. *Ixodes minutae* is only known on an unidentified rodent and is not shown in this graph (see Supplementary Table S3). **B** Stacked bar chart of each life stage and/or sex of the *Afrixodes* spp. reported on each rodent host species. Details and references are available in Supplementary Table S3. *Abbreviation*: UD, undetermined stage.

Seven species of *Afrixodes* (*I*. *bedfordi*, *I. calcarhebes*, *I. heinrichi*, *I. myotomys*, *I. radfordi*, *I. thomasae*, and *I. transvaalensis*) were only found on hosts of the Muridae, one (*I. loveridgei*) is specific to hosts of the Nesomyidae, and another one (*I*. *latus*) is specific to hosts of the Sciuridae (Fig. 4A). Seventeen *Afrixodes* species were reported on a single rodent species (*I*. *albignaci*, *I. ambohitantelensis*, *I. calcarhebes*, *I. elongatus*, *I. fynbosensis*, *I. latus*, *I. lemuris*, *I. lunatus*, *I. moreli*, *I. myotomys*, *I. nchisiensis*, *I. nesomys*, *I. radfordi*, *I. randrianasoloi*, *I. schillingsi*, *I. transvaalensis*, and *I. ugandanus*), five - on two rodent species (*I*. *bedfordi*, *I. heinrichi*, *I. loveridgei*, *I. procaviae*, and *I. rotundatus*), one - on three rodent species (*I*. *colasbelcouri*), four - on four rodent species (*I*. *ampullaceus*, *I. auriculaelongae*, *I. bakeri*, and *I. oldi*), seven - on five rodent species (*I*. *aulacodi*, *I. browningi*, *I. muniensis I. nairobiensis*, *I. rasus*, *I. rubicundus*, and *I. thomasae*) and two - on nine rodent species (*I*. *ceylonensis* and *I. rhabdomysae*) (Fig. 4A). *Ixodes cumulatimpunctatus* was the most represented species in the literature for Rodentia with 13 species of rodent parasitized (Fig. 4A), with adult females being the most observed life stage and sex (Fig. 4B).

In terms of strict host specificity, among the seven species found only on hosts of the Muridae, there was a close association between *I. calcarhebes* and *Mastomys natalensis*, *I. myotomys* and *Otomys unisulcatus*, *I. radfordi* and *Rattus rattus*, *I. transvaalensis* and *Aethomys namaquensi*, and *I. ambohitantelensis* and *Mus musculus* (Supplementary Tables S3 and S4). A close specific host-parasite relationship is noted between *I. latus* and *Sciurus lucifer* (Sciuridae), *I. muniensis* and *Graphiurus microtis* (Gliridae), *I. schillingsi* and *Lophiomys imhausi* (Cricetidae) (Supplementary Tables S3 and S4). Moreover, *I. loveridgei* has specific hosts from the family Nesomyidae. *Ixodes ugandanus* appears to be a generalist species for rodents (Muridae and Thryonomydae), and *I. ampullaceus* was found on hosts of the families Nesomyidae and Thryonomyidae (Supplementary Tables S3 and S4).

Of the 37 species of ticks, it is the females that were most commonly observed, with 70 out of 164 reports (43%), followed by males (20/164, 12%), nymphs (20/164,12%), larvae (19/164, 12%) and 21% with undetermined stage (Fig. 4B). Only six species of ticks were observed at all stages of their development on 37 species of rodents (*I*. *aulacodi*, *I. colasbelcouri*, *I. cumulatimpunctatus*, *I. loveridgei*, *I. muniensis*, and *I. rasus*) (Fig. 4B). The female stage was observed in 15 species of ticks, and two species of ticks (*I*. *nchisiensis* and *I. procaviae*) do not have a specific stage identified in the records (Fig. 4B and Supplementary Tables S3 and S4).

#### 3.5.2 Shrews (Afrosoricida, Soricomorpha, and Macroscelidea)

**Fig. 5:**
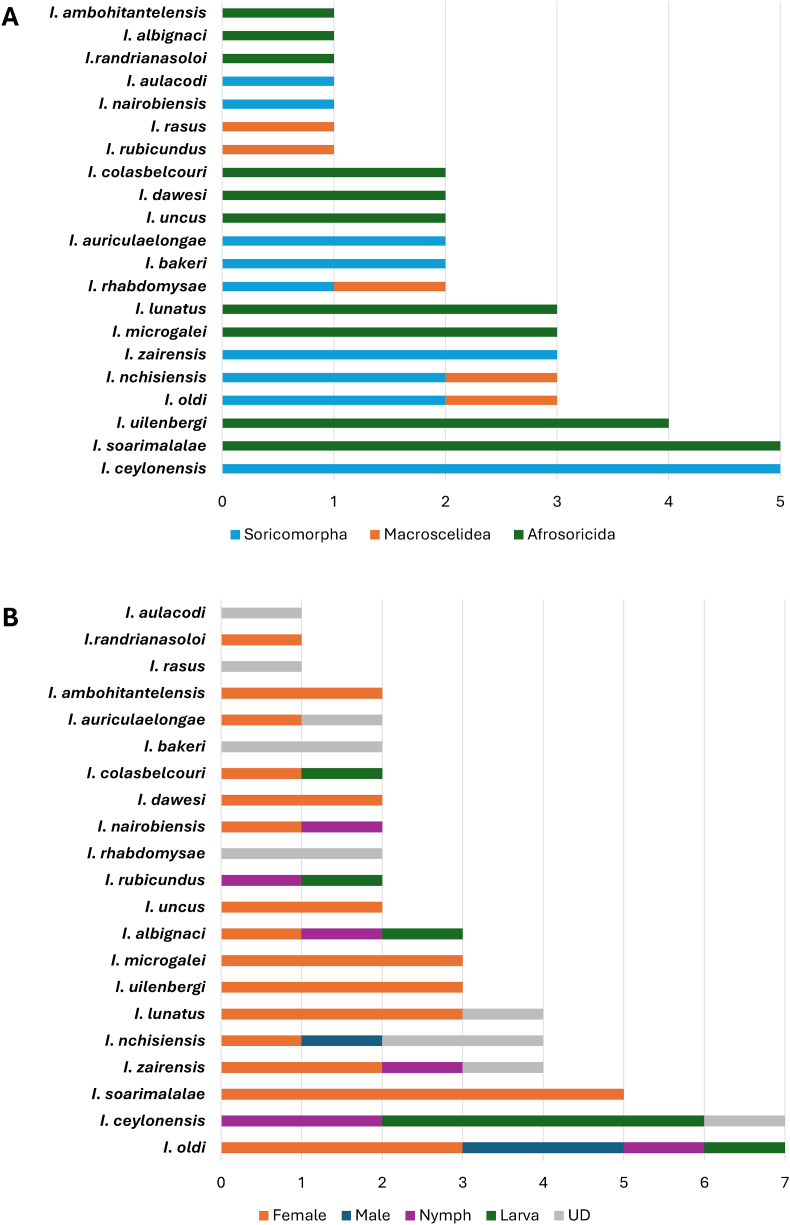
**A** Stacked bar chart representing the number of shrew species parasitized by *Afrixodes* spp. according to their clade (taxonomic order). **B** Stacked bar chart of each life stage and/or sex of the *Afrixodes* spp. reported on each shrew host. Details and references are provided in Supplementary Table S3. *Abbreviation*: UD, undetermined stage.

Among the 21 tick species reported in shrews, 18.9% were found on hosts of a single order, and tick species shared hosts of the three orders. Ten *Afrixodes* species (*I*. *ambohitantelensis*, *I. albignaci*, *I. colasbelcouri*, *I. dawesi*, *I. lunatus*, *I. microgalei*, *I. randrianasoloi*, *I. soarimalalae*, *I. uilenbergi*, and *I. uncus*) were found to exhibit a close relationship with hosts of the order Afrosoricida (Fig. 5A). A specific host-parasite association exists between species of the family Tenrecidae in Madagascar and the nine species of ticks (*I*. *albignaci*, *I. ambohitantelensis*, *I. colasbelcouri*, *I. lunatus*, *I. microgalei*, *I. randrianasoloi*, *I. soarimalalae*, *I. uilenbergi*, and *I. uncus*) endemic to the island (Table 1, Supplementary Tables S3 and S4).

Regarding the family Potamogalidae (representing the order Afrosoricida on the African continent), only *I. dawesi* was reported to parasitize hosts of this family (Supplementary Tables S3 and S4). Three *Ixodes* spp. (*I*. *nchisiensis*, *I. oldi*, and *I. rhabdomysae*) although restricted to the different shrew orders, were found on several host species within Macroscelidea and Soricomorpha, whereas six species (*I*. *aulacodi*, *I. auriculaelongae*, *I. bakeri*, *I. ceylonensis*, *I. nairobiensis*, and *I. zairensis*) and two species (*I*. *rasus* and *I. rubicundus*) were only found on hosts of the Soricomorpha and Macroscelidea, respectively (Fig. 5A–Supplementary Table S4). *Ixodes zairensis* was found exclusively on Soricomorpha, all mammals combined (Supplementary Tables S3 and S4).

Regarding the number of host species, eight species of *Afrixodes* were reported from two or more host species of the same order (Afrosoricida: seven species, i.e. *I*. *colasbelcouri*, *I. dawesi*, *I. lunatus*, *I. microgalei*, *I. uilenbergi*, *I. uncus*, and *I. soarimalalae*; Soricomorpha: four species, i.e. *I*. *auriculaelongae*, *I. bakeri*, *I. ceylonensis*, and *I. zairensis*) or of different orders (Soricomorpha and Macroscelidea: three species, i.e. *I*. *nchisiensis*, *I. oldi*, and *I. rhabdomysae*) (Fig. 5A, Supplementary Table S4). Three tick species were reported in more than three host species: *I*. *uilenbergi* in four species of the Afrosoricida; *I. soarimalalae* in five species of the Afrosoricida; and *I. ceylonensis* in five species of the Soricomorpha (Fig. 5A, Supplementary Table S4).

Females were the most frequently reported in the studies identifying life stage and sex (31 out of 61 reports, 51%). The male stage was reported in 5% (3/61) of the publications. Nymph stage and larvae were reported 11% (7/61) and 13% (8/61) of the publications, respectively (Fig. 5B). Female ticks alone were reported for seven species (*I*. *ambohitantelensis*, *I. dawesi*, *I. microgalei*, *I. randrianasoloi*, *I. soarimalalae*, *I. uilenbergi*, and *I. uncus*) (Fig. 5B). Only *I. nchisiensis* and *I. oldi* have the male stage identified on a host (Fig. 5B). Six *Afrixodes* species (*I*. *albignaci*, *I. ceylonensis*, *I. nairobiensis*, *I. oldi*, *I. rubicundus*, and *I. zairensis*) have been found at the nymph stage and five (*I*. *albignaci*, *I. ceylonensis*, *I. colasbelcouri*, *I. oldi*, and *I. rubicundus*) at the larval stage (Fig. 5B). *Ixodes rubicundus* and *I. ceylonensis* were reported from shrews only for immature stages (Fig. 5B). All stages and both sexes were observed on shrews only for *I. oldi* (Fig. 5B). Finally, some life cycle stages were not determined (UD) for nine species (*I*. *aulacodi*, *I. auriculaelongae*, *I. bakeri*, *I. ceylonensis*, *I. lunatus*, *I. nchisiensis*, *I. rasus*, *I. rhabdomysae*, and *I. zairensis*); of these, no other life cycle stages were determined for four species (*I*. *aulacodi*, *I. bakeri*, *I. rasus*, and *I. rhabdomysae*) (Fig. 5B).

### 3.6 Anecdotal data

Interestingly, in insular systems, other than islands cited here, such as Madagascar and Malta, we found two reports of *Afrixodes* spp.: one adult of *I. cumulatimpunctatus* found on a rodent on an island in Equatorial Guinea; and *I. schillingsi* collected on a brown greater galago, *Otolemur crassicaudatus*, on Pemba Island, Zanzibar (Supplementary Tables S3 and S4). Finally, *I. rasus* was reported on the forest hinge-back tortoise, *Kinixys erosa*, in Gabon (Supplementary Tables S3 and S4); this is the only report of an *Afrixodes* tick species on a reptile.

### 3.7 Species of *Afrixodes* as vectors of tick-borne pathogens or toxins

Although not specifically addressed in the systematic review, some of the reviewed articles (*n* = 7) were related to TBPs (*n* = 5) and toxins (*n* = 2). Among the 66 known species of *Afrixodes*, there were only seven species related to tick-borne diseases, four with a demonstrated role as vectors for a given pathogen (*I*. *aulacodi*, *I. colasbelcouri*, *I. muniensis*, and *I*. *rasus*) and three putative vectors (*I. ceylonensis*, *I. pilosus*, and *I. rubicundus*) (Table 2). As vectors, two species of *Afrixodes* can harbour and transmit two different TBP groups: *Ixodes aulacodi* (*Anaplasma* spp. and *Ehrlichia* spp.) and *I. muniensis* (*Babesia* spp. and *Rickettsia* spp.) (Table 2), and two other species have been reported regarding one TBP group: *Ixodes colasbelcouri* and *I. rasus* for *Rickettsia* spp. (Table 2). As putative vectors, *I. pilosus* and *I. rubicundus* may transmit *Anaplasma* spp., *Babesia* spp. and *Hepatozoon* spp., while *Rickettisa* spp. may be transmitted by *I. ceylonensis* (Table 2). For both types of vectors, most of the reported host-pathogen associations originate from South Africa (6; 46% of the records), followed by Uganda (3; 23%), Ghana (2; 15%), Madagascar and Sri Lanka (1; 8% each).

**Table 2:** Tick-borne pathogens recorded from species of *Afrixodes* in the articles included in the review.

Tick-borne pathogen	*Afrixodes* vector species	Distribution (country)	Reference
*Anaplasma* spp.	*I*. (*A.*) *aulacodi*	Ghana	Adenyo et al. (2020)
	*I*. (*A*.) *pilosus*^a^	South Africa	Viljoen et al. (2021)
	*I*. (*A.*) *rubicundus*^a^	South Africa	Viljoen et al. (2021)
*Babesia* spp.	*I*. (*A.*) *muniensis*	Uganda	Lacroux et al. (2023)
	*I*. (*A.*) *pilosus*^a^	South Africa	Viljoen et al. (2021)
	*I*. (*A.*) *rubicundus*^a^	South Africa	Viljoen et al. (2021)
*Ehrlichia* spp.	*I*. (*A.*) *aulacodi*	Ghana	Adenyo et al. (2020)
*Hepatozoon* spp.	*I*. (*A.*) *pilosus*^a^	South Africa	Viljoen et al. (2020, 2021)
	*I*. (*A.*) *rubicundus*^a^	South Africa	Viljoen et al. (2020, 2021)
*Rickettsia* spp.	*I*. (*A.*) *ceylonensis*^a^	Sri Lanka	Yamathramullage et al. (2018)
	*I*. (*A.*) *colasbelcouri*	Madagascar	Rasoamalala et al. (2022)
	*I*. (*A.*) *muniensis*	Uganda	Lacroux et al. (2023)
	*I*. (*A.*) *rasus*	Uganda	Lacroux et al. (2023)

a Putative vector.

Other than TBPs, an important disease, Karoo paralysis, restricted to South Africa and impacting livestock, is induced by a toxin found in female *I. rubicundus* saliva (Horak et al., 1987; Tonetti et al., 2009).

## 4 Discussion

The systematic review summarized the data for 66 species of the *Ixodes* subgenus *Afrixodes* and revealed (i) a large variety of hosts associated with *Afrixodes* spp., including more than 200 mammal and almost 30 bird species; (ii) 46% of the records from mammals were for micromammals, with 21 and 37 *Afrixodes* spp. reported on shrews and rodents, respectively; (iii) records for some TBPs (*Anaplasma* spp., *Babesia* spp., *Ehrlichia* spp., *Hepatozoon* spp. and *Rickettsia* spp.) associated with seven *Afrixodes* spp., acting as either competent or putative vectors.

The number of species of the subgenus *Afrixodes* considered valid is not yet clearly resolved. An exhaustive list of 60 species has been proposed by Camicas et al. (1998). Since then, nine additional species have been described or reported in the Afrotropical region: *I*. *ambohitantelensis*, *I. ampullaceus*, *I. colboi*, *I. fynbosensis*, *I. hyracis*, *I. microgalei*, *I. soarimalalae*, *I. uilenbergi*, and *I. uncus* (Apanaskevich et al., 2011, 2013, 2025a, 2025b; Apanaskevich and Goodman, 2020; Apanaskevich and Hall, 2025; Englert et al., 2023). The taxonomy of some *Afrixodes* species is a subject of ongoing debate. The taxonomic status of *I. donarthuri* Santos Dias, 1980 is not clearly defined; *I. donarthuri* was considered a valid taxon by Camicas et al. (1998) and by Barker and Murrell (2004) but not by Horak et al. (2002). Keirans and Hillyard (2001) examined a paratype of *I. donarthuri* and concluded that this name is a junior synonym of *I. neitzi* Clifford, Walker & Keirans, 1977. Finally, Guglielmone et al. (2020) did not consider *I. donarthuri* as a valid specie*s*. Robbins et al. (2025) recognized *I*. *ambohitantelensis*, *I. soarimalalae*, *I. uilenbergi*, and *I. uncus*, but did not validate *I. ampullaceus* (syn. *I. amersoni* Kohls, 1966) or *I. donarthuri* (syn. *I. domerguei* Uilenberg & Hoogstraal, 1965). Although included in the list of articles selected using the keyword search, the article on *I*. *hoogstraali* by Arthur (1954) was ultimately not used, as the validity of the species is disputed by other authors. *Ixodes hoogstraali* Arthur, 1954 was described at the female stage on a *Meriones rex brunei* (Muridae) in Yemen. Arthur (1954) and Morel (1966) considered that this species is a member of the subgenus *Afrixodes*, but no other mentions of this species, e.g. in Clifford et al. (1973), Guglielmone et al. (2020) or Robbins et al. (2025), indicate this possible assignment. The affiliation of *I. hoogstraali* to *Afrixodes* is therefore questionable, and this species has not been included in the review.

Another problem is the complex history of taxonomic and nomenclatural changes for some species, see e.g. Apanaskevich et al. (2025b), who redescribed *I. ampullaceus* Warburton, 1933 (syns *I. mossambicensis* Santos Dias, 1952; *I. mossambicus* Theiler, 1962 (*lapsus*)), *I. ugandanus* (syn. *Ixodes ugandanus ugandanus* Neumann, 1906 *sensu* Neumann, 1907), and *I. aulacodi*, and provided detailed comments on the identification and distribution of these species in Africa. Overall, the *Afrixodes* species number ranges between 60 and 69 species. Therefore, our review updating the nomenclature of both tick and host species may serve as an accurate portrayal of the true diversity of the 66 currently considered valid *Afrixodes* spp. in Africa, focusing on micromammals.

To our knowledge, this is the first review of *Afrixodes* species to examine both their geographical distribution and the hosts from which they were collected. The distribution range of species in the subgenus *Afrixodes* corresponds closely to the Afrotropical region of Africa, except for Egypt (Fig. 2), where a single species (*I. euplecti*) is reported (Table 1). *Afrixodes* ticks are frequently found associated with mountain forests and more specifically in rainforests or in savannahs near these forests (Morel, 1969). Species of *Afrixodes* have been reported on a wide range of hosts, representing more than 200 species (see Supplementary Table S4). One of the important results of this review is that species of the subgenus *Afrixodes* have a wider host range than previously thought. For example, in his extensive review of the tick-mammal hosts associations Kolonin (2007) listed four *Afrixodes* spp. associated with hosts of the Afrosoricida (*vs* 10 in the present review), one associated with hosts of the Soricomorpha (*vs* 9 in the present review), and one associated with hosts of the Macroscelidea (*vs* 5 in the present review). Tick host specificity is a major life history trait to characterize the ecology of a species and has some consequences on the potential role of this species as a vector for pathogens (McCoy et al., 2013). Our data show that a tick species could be reported on one or many hosts, belonging to several orders or families (see Supplementary Table S4) depending on the availability of hosts in the area (Estrada-Peña et al., 2023). The association of *Ixodes* spp. with vertebrate hosts is based on ecological factors.

Rodents (Rodentia) and shrews (Afrosoricida, Soricomorpha, and Macroscelidea) represent 46% of the host species recorded in the studies reviewed here (Fig. 3A), with 21 species of *Afrixodes* on shrews and 37 species on rodents (Supplementary Tables S3 and S4).

The role of small mammals as hosts of *Ixodes* spp. in the Palaearctic or Nearctic is well documented, particularly for immature stages (Horak et al., 2005; Paziewska et al., 2010; Shiels et al., 2014). Because of the difficulty of catching micromammals in the field, this frequent association of *Afrixodes* with rodents may be underestimated. In tropical regions, the density of vegetation can make it difficult to find and capture micromammals and thus their associated parasites. Nonetheless, these difficulties do not prevent the discovery of new species. For example, in Madagascar, there have been 339 newly discovered land vertebrates alone in the last 20 years, along with 24 new species of ixodid ticks out of the 27 known (Goodman, 2022). Madagascar is a biodiversity hotspot with an endemic rate of 89% for land vertebrates (Goodman, 2022); accordingly, there are 11 endemic *Afrixodes* species associated with endemic tenrecs or primates of Madagascar.

Micromammals are hosts found in the ticks’ environment, enabling the ticks to complete their entire life cycle. It is difficult to say whether micromammals are indeed the main suitable reproduction host for *Afrixodes* adults. However, if this is the case, they would be a key factor in limiting populations and species in the Afrotropical ecosystems. The spread of ticks is exacerbated by bird hosts, with species such as *I. euplecti* found on an *Anthus trivialis*, a migratory passerine. Detected in Egypt, this passerine migrates to Europe and specimens found in Malta were also found to host *I. cumulatimpunctatus* (Supplementary Table S3). Ticks are also spread by bovids during cross-border transhumance (Ouredraogo et al., 2021). These findings demonstrate the importance and the impact that migratory birds and human activities can have on the passive dispersal of ticks along their route in Europe, especially if they share their ecological niche with rodents and shrews.

Interestingly, our results include eight species reported from humans (without confirmation of engorgement): *I. cavipalpus* (Cumming, 1998), *I. cumulatimpunctatus* (Arthur and Burrow, 1957; Elbl and Anastos, 1966; Aeschlimann, 1967; Cumming, 1998), *I. muniensis* (Aeschlimann, 1967; Lacroux et al., 2023), *I. pilosus* (Cumming, 1998; Viljoen et al., 2020), *I. rageaui* (Morel and Mouchet, 1965), *I. rasus* (Aeschlimann, 1967; Lacroux et al., 2023), *I. schillingsi* (Hoogstraal and Theiler, 1959; Guglielmone et al., 2020) and *I. vanidicus* (Guglielmone et al., 2020). Micromammals such as rodents are present in both human and domestic animal habitats and constitute a bridge between wildlife and domestic animals in the transmission of TBPs (Tonetti et al., 2009; McCoy et al., 2013).

Some tick species that infest micromammals are important vectors of disease or toxins to domestic livestock, whereas others are of little or unknown economic importance. The pathogenic role of these ticks on livestock is due to blood spoliation, blood contamination, allergic reactions, superinfection, etc., and the indirect role through the transmission of bacteria, protozoa and viruses.

The TBPs *Anaplasma* spp., *Babesia* spp., *Ehrlichia* spp., *Hepatozoon* spp., and *Rickettsia* spp. reported from species of *Afrixodes* in the publications included in the review are not specific to this subgenus. Hosts that carry these microorganisms include African caracals (*Caracal caracal* and *C. mesomelas*) (Viljoen et al., 2020, 2021), Malagasy rodents (Rasoamalala et al., 2022), the greater cane rat (or grasscutter) *Thryonomys swinderianus* (Rodentia) from West Africa (Adenyo et al., 2020), chimpanzee (Lacroux et al., 2023), cattle (Santos et al., 2004; Vorou et al., 2007; de la Fuente et al., 2008; Parodi et al., 2022) as well as horses (Dziegiel et al., 2013), dogs (Skotarczak, 2003; Keyte et al., 2021) and humans (Portillo et al., 2015; Del Cerro et al., 2022).

Our review has some limitations. First, the search approach excluded references that do not mention host species, or report only ticks on vegetation, in nests, etc. Therefore, our review does not reflect all the data available on the geographical distribution of *Afrixodes* spp.; with a focus on host data, some countries have been reported in references excluded from the review. The distribution area of some species is thus broader than that reported here, i.e. *I*. *aulacodi* has also been reported in Burkina Faso, *I*. *pilosus* in Eswatini, *I*. *aulacodi*, *I. moreli*, *I. muniensis*, *I. rasus*, and *I. ugandanus* in Nigeria, *I*. *cumulatimpunctatus* and *I. rasus* in Senegal, and *Ixodes oldi* and *I. rasus* in Togo (Guglielmone et al., 2023). Secondly, some reports may have been missed, and some were not accessible online. Despite these limitations, the review was based on a substantial amount of studies, ensuring the near-complete picture of the diversity and distribution of *Afrixodes* spp. in Africa, focusing on micromammals.

## 5 Conclusions

This review provides a complete list of the 66 species of *Afrixodes* and an overview of the distribution of these species, as well as tick-host and tick-host-pathogen associations. The data gathered highlight the great diversity of host species, more than 200 mammal species and more than 20 bird species. Micromammals are important hosts for *Afrixodes* species, which share the same ecological niches with many species of ticks. Furthermore, all stages of tick development are present in these hosts, making them a key host in the ecology of *Afrixodes* species. Finally, this systematic review compiled data for four species as competent vectors and three as putative vectors of vector-borne pathogens.

## Ethical approval

Not applicable.

## CRediT authorship contribution statement

**Camille Lorang:** Conceptualization, Methodology, Formal analysis, Writing - original draft, Writing - review & editing. **Denis Augot:** Writing - original draft, Writing - review & editing.

## Funding

This work was supported by the Ile-de-France region, France through the DIM OneHealth.

## Declaration of competing interests

The authors declare that they have no known competing financial interests or personal relationships that could have appeared to influence the work reported in this paper.

## Data Availability

The data supporting the conclusions of this article are included within the article and its supplementary files.
