# Supplementary material for: Ecological review of Afrotropical Ixodes (Afrixodes) ticks: Distribution and host diversity, with a focus on micromammals
Source: Curr Res Parasitol Vector Borne Dis. 2026 May 27;9:100392. doi: 10.1016/j.crpvbd.2026.100392 (PMC13266215; doi:10.1016/j.crpvbd.2026.100392)
Supplement: Multimedia component 1 [file mmc1.pdf]

Supplementary Table 1. PRISMA checklist.

Section and Topic	Item #	Checklist item	Location where item is reported
**TITLE**			
Title	1	Identify the report as a systematic review.	1
**ABSTRACT**			
Abstract	2	See the PRISMA 2020 for Abstracts checklist.	1
**INTRODUCTION**			
Rationale	3	Describe the rationale for the review in the context of existing knowledge.	2-3
Objectives	4	Provide an explicit statement of the objective(s) or question(s) the review addresses.	3
**METHODS**			
Eligibility criteria	5	Specify the inclusion and exclusion criteria for the review and how studies were grouped for the syntheses.	4
Information sources	6	Specify all databases, registers, websites, organisations, reference lists and other sources searched or consulted to identify studies. Specify the date when each source was last searched or consulted.	4
Search strategy	7	Present the full search strategies for all databases, registers and websites, including any filters and limits used.	4-5
Selection process	8	Specify the methods used to decide whether a study met the inclusion criteria of the review, including how many reviewers screened each record and each report retrieved, whether they worked independently, and if applicable, details of automation tools used in the process.	4-5
Data collection process	9	Specify the methods used to collect data from reports, including how many reviewers collected data from each report, whether they worked independently, any processes for obtaining or confirming data from study investigators, and if applicable, details of automation tools used in the process.	4-5
Data items	10a	List and define all outcomes for which data were sought. Specify whether all results that were compatible with each outcome domain in each study were sought (e.g. for all measures, time points, analyses), and if not, the methods used to decide which results to collect.	4-5
	10b	List and define all other variables for which data were sought (e.g. participant and intervention characteristics, funding sources). Describe any assumptions made about any missing or unclear information.	4-5
Study risk of bias assessment	11	Specify the methods used to assess risk of bias in the included studies, including details of the tool(s) used, how many reviewers assessed each study and whether they worked independently, and if applicable, details of automation tools used in the process.	4
Effect measures	12	Specify for each outcome the effect measure(s) (e.g. risk ratio, mean difference) used in the synthesis or presentation of results.	NA
Synthesis methods	13a	Describe the processes used to decide which studies were eligible for each synthesis (e.g. tabulating the study intervention characteristics and comparing against the planned groups for each synthesis (item #5)).	4-5, Fig. 1
	13b	Describe any methods required to prepare the data for presentation or synthesis, such as handling of missing summary statistics, or data conversions.	NA
	13c	Describe any methods used to tabulate or visually display results of individual studies and syntheses.	4-5
	13d	Describe any methods used to synthesize results and provide a rationale for the choice(s). If meta-analysis was performed, describe the model(s), method(s) to identify the presence and extent of statistical heterogeneity, and software package(s) used.	NA

Section and Topic	Item #	Checklist item	Location where item is reported
	13e	Describe any methods used to explore possible causes of heterogeneity among study results (e.g. subgroup analysis, meta-regression).	NA
	13f	Describe any sensitivity analyses conducted to assess robustness of the synthesized results.	NA
Reporting bias assessment	14	Describe any methods used to assess risk of bias due to missing results in a synthesis (arising from reporting biases).	4
Certainty assessment	15	Describe any methods used to assess certainty (or confidence) in the body of evidence for an outcome.	4
**RESULTS**			
Study selection	16a	Describe the results of the search and selection process, from the number of records identified in the search to the number of studies included in the review, ideally using a flow diagram.	5
	16b	Cite studies that might appear to meet the inclusion criteria, but which were excluded, and explain why they were excluded.	5
Study characteristics	17	Cite each included study and present its characteristics.	5, Fig. 1, Supplementary Table S3
Risk of bias in studies	18	Present assessments of risk of bias for each included study.	5
Results of individual studies	19	For all outcomes, present, for each study: (A.) summary statistics for each group (where appropriate) and (b) an effect estimate and its precision (e.g. confidence/credible interval), ideally using structured tables or plots.	Supplementary Table S3
Results of syntheses	20a	For each synthesis, briefly summarise the characteristics and risk of bias among contributing studies.	Tables 1-3, Supplementary Table S4
	20b	Present results of all statistical syntheses conducted. If meta-analysis was done, present for each the summary estimate and its precision (e.g. confidence/credible interval) and measures of statistical heterogeneity. If comparing groups, describe the direction of the effect.	5-11, Fig 2, 3, 4, 5
	20c	Present results of all investigations of possible causes of heterogeneity among study results.	NA
	20d	Present results of all sensitivity analyses conducted to assess the robustness of the synthesized results.	NA
Reporting biases	21	Present assessments of risk of bias due to missing results (arising from reporting biases) for each synthesis assessed.	5
Certainty of evidence	22	Present assessments of certainty (or confidence) in the body of evidence for each outcome assessed.	NA
**DISCUSSION**			
Discussion	23a	Provide a general interpretation of the results in the context of other evidence.	11
	23b	Discuss any limitations of the evidence included in the review.	12
	23c	Discuss any limitations of the review processes used.	16, Table 3
	23d	Discuss implications of the results for practice, policy, and future research.	12-15
**OTHER INFORMATION**			

Section and Topic	Item #	Checklist item	Location where item is reported
Registration and protocol	24a	Provide registration information for the review, including register name and registration number, or state that the review was not registered.	NA
	24b	Indicate where the review protocol can be accessed, or state that a protocol was not prepared.	NA
	24c	Describe and explain any amendments to information provided at registration or in the protocol.	NA
Support	25	Describe sources of financial or non-financial support for the review, and the role of the funders or sponsors in the review.	18
Competing interests	26	Declare any competing interests of review authors.	18
Availability of data, code and other materials	27	Report which of the following are publicly available and where they can be found: template data collection forms; data extracted from included studies; data used for all analyses; analytic code; any other materials used in the review.	18

Supplementary Table S2. Listing of the 66 *Ixodes* (*Afrixodes*) species with indication of the life cycle stage and sex of the deposited in museums types according to Robbins et al. (2025), Englert et al. (2023) and Santos Dias (1952).

**Species of *Ixodes* (*Afrixodes*)**	**Stage/sex of deposited types**
I. (*A.*) *albignaci* Uilenberg & Hoogstraal, 1970	F
I. (*A.*) *ambohitantelensis* Englert, Goodman & Apanaskevich, 2023	F
I. (*A.*) *ampullaceus* Warburton, 1933	F, M
I. (*A.*) *arebiensis* Arthur, 1956	F
I. (*A.*) *aulacodi* Arthur, 1956	F
I. (*A.*) *auriculongae* Arthur, 1958	F
I. (*A.*) *bakeri* Arthur & Clifford, 1961	F
I. (*A.*) *bedfordi* Arthur, 1959	F, M
I. (*A.*) *brewsterae* Keirans, Clifford & Walker, 1982	F, M
I. (*A.*) *browningi* Arthur, 1956	F, M
I. (*A.*) *brumpti* Morel, 1965	F
I. (*A.*) *calcarhebes* Arthur & Zulu, 1980	F
I. (*A.*) *catherinei* Keirans, Clifford & Walker, 1982	F
I. (*A.*) *cavipalpus* Nuttall & Warburton, 1908	F, M
I. (*A.*) *ceylonensis* Kohls, 1950	F
I. (*A.*) *colasbelcouri* Arthur, 1957	F, M, N, L
I. (*A.*) *corwini* Keirans, Clifford & Walker, 1982	F, M, N
I. (*A.*) *cumulatimpinctatus* Schulze, 1943	F, M, N
I. (*A.*) *dawesi* Arthur, 1956	F
I. (*A.*) *djaronensis* Neumann, 1907	F, M
I. (*A.*) *drakensbergensis* Clifford, Theiler & Baker, 1975	F, M, N, L
I. (*A.*) *elongatus* Bedford, 1929	F
I. (*A.*) *euplecti* Arthur, 1958	F
I. (*A.*) *evansi* Arthur, 1956	F, M
I. (*A.*) *fynbosensis* Apanaskevich, Horak, Matthee & Matthee, 2011	F, N, L
I. (*A.*) *heinrichi* Arthur, 1962	F, M
I. (*A.*) *hyracis* Apanaskevich, Drew & Pienaar, 2025	F
I. (*A.*) *latus* Arthur, 1958	F
I. (*A.*) *lemuris* Arthur, 1957	F
I. (*A.*) *lewisi* Arthur, 1965	F, M
I. (*A.*) *loveridgei* Arthur, 1958	F, M
I. (*A.*) *lunatus* Neumann, 1907	F
I. (*A.*) *macfarlanei* Keirans, Clifford & Walker, 1982	F, M
I. (*A.*) *matopi* Spickett, Keirans, Norval & Clifford, 1981	F, M
I. (*A.*) *microgalei* Apanaskevich, Soarimalala & Goodman, 2013	F
I. (*A.*) *minutae* Arthur, 1959	F
I. (*A.*) *moreli* Arthur, 1957	F
I. (*A.*) *muniensis* Arthur & Burrow, 1957	F, N, L
I. (*A.*) *myotomys* Clifford & Hoogstraal, 1970	F
I. (*A.*) *nairobiensis* Nuttall, 1916	F
I. (*A.*) *nchisiensis* Arthur, 1958	F, M
I. (*A.*) *neitzi* Clifford, Walker & Keirans, 1977	F, M
I. (*A.*) *nesomys* Uilenberg & Hoogstraal, 1969	F

**Species of *Ixodes* (*Afrixodes*)**	**Stage/sex of deposited types**
I. (*A.*) *nicolasi* Santos Dias, 1982	F
I. (*A.*) *okapiae* Arthur, 1956	F
I. (*A.*) *oldi* Nuttall, 1913	F, M
I. (*A.*) *pilosus* Koch, 1844	F
I. (*A.*) *procaviae* Arthur & Burrow, 1957	F, M
I. (*A.*) *radfordi* Kohls, 1948	F
I. (*A.*) *rageaui* Arthur, 1957	F, M
I. (*A.*) *randrianasoloi* Uilenberg & Hoogstraal, 1969	F
I. (*A.*) *rasus* Neumann, 1899	F, M
I. (*A.*) *rhabdomysae* Arthur, 1959	F
I. (*A.*) *rotundatus* Arthur, 1958	F
I. (*A.*) *rubicundus* Neumann, 1904	F, M
I. (*A.*) *schillingsi* Neumann, 1901	F, N
I. (*A.*) *soarimalalae* Apanaskevich & Goodman, 2020	F
I. (*A.*) *spinae* Arthur, 1958	F, M
I. (*A.*) *thomasae* Arthur & Burrow, 1957	F
I. (*A.*) *transvaalensis* Clifford & Hoogstraal, 1966	F
I. (*A.*) *ugandanus* Neumann, 1906	F, M
I. (*A.*) *uilenbergi* Apanaskevich & Goodman, 2020	F
I. (*A.*) *uncus* Apanaskevich & Goodman, 2020	F
I. (*A.*) *vanidicus* Schulze, 1943	F, M, N, L
I. (*A.*) *walkerae* Clifford, Kohls & Hoogstraal, 1968	F
I. (*A.*) *zairensis* Keirans, Clifford & Walker, 1982	F, N

Abbreviations: F, female; L, larva; M, male; N, nymph.

Supplementary Table S3. Summary table of all hosts, mammals, birds, and reptile, found in the publications included in the review.

Order	Family	Host species (scientific name)	Host species (common name)	*Ixodes* (*Afrixodes*) species	Life stage	Locality	Reference
**Mammalia**							
**Afrosoricida**	Tenrecidae	*Hemicentetes nigriceps*	Highland streaked tenrec	*I. (A.) oarimalalae*	F	Madagascar	1
				I. (A.) uilenbergi	F	Madagascar	1
		Hemicentetes semispinosus	Lowland streaked tenrec	*I. (A.) lunatus*	F	Madagascar	2
		Microgale cowani	Cowan's shrew tenrec	*I. (A.) soarimalalae*	F	Madagascar	1
		Microgale majori	Major's shrew tenrec	*I. (A.) ambohitantelensis*	F	Madagascar	3
		Microgale parvula	Pygmy shrew tenrec	*I. (A.) microgalei*	F	Madagascar	4
		Microgale soricoides	Shrew-toothed shrew tenrec	*I. (A.) microgalei*	F	Madagascar	4
				I. (A.) soarimalalae	F	Madagascar	1
				I. (A.) uilenbergi	F	Madagascar	1
				I. (A.) uncus	F	Madagascar	1
		Nesogale dobsoni	Dobson's shrew tenrec	*I. (A.) microgalei*	F	Madagascar	1, 4
				I. (A.) soarimalalae	F	Madagascar	1
				I. (A.) uilenbergi	F	Madagascar	1
				I. (A.) uncus	F	Madagascar	1
		Nesogale talazaci	Talazac's shrew tenrec	*I. (A.) albignaci*	F, N, L	Madagascar	1, 2, 5
				I. (A.) colasbelcouri	L	Madagascar	2
				I. (A.) randrianasoloi	F	Madagascar	1, 2, 5
		Oryzorictes hova	Mole-like rice tenrec	*I. (A.) soarimalalae*	F	Madagascar	1
				I. (A.) uilenbergi	F	Madagascar	1
		Setifer setosus	Greater hedghog tenrec	*I. (A.) lunatus*	F	Madagascar	2, 75
		Tenrec sp.	Tenrec	*I. (A.) lunatus*	UD	Madagascar	1

Order	Family	Host species (scientific name)	Host species (common name)	*Ixodes* (*Afrixodes*) species	Life stage	Locality	Reference
		Tenrec ecaudatus	Tailless tenrec	*I. (A.) colasbelcouri*	F	Madagascar	2, 75
				I. (A.) lunatus	F	Madagascar	2, 75
	Potamogalidae	*Micropotamogale ruwenzorii*	Ruwenzori otter shrew	*I. (A.) dawesi*	F	DRC	6
		Potamogale velox	Giant otter shrew	*I. (A.) dawesi*	F	DRC	5, 6, 7
**Artiodactyla**	Bovidae	*Aepyceros melampus*	Impala (or rooibok)	*I. (A.) neitzi*	A	South Africa	5, 8, 9
		Alcelaphus buselaphus caama	Red hartebeest	*I. (A.) rubicundus*	F, M	South Africa	10, 11
		Alcelaphus lichtensteinii	Lichtenstein's hartebeest	*I. (A.) cavipalpus*	F, M	Tanzania	6
		Antidorcas marsupiali	Springbok	*I. (A.) procaviae*	UD	South Africa	11, ,12, 13
				I. (A.) rubicundus	F, M, N, L	South Africa	10, 11, 14, 15
		Bos indicus	Zebu	*I. (A.) rubicundus*	F, M	South Africa	15
		Bos taurus	Cattle	*I. (A.) cavipalpus*	F	South Africa	9, 16
				I. (A.) cumulatimpunctatus	F, M	DRC, Ivory Coast, Uganda	9, 17, 19
				I. (A.) drakensbergensis	M	South Africa	16
				I. (A.) muniensis	F	Ivory Coast	17
				I. (A.) oldi	F	Ivory Coast	9, 17
				I. (A.) pilosus	A	South Africa	9, 16, 18
				I. (A.) rubicundus	F, M	South Africa	15
		Capra hircus	Domestic goat	*I. (A.) cavipalpus*	A	DRC	6, 9
				I. (A.) cumulatimpunctatus	UD, F	Africa, DRC	9, 19
				I. (A.) drakensbergensis	F, M, N, L	South Africa	16
				I. (A.) lewisi	UD	Africa	9
				I. (A.) matopi	F	Zimbabwe	20
				I. (A.) muniensis	A, N	Ivory Coast, Rwanda	6, 9, 17
				I. (A.) pilosus	UD	Africa	9
				I. (A.) rasus	UD	Africa	9

Order	Family	Host species (scientific name)	Host species (common name)	*Ixodes* (*Afrixodes*) species	Life stage	Locality	Reference
				I. (A.) rubicundus	A	South Africa	9, 16, 21
				I. (A.) ugandanus	UD	Africa	9
			Dwarf goat	*I. (A.) rasus*	F	Ivory Coast	17
		Cephalophus dorsalis	Bay duiker	*I. (A.) cumulatimpunctatus*	A, N, L	Gabon	22, 23
				I. (A.) moreli	A	Ghana, Ivory Coast	17, 23, 24
				I. (A.) muniensis	A, N	Ghana, Ivory Coast	17, 24
				I. (A.) rasus	A, N, L	Gabon	9, 22
		Cephalophus dorsalis castaneus	Bay duiker	*I. (A.) muniensis*	F	Cameroon	25
		Cephalophus leucogaster	White-bellied duiker	*I. (A.) rasus*	F	Cameroon	9, 19
		Cephalophus niger	Black duiker	*I. (A.) aulacodi*	A, N	Ghana	23, 24
				I. (A.) cumulatimpunctatus	A, N	Ghana, Ivory Coast	17, 24, 26
				I. (A.) moreli	F	Ghana, Ivory Coast	17, 23, 24
				I. (A.) muniensis	A, N	Ghana, Ivory Coast	17, 23, 24
				I. (A.) oldi	F	Ivory Coast	9, 17
		Cephalophus nigrifrons nigrifrons	Black-fronted duiker	*I. (A.) cumulatimpunctatus*	F, N	Rwanda	6
				I. (A.) muniensis	F	Rwanda	6
				I. (A.) rasus	A, N, L	Gabon	9, 22
		Cephalophus rufilatus	Red-flanked duiker	*I. (A.) moreli*	A	Ghana	23
				I. (A.) muniensis	A	Ghana	24
		Cephalophus sp.	Duiker	*I. (A.) cavipalpus*	M	DRC	6
				I. (A.) cumulatimpunctatus	F, N	Equatorial Guinea, Kenya	9, 19, 25
				I. (A.) moreli	UD	Ivory Coast, Kenya	9, 21
				I. (A.) muniensis	F, M, N, L	DRC, Equatorial Guinea, Gabon, Ivory Coast, Rwanda	6, 9, 17, 19, 22, 25
		Cephalophus silvicultor	Yellow-backed duiker	*I. (A.) macfarlanei*	A	DRC	27, 28
		Cephalophus zebra	Zebra duiker	*I. (A.) cumulatimpunctatus*	F	Ivory Coast	17
				I. (A.) moreli	F	Ivory Coast	17

Order	Family	Host species (scientific name)	Host species (common name)	*Ixodes* (*Afrixodes*) species	Life stage	Locality	Reference
		Connochaetes gnou	Black wildebeest	*I. (A.) procaviae*	UD	South Africa	11, 12
				I. (A.) rubicundus	F, L	South Africa	14
		Damaliscus pygargus dorcas	Blesbok	*I. (A.) procaviae*	UD	South Africa	11, 12, 18
		Damaliscus pygargus phillipsi	Blesbok	*I. (A.) rubicundus*	A	South Africa	9, 10, 11
		Neotragus batesi	Bate's pygmy antelope	*I. (A.) muniensis*	A, N	Cameroon	9, 25
				I. (A.) rasus	N	Cameroon	9, 25
		Neotragus moschatus akeleyi	Suni	*I. (A.) cumulatimpunctatus*	F	Kenya	19
		Neotragus pygmaeus	Royal antelope	*I. (A.) aulacodi*	A, N, L	Ghana	23, 24
				I. (A.) cumulatimpunctatus	N	Ivory Coast	9, 17, 26
				I. (A.) moreli	A	Ghana	9, 24
				I. (A.) muniensis	A, N	Ghana, Ivory Coast	9, 17, 19, 23, 24, 26
				I. (A.) rasus	F, N	DRC, Ivory Coast	9, 17, 19
		Oreotragus oreotragus	Klipspringer	*I. (A.) cavipalpus*	F	DRC	6, 7
				I. (A.) matopi	F, M	Zimbabwe	5, 9, 20
				I. (A.) neitzi	A	Zimbabwe	5, 9, 29
		Oryx gazella	Gemsbok	*I. (A.) procaviae*	UD	South Africa	11, 12
		Ourebia ourebi	Oribi	*I. (A.) cumulatimpunctatus*	UD	Ivory Coast	17
		Ovis aries	Domestic sheep	*I. (A.) cavipalpus*	A	DRC	6, 9
				I. (A.) cumulatimpunctatus	UD	Africa	9
				I. (A.) lewisi	UD	Africa	9
				I. (A.) muniensis	N, A	Ivory Coast	9, 17
				I. (A.) pilosus	UD	Africa	9
				I. (A.) rasus	UD	Africa	9
				I. (A.) rubicundus	F, M	South Africa	9, 11, 15, 16, 21, 30
				I. (A.) ugandanus	UD	Africa	9
		Pelea capreolus	Grey rhebok	*I. (A.) pilosus*	A, N, L	South Africa	8, 18

Order	Family	Host species (scientific name)	Host species (common name)	*Ixodes* (*Afrixodes*) species	Life stage	Locality	Reference
				I. (A.) procaviae	UD	South Africa	11, 12
		Philantomba maxwellii	Maxwell's duiker	*I. (A.) aulacodi*	A, N, L	Ghana	23, 24
				I. (A.) cumulatimpunctatus	A, N, L	Ghana, Ivory Coast	17, 23, 24
				I. (A.) moreli	A	Ghana	23, 24
				I. (A.) muniensis	A	Ghana, Ivory Coast	17, 23, 24, 26
				I. (A.) rasus	N	Ivory Coast	17
		Philantomba monticola	Blue duiker	*I. (A.) cumulatimpunctatus*	A, N	Gabon	22
				I. (A.) rasus	A, N, L	Gabon	22
		Philantomba sp.	Duiker	*I. (A.) cumulatimpunctatus*	N	Cameroon	25
				I. (A.) muniensis	A, N	Cameroon	25
		Redunca fulvorufula	Mountain reedbuck	*I. (A.) neitzi*	F, M	South Africa	5, 9, 31
				I. (A.) procaviae	UD	South Africa	11, 12
				I. (A.) rubicundus	F, M, L	South Africa	8, 9, 14
		Redunca sp.	Reedbuck	*I. (A.) pilosus*	UD	South Africa	9, 21
		Sylvicapra grimmia	Common duiker	*I. (A.) pilosus*	A, N, L	South Africa	8
		Syncerus caffer	African buffalo	*I. (A.) cumulatimpunctatus*	F	DRC	6, 19
		Taurotragus oryx	Common eland	*I. (A.) drakensbergensis*	F, M, N, L	South Africa	5, 16
				I. (A.) procaviae	UD	South Africa	5, 9, 11, 12
				I. (A.) rubicundus	F, M	South Africa	8, 9, 11, 15, 33
		Tragelaphus eurycerus	Bongo	*I. (A.) cumulatimpunctatus*	UD, F, N	Equatorial Guinea, Ivory Coast, Kenya	9, 17, 19
				I. (A.) muniensis	F, M, N	Ivory Coast, Equatorial Guinea	9, 17, 19
				I. (A.) rasus	F	Cameroon	9, 25
		Tragelaphus scriptus	Bushbuck	*I. (A.) aulacodi*	A, N, L	Ghana	23, 24
				I. (A.) cumulatimpunctatus	A, N, L	DRC, Ghana, Ivory Coast, Uganda	17, 19, 23, 24
				I. (A.) evansi	F	DRC	9
				I. (A.) moreli	F	Ivory Coast, Ghana	6, 17, 23

Order	Family	Host species (scientific name)	Host species (common name)	*Ixodes* (*Afrixodes*) species	Life stage	Locality	Reference
				I. (A.) muniensis	A	DRC, Ghana, Ivory Coast	6, 9, 17, 23, 24
				I. (A.) pilosus	A, N, L	South Africa	8
				I. (A.) rotundatus	F	Uganda	9, 35
		Tragelaphus sp.	Spiral-horned antelope	*I. (A.) cumulatimpunctatus*	UD	Africa	9, 21
				I. (A.) moreli	UD	Ivory Coast, Kenya	9, 21
				I. (A.) pilosus	UD	South Africa	9, 18, 21
				I. (A.) procaviae	F, M, N, L	South Africa	11, 13
				I. (A.) rubicundus	F, M	South Africa	11, 33
		Tragelaphus strepsiceros	Greater kudu	*I. (A.) rubicundus*	F, M	South Africa	10, 11, 30
		UD	Boviid	*I. (A.) ceylonensis*	A	India	5, 28
				I. (A.) latus	F	Africa	9
				I. (A.) nicolasei	UD	Mozambique	34
				I. (A.) vanidicus	A	Africa	9, 28
		UD Antilopinae	Antelope	*I. (A.) cavipalpus*	F, M	DRC	6
				I. (A.) lewisi	UD	Africa	9, 28
		UD Cephalophinae	Duiker	*I. (A.) lewisi*	UD	Africa	9
		UD Neotraginae	Dwarf antelopes including klipspringer, suni, grysbok	*I. (A.) lewisi*	UD	Africa	9
				I. (A.) matopi	UD	Africa	5, 9
		UD Reduncinae	Reedbuck, lechwe, waterbuck	*I. (A.) lewisi*	UD	Africa	9
		UD Tragelaphinae	Kudu, eland, sitatunga	*I. (A.) lewisi*	UD	Africa	9
	Giraffidae	*Okapia johnstoni*	Okapi	*I. (A.) okapiae*	F	DRC	5, 6, 9, 35
		Okapia sp.	Okapi	*I. (A.) muniensis*	A	Africa	6, 9
		UD	Girafe and okapi	*I. (A.) cavipalpus*	A	Africa	9
				I. (A.) ceylonensis	UD	India	36
				I. (A.) lewisi	UD	Africa	9
				I. (A.) rasus	UD	Africa	9

**Order**	**Family**	**Host species (scientific name)**	**Host species (common name)**	***Ixodes* (*Afrixodes*) species**	**Life stage**	**Locality**	**Reference**
	Suidae	*Phacochoerus aethiopicus*	Desert warthog	*I. (A.) nairobiensis*	F	Kenya	6, 9
		Potamochoerus porcus	Red river hog	*I. (A.) cumulatimpunctatus*	A, N, L	DRC, Gabon, Ghana, Ivory Coast	9, 17, 19, 22, 23, 24
				I. (A.) muniensis	F	DRC	6
				I. (A.) rasus	A	Gabon	9, 22
		Potamochoerus sp.	Bushpig	*I. (A.) cumulatimpunctatus*	F	Cameroon	9, 21, 25
				I. (A.) procaviae	F	Burundi	9, 19
				I. (A.) rasus	F	Cameroon	9, 19, 25
		Sus domesticus	Domestic pig	*I. (A.) cumulatimpunctatus*	UD	DRC	9, 37
				I. (A.) muniensis	A, N	Rwanda	6, 9
		UD	Wild pig	*I. (A.) muniensis*	A, N	Africa	9
	Trangulidae	*Hyemoschus aquaticus*	Water chevrotain (or fanged deer)	*I. (A.) cumulatimpunctatus*	F, N	Equatorial Guinea, Ivory Coast	9, 25, 26
				I. (A.) rasus	N	Ivory Coast	9, 17
		Tragulus sp.	Chevrotain (or mouse-deer)	*I. (A.) cumulatimpunctatus*	F, N	Equatorial Guinea	19
**Carnivora**	Canidae	*Canis familiaris*	Domestic dog	*I. (A.) cavipalpus*	A	Africa	9
				I. (A.) corwini	F, M	South Africa	18, 27
				I. (A.) cumulatimpunctatus	A, N, L	DRC, Gabon, Ivory Coast	9, 17, 19 22, 26
				I. (A.) fynbosensis	F	Afrique du sud	38
				I. (A.) lewisi	UD	Africa	9
				I. (A.) muniensis	A, N	Ivory Coast	9, 17, 26
				I. (A.) nairobiensis	F	Kenya	6, 9, 39
				I. (A.) oldi	F	Ivory Coast	9, 17, 21
				I. (A.) pilosus	A	South Africa	9, 16, 18
				I. (A.) procaviae	F, M, N, L	South Africa	18
				I. (A.) rasus	F, N	Cameroon, Ivory Coast	9, 17, 25
				I. (A.) rubicundus	A	Africa	9

Order	Family	Host species (scientific name)	Host species (common name)	*Ixodes* (*Afrixodes*) species	Life stage	Locality	Reference
				I. (A.) vanidicus	A	Cameroon	9, 25, 28
		Lupulella mesomelas	Black-backed jackal	*I. (A.) pilosus*	A, N, L	South Africa	8
				I. (A.) procaviae	F, M, N, L	South Africa	18
				I. (A.) rubicundus	F, M	South Africa	11, 40
		Otocyon megalotis	Bat-eared fox	*I. (A.) pilosus*	A, N, L	South Africa	8
				I. (A.) procaviae	L	South Africa	18
	Felidae	*Caracal caracal*	Caracal	*I. (A.) pilosus*	A, N, L	South Africa	8
				I. (A.) procaviae	UD	South Africa	40, 41
				I. (A.) rubicundus	F, M, L	South Africa	8, 11, 14, 33
		Felis catus	Domestic cat	*I. (A.) brewsterae*	F	Liberia	27
				I. (A.) cavipalpus	A	Africa	9
				I. (A.) ceylonensis	A, N	India	42, 43
				I. (A.) cumulatimpunctatus	F	Ivory Coast	9, 17
				I. (A.) lewisi	UD	Africa	9, 28
				I. (A.) moreli	F	Ivory Coast	9, 17
				I. (A.) muniensis	A, N	Equatorial Guinea, Ivory Coast	9, 17, 19, 25
				I. (A.) oldi	F	Ghana, Ivory Coast, Liberia	9, 17, 27
				I. (A.) rubicundus	F, M, N, L	South Africa	16, 21
		Felis silvestris lybica	African wildcat	*I. (A.) procaviae*	F	South Africa	40
				I. (A.) rubicundus	F, M	South Africa	11, 21, 40, 41
		Felis sp.	Cat (wild or domestic)	*I. (A.) pilosus*	UD	South Africa	21
		Leptailurus serval	Serval	*I. (A.) oldi*	F	Sierra Leone	27, 35
				I. (A.) pilosus	UD	South Africa	44
		Panthera leo	Lion	*I. (A.) procaviae*	F, N	South Africa	11, 40
		Panthera pardus	Leopard	*I. (A.) cumulatimpunctatus*	F	Ghana, Ivory Coast	17, 19, 21
				I. (A.) moreli	F	Ivory Coast	17, 21

Order	Family	Host species (scientific name)	Host species (common name)	*Ixodes* (*Afrixodes*) species	Life stage	Locality	Reference
				I. (A.) muniensis	A	DRC, Cameroon, Ivory Coast	6, 17, 19, 25, 26
				I. (A.) neitzi	F, A	South Africa	11, 45
				I. (A.) oldi	F	Ivory Coast	17, 21
				I. (A.) rasus	F	Cameroon	19
		UD	Cat and leopard	*I. (A.) myotomys*	F	Africa	28
	Herpestidae	*Atilax paludinosus*	Marsh mongoose	*I. (A.) brewsterae*	A	Uganda	5, 27
				I. (A.) cumulatimpunctatus	L, N	Cameroon, Ivory Coast	17, 25, 26
				I. (A.) rasus	F, N	Cameroon, Gabon	22, 25
				I. (A.) vanidicus	A, N, L	Cameroon, Mozambique	25, 27, 28
		Atilax paludinosus robustus	Marsh mongoose	*I. (A.) brewsterae*	F	Uganda	5, 27
		Crossarchus obscurus	Common kusimanse (or Long-nosed kusimanse)	*I. (A.) cumulatimpunctatus*	F, N	Ivory Coast	17, 26
				I. (A.) muniensis	F, N, L	Ivory Coast	17, 26
				I. (A.) rasus	N	Ivory Coast	17
		Herpestidae ichneumon	Egyptian mongoose	*I. (A.) aulacodi*	N, A	South Africa	8, 9
				I. (A.) brewsterae	F	South Africa	5, 27
		Herpestidae pulverulentus	Cape grey mongoose	*I. (A.) corwini*	F, N, L	South Africa	8, 27
		Herpestidae sanguina	Angola (or Common) slender mongoose	*I. (A.) corwini*	F, N, L	South Africa	8, 27
				I. (A.) procaviae	N	South Africa	11, 40
		Herpestidae smithii zeylanicus	Ruddy mongoose	*I. (A.) ceylonensis*	F	Sri Lanka	46, 47
		Herpestidae sp.	Mongoose	*I. (A.) brewsterae*	F	South Africa, Uganda	5, 27
				I. (A.) ceylonensis	A, N	India	42, 43
				I. (A.) pilosus	UD	South Africa	21
				I. (A.) rasus	M	DRC	19
		Ichneumia albicauda	White-tailed mangoose	*I. (A.) rasus*	F, N	Cameroon	19
		Mungos melanurus	Banded mongoose	*I. (A.) cumulatimpunctatus*	F	Malawi	19

Order	Family	Host species (scientific name)	Host species (common name)	*Ixodes* (*Afrixodes*) species	Life stage	Locality	Reference
		UD	Mongoose	*I. (A.) radfordi*	UD	India	42, 43
				I. (A.) ugandanus	A	Africa	28
				I. (A.) walkerae	A	Africa	28
	Hyaenidae	*Crocuta crocuta*	Spotted hyena	*I. (A.) procaviae*	F, L	South Africa	11, 40
		Hyaena brunnea	Brown hyena	*I. (A.) neitzi*	UD	South Africa	11, 45
		Proteles cristatus	Aardwolf	*I. (A.) procaviae*	L	South Africa	18
	Mustelidae	*Aonyx capensis*	African clawless otter	*I. (A.) corwini*	A	South Africa	8, 18
	Nandiniidae	*Nandinia binotata*	African palm civet	*I. (A.) cumulatimpunctatus*	F	Cameroon	6
				I. (A.) moreli	F	Ivory Coast	17
				I. (A.) oldi	F	Ivory Coast	27
				I. (A.) rasus	A, N	Cameroon, Gabon, Ivory Coast	17, 19, 22
	Viverridae	*Civettictis civetta*	African civet	*I. (A.) ampullaceus*	F	South Africa, Zimbabwe	49
				I. (A.) aulacodi	A, N, L	DRC, Ghana	7, 9, 24
				I. (A.) cumulatimpunctatus	A, N, L	DRC, Ghana, Ivory Coast	7, 17, 23, 24
				I. (A.) moreli	A	Ghana, Ivory Coast	17, 24
				I. (A.) muniensis	A	Ghana, Ivory Coast	17, 23, 23
				I. (A.) oldi	A, N, L	Ghana, Ivory Coast, Sierra Leone	17, 24, 27, 48
				I. (A.) procaviae	F, N, L	South Africa	11, 40
				I. (A.) rasus	A, N, L	DRC, Ghana	19, 22, 23, 24
		Civettictis civetta schwarzi	Schwarzi's african civet	*I. (A.) aulacodi*	UD	DRC	6, 9
				I. (A.) cumulatimpunctatus	F, M	DRC	6
				I. (A.) nairobiensis	UD	Uganda	6, 9
		Civettictis scivetta congica	Civet	*I. (A.) cavipalpus*	A	Sudan	6, 50
		Civettictis sp.		*I. (A.) vanidicus*	A	Cameroon	5, 9, 25, 27
		Genetta genetta	Common genet	*I. (A.) ampullaceus*	F	Zimbabwe	49
				I. (A.) corwini	N, L	South Africa	8, 27

Order	Family	Host species (scientific name)	Host species (common name)	*Ixodes* (*Afrixodes*) species	Life stage	Locality	Reference
				I. (A.) macfarlanei	A	Uganda	27, 28
				I. (A.) oldi	F	Tanzania	27
				I. (A.) procaviae	N, L	South Africa	11, 18, 40
		Genetta genetta hintoni	Common genet	*I. (A.) cumulatimpunctatus*	F, M, N, L	DRC	6, 7
		Genetta genetta neumanni	Common genet	*I. (A.) oldi*	F	Kenya	27, 35
		Genetta maculata	Cape genet	*I. (A.) macfarlanei*	A	Uganda	27, 28
				I. (A.) oldi	F	Ivory Coast	17
		Genetta sp.	Genet	*I. (A.) oldi*	F, M, N	Ivory Coast, Kenya, Liberia, Tanzania	5, 9, 17, 27, 35
				I. (A.) procaviae	N	South Africa	11, 40
		Genetta tigrina	Cape genet (or Large-spotted genet)	*I. (A.) corwini*	F, N	South Africa	8, 27
				I. (A.) djaronensis	A	Tanzania	5, 28, 51
				I. (A.) macfarlanei	A	Uganda	27, 28
				I. (A.) procaviae	N	South Africa	11, 40
				I. (A.) rasus	F, M	DRC	19
		Paradoxurus hermaphroditus	Asian palm civet	*I. (A.) ceylonensis*	UD	India	36
		UD	Civet or genet	*I. (A.) ugandanus*	A, N	Africa	9, 28
				I. (A.) walkerae	A	Africa	28
	UD	UD	Carnivore	*I. (A.) arebiensis*	UD	Africa	9
**Hyracoidea**	Procaviidae	*Dendrohyrax arboreus*	Southern tree hyrax	*I. (A.) procaviae*	F	DRC, Rwanda	5, 9, 19, 51
				I. (A.) rasus	UD	Rwanda	6
		Dendrohyrax dorsalis	Western tree hyrax	*I. (A.) cumulatimpunctatus*	N	Ivory Coast	9, 17
				I. (A.) rasus	F	Ivory Coast	17
		Dendrohyrax sp.	Hyrax	*I. (A.) muniensis*	N	Ivory Coast	9, 17
		Heterohyrax brucei	Bush hyrax	*I. (A.) brumpti*	F	Ethiopia	5, 51
				I. (A.) lewisi	UD	Zambia	9, 28, 52
				I. (A.) matopi	N, L	Zimbabwe	9, 53
		Procavia capensis		*I. (A.) hyracis*	F	South Africa	76

Order	Family	Host species (scientific name)	Host species (common name)	*Ixodes* (*Afrixodes*) species	Life stage	Locality	Reference
			Rock hyrax (or dassie)	*I. (A.) matopi*	N, L	Zimbabwe	9 53
				I. (A.) procaviae	F, N	South Africa	8, 11, 12
				I. (A.) spinae	F, M	South Africa	5, 7, 9
		Procavia capensis johnstoni	Eastern rock hyrax	*I. (A.) procaviae*	F	DRC	6
		Procavia sp.	Hyrax or dassie	*I. (A.) cumulatimpunctatus*	F	Cameroon, DRC	6, 19
		UD	Hyraxes and dassies	*I. (A.) moreli*	UD	Africa	9
				I. (A.) rasus	F, M	DRC	9, 19
**Lagomorpha**	Leporidae	*Lepus saxatilis*	Scrub hare	*I. (A.) catherinei*	F	South Africa	5, 8, 9, 27
				I. (A.) cavipalpus	A	South Africa	9, 16
				I. (A.) pilosus	A, N, L	South Africa	8, 9, 18, 21
				I. (A.) rubicundus	N, L	South Africa	14, 21
		Lepus victoriae microtis	African savanna hare	*I. (A.) cavipalpus*	M	Sudan	50
		Pronolagus crassicaudatus	Natal red rock hare	*I. (A.) matopi*	N, L	Zimbabwe	9, 53
		Pronolagus rupestris	Smith’s red rock hare	*I. (A.) procaviae*	UD	South Africa	11, 12
				I. (A.) rubicundus	N, L	South Africa	8, 14, 21
**Macroscelidea**	Macroscelididae	*Elephantulus myurus*	Eastern rock elephant shrew	*I. (A.) rubicundus*	N, L	South Africa	8, 11, 30, 40
		Petrodromus tetradactylus	Four-toed sengi	*I. (A.) nchisiensis*	F, M	Malawi	5, 35
				I. (A.) oldi	F	Zambia	27
				I. (A.) rhabdomysae	UD	Zambia	52
		Rhynchocyon cirnei	Checquered elephant shrew / chequered sengi	*I. (A.) rasus*	UD	Zambia	52
		UD	Elephant shrew	*I. (A.) vanidicus*	A	Africa	28
**Perissodactyla**	Equidae	*Equus zebra zebra*	Plains zebra	*I. (A.) rubicundus*	A, L	South Africa	11, 13
		UD	Horse, zebra	*I. (A.) cumulatimpunctatus*	UD	Africa	9
	Rhinocerotidae	*Ceratotherium simum*	White rhinoceros	*I. (A.) rubicundus*	A	South Africa	10, 11
		UD	Rhinoceros	*I. (A.) cumulatimpunctatus*	UD	Africa	9
**Pholidota**	Manidae	*Phataginus tetradactyla*	Long-tailed pangolin	*I. (A.) cumulatimpunctatus*	N	Ivory Coast	9, 17

Order	Family	Host species (scientific name)	Host species (common name)	*Ixodes* (*Afrixodes*) species	Life stage	Locality	Reference
				I. (A.) oldi	N	Ivory Coast	9, 17
		Phataginus tricuspis	Tree pangolin	*I. (A.) cumulatimpunctatus*	A	Gabon	22
				I. (A.) rasus	A, N	Cameroon, Equatorial Guinea, Gabon, Uganda	9, 19, 22, 25
**Primates**	Cercopithecidae	*Cercocebus albigena*	White-eyelid mangabey	*I. (A.) rasus*	F	DRC	9, 19, 54
		Cercopithecus ascanius	Red-tailed monkey	*I. (A.) rasus*	F	DRC	6, 9
		Cercopithecus cephus	Moustached guenon	*I. (A.) rageaui*	F, M	Cameroon, Gabon	22, 25, 32, 54
		Cercopithecus diana	Diana monkey	*I. (A.) cumulatimpunctatus*	L	Ivory Coast	17, 26
		Cercopithecus mitis	Blue monkey	*I. (A.) rageaui*	A	Rwanda	5, 6, 54
		Colobus caudatus	Mount Kilimanjaro guereza	*I. (A.) schillingsi*	N	Burundi, Rwanda, Tanzania	28, 32
		Colobus polykomos caudatus	King colobus	*I. (A.) schillingsi*	M, N	Tanzania	28, 54
		Colobus polykomos dodingae	King colobus	*I. (A.) schillingsi*	F	Sudan	28, 50, 54
		Colobus polykomos matschei	King colobus	*I. (A.) schillingsi*	F	Kenya	28, 54
		Colobus sp.	Colobus (or colobi)	*I. (A.) schillingsi*	A	Kenya, Sudan, Tanzania	5, 28, 32, 54
		Papio cynocephalus	Yellow baboon	*I. (A.) cavipalpus*	F	Zambia	54
		Piliocolobus badius	Western red colobus	*I. (A.) rasus*	F	Ivory Coast	9, 19, 54
		UD	Baboon	*I. (A.) ceylonensis*	A, N	India	28
	Cheirogaleidae	*Cheirogaleus major*	Greater dwarf lemur	*I. (A.) lemuris*	UD	Madagascar	2
		Microcebus sp	Mouse lemur	*I. (A.) lemuris*	UD	Madagascar	55
	Galagidae	*Otolemur crassicaudatus*	Brown greater galago / Thick-tailed greater galago	*I. (A.) rhabdomysae*	F	South Africa	61
				I. (A.) schillingsi	N, L	Zanzibar	28, 54
		UD	Bushbabies	*I. (A.) cavipalpus*	A	Africa	9
				I. (A.) pilosus	UD	Africa	9
	Hominoidae	*Homo sapiens*	Man	*I. (A.) cumulatimpunctatus*	UD, F	Ivory Coast, Rwanda, Zimbabwe	6, 9, 17, 19
				I. (A.) muniensis	M	Ivory Coast	17, 56

Order	Family	Host species (scientific name)	Host species (common name)	*Ixodes* (*Afrixodes*) species	Life stage	Locality	Reference
				I. (A.) rageaui	F	Cameroon	5, 9, 21, 25
				I. (A.) rasus	F	Ivory Coast	9, 17
				I. (A.) schillingsi	F	Kenya	28, 54
				I. (A.) vanidicus	UD	Africa	28
		UD	Monkeys, Man	*I. (A.) cavipalpus*	A	Africa	9
				I. (A.) pilosus	UD	Africa	9
	Indriidae	*UD*	Indris	*I. (A.) lemuris*	UD	Madagascar	5
	Lemuridae	*Eulemur rufifrons*	Red-fronted lemur	*I. (A.) lemuris*	F	Madagascar	2, 32
	Lorisidae	*UD*	Lorisid	*I. (A.) ugandanus*	A	Africa	9, 28
**Proboscidea**	Elephantidae	*UD*	Elephant	*I. (A.) elongatus*	F	South Africa	8
**Rodentia**	Cricetidae	*Lophiomys imhausi*	Crested rat (or maned rat)	*I. (A.) schillingsi*	F	Kenya	28, 54
	Gliridae	*Graphiurus microtis*	Small-eared dormouse	*I. (A.) muniensis*	N	Kenya	
	Hystricidae	*Atherurus africanus*	African bush-tailed porcupine	*I. (A.) aulacodi*	F	Ivory Coast	17
				I. (A.) cumulatimpunctatus	A, N	Gabon, Ivory Coast	17, 22
				I. (A.) rasus	A, N, L	DRC, Gabon, Ghana	6, 22, 23, 24
		Hystrix indica	Indian crested porcupine	*I. (A.) ceylonensis*	UD	India	43, 57
	Muridae	*Aethomys chrysophilus*	Red rock rat	*I. (A.) rhabdomysae*	UD	Zambia	9, 52
		Aethomys kaiseri walambae	Kaiser's rock rat	*I. (A.) rhabdomysae*	UD	Zambia	9, 52
		Arvicanthis abyssinicus	Abyssinian grasse rat	*I. (A.) thomasae*	F	Kenya	5, 8, 19
		Arvicanthis niloticus	African grass rat	*I. (A.) nairobiensis*	F	DRC	5, 6, 7, 9
				I. (A.) nchisiensis	UD	DRC	6, 9, 27, 59
		Bandicota sp.	Bandicoot	*I. (A.) ceylonensis*	UD	India	43, 57
				I. (A.) heinrichi	UD	Africa	5
		Dasymys sp.	African marsh rat (or Common dasymys, Water rat)	*I. (A.) cumulatimpunctatus*	UD	RC	26
				I. (A.) rasus	F	DRC	6, 9
		Grammomys dolichurus	Woodland thicket rat	*I. (A.) rhabdomysae*	UD	Zambia	9, 52

Order	Family	Host species (scientific name)	Host species (common name)	*Ixodes* (*Afrixodes*) species	Life stage	Locality	Reference
		Golunda ellioti	Indian bush rat	*I. (A.) ceylonensis*	UD	India	43, 57
		Lemniscomys striatus	Striped grass mouse	*I. (A.) cumulatimpunctatus*	UD	DRC	26
		Lophuromys aquilus	Grey brush-furred rat	*I. (A.) rasus*	F	Malawi	9, 19
		Lophuromys sikapusi	Rusty-bellied brush-furred rat	*I. (A.) cumulatimpunctatus*	UD	RC	26
		Lophuromys sp.	Brush-furred mouse	*I. (A.) browningi*	F	DRC	6, 9
		Madromys blanfordi	Blanford's rat	*I. (A.) ceylonensis*	UD	India	43, 57
		Mastomys coucha ugandanus	Southern multimammate mouse	*I. (A.) auriculaelongae*	F	DRC	6
		Mastomys natalensis	Natal multimammate mouse	*I. (A.) auriculaelongae*	UD	Zambia	9, 52
				I. (A.) calcarhebes	F	Zambia	60
				I. (A.) rhabdomysae	UD	Zambia	9, 52
				I. (A.) thomasae	UD	Zambia	5, 9, 52
		Mastomys natalensis ismailiae	Natal multimammate mouse	*I. (A.) nairobiensis*	N	Sudan	5, 6, 9, 50
		Mastomys sp.	Mouse	*I. (A.) nairobiensis*	UD	South Africa	5, 6, 9, 50
				I. (A.) thomasae	F	DRC	6
		Micaelamys namaquensis	Namaqua rock rat	*I. (A.) nairobiensis*	UD	South Africa	5, 6, 9
				I. (A.) rubicundus	N, L	South Africa	5, 9, 14
				I. (A.) transvaalensis	F	South Africa	5, 8
		Montemys delectorum	Delectable soft-furred mouse	*I. (A.) auriculaelongae*	UD	Zambia	9, 52
				I. (A.) cumulatimpunctatus	F	Kenya	58
		Mus musculoides	Temminck's mouse	*I. (A.) oldi*	A	DRC	6, 9
		Mus musculus	House mouse	*I. (A.) ambohitantelensis*	F	Madagascar	3
		Mus sp.	Mouse	*I. (A.) auriculaelongae*	F	Tanzania	9, 35
				I. (A.) ceylonensis	UD	India	43, 57
		Mus triton	Gray-bellied pygmy mouse	*I. (A.) rhabdomysae*	UD	Zambia	9, 52
		Mus (Leggada) sp.	Pigmy mice	*I. (A.) cumulatimpunctatus*	L	Cameroon	9, 25, 26
		Otomys irroratus	Southern african vlei rat	*I. (A.) bakeri*	F, N	South Africa	62, 63
				I. (A.) elongatus	F	South Africa	8, 9, 62

Order	Family	Host species (scientific name)	Host species (common name)	*Ixodes* (*Afrixodes*) species	Life stage	Locality	Reference
				I. (A.) procaviae	UD	South Africa	11, 62
				I. (A.) rubicundus	L	South Africa	5, 9, 33, 64
		Otomys unisulcatus	Bush vlei rat	*I. (A.) myotomys*	F	South Africa	5, 8, 9
				I. (A.) rubicundus	L	South Africa	5, 9, 14
		Otomys sp.	Dent's vlei rat	*I. (A.) bakeri*	A, N	South Africa	8
				I. (A.) bedfordi	UD	Lesotho	8, 9
		Otomys tropicalis elgonis	Tropical vlei rat	*I. (A.) thomasae*	F	Kenya	5, 9, 19
		Pelomys fallax	Creek groove-toothed swamp rat	*I. (A.) rhabdomysae*	UD	Zambia	9, 52
				I. (A.) thomasae	UD	Zambia	5, 9, 52
		Pelomys fallax concolor	Creek groove-toothed swamp rat	*I. (A.) nairobiensis*	UD	Uganda	5, 6, 9
		Praomys sp.	African soft-furred rat	*I. (A.) bakeri*	N	South Africa	63
				I. (A.) browningi	F	DRC	6, 9
		Rattus rattus	Black rat	*I. albignaci*	F	Madagascar	2
				I. ceylonensis	A	Sri Lanka	65
				I. colasbelcouri	F, M, N, L	Madagascar	1, 2, 75
				I. lemuris	F	Madagascar	2
				I. radfordi	A	India	28, 66
				I. randrianasoloi	F	Madagascar	1, 2, 5, 28
		Rattus rattus kandianus	Black rat	*I. ceylonensis*	F	Sri Lanka	46, 47
		Rattus rattus rufescens	White-bellied wood rat	*I. (A.) ceylonensis*	UD	India	43, 57
		Rattus rattus wroughtoni	White-bellied wood rat	*I. (A.) ceylonensis*	UD	India	43, 57
		Rhabdomys pumilio	Four-striped grass mouse	*I. (A.) bakeri*	A	South Africa	62, 64
				I. (A.) fynbosensis	F, N, L	South Africa	38, 62
				I. (A.) procaviae	UD	South Africa	11, 62
				I. (A.) rhabdomysae	F	South Africa	8, 9
				I. (A.) rubicundus	UD	South Africa	5, 9, 14

Order	Family	Host species (scientific name)	Host species (common name)	*Ixodes* (*Afrixodes*) species	Life stage	Locality	Reference
		Rhabdomys spp.	Four-striped grass mouse	*I. (A.) bedfordi*	A	Lesotho	8, 9
		Tatera sp.	Gerbil	*I. (A.) aulacodi*	UD	Cameroon	25, 26
				I. (A.) cumulatimpunctatus	L	Cameroon	25, 26
		Thallomys sp.	Acacia rat	*I. (A.) ugandanus*	F	Ethiopia	49
		Uranomys ruddi	Rudd's mouse	*I. (A.) aulacodi*	UD	Ivory Coast	26
				I. (A.) muniensis	UD	Ivory Coast	9, 26
		UD	Muriid	*I. (A.) loveridgei*	UD	Africa	5, 9
				I. (A.) nesomys	UD	Madagascar	1, 5
		Zelotomys hildegardeae	Hildegarde's broad-headed mouse	*I. (A.) rhabdomysae*	UD	Zambia	9, 52
	Nesomyidae	*Brachyuromys betsileoensis*	Lesser short-tailed rat	*I. (A.) colasbelcouri*	F	Madagascar	2
		Cricetomys gambianus	Gambian pouched rat	*I. (A.) cumulatimpunctatus*	F, M	DRC, Equatorial Guinea (island), Ivory Coast, RC, Zambia, Zimbabwe	6, 7, 17, 19, 25, 26, 52
				I. (A.) oldi	F	Ivory Coast	17
				I. (A.) rasus	F	Ivory Coast	17
		Cricetomys gambianus viator	Gambian pouched rat	*I. (A.) loveridgei*	A	Malawi	35
		Cricetomys sp.	Giant pouched rat	*I. (A.) ampullaceus*	F	Uganda	49
				I. (A.) aulacodi	A, N, L	Ghana	24
				I. (A.) cumulatimpunctatus	A, N, L	Ghana, Malawi, Uganda	19, 23, 24
				I. (A.) loveridgei	A, N, L	Ghana	23
				I. (A.) muniensis	F, M, N, L	Ivory Coast, Rwanda	6, 17
		Nesomys audeberti	White-bellied nesomys	*I. (A.) lunatus*	F	Madagascar	2, 75
		Nesomys rufus	Island mouse (or Eastern red forest rat)	*I. (A.) colasbelcouri*	F	Madagascar	2
				I. (A.) nesomys	F	Madagascar	1, 2, 67
		Saccostomus campestris		*I. (A.) rhabdomysae*	UD	Zambia	9, 52

Order	Family	Host species (scientific name)	Host species (common name)	*Ixodes* (*Afrixodes*) species	Life stage	Locality	Reference
			South african pouched mouse	*I. (A.) rubicundus*	L	South Africa	14
	Sciuridae	*Funisciurus anerythrus*	Thomas's rope squirrel	*I. (A.) browningi*	F	DRC	6
		Funisciurus pyrropus leucostigma	Fire-footed rope squirrel	*I. (A.) cumulatimpunctatus*	L	Ivory Coast	17, 26, 28
		Funambulus tristriatus	Jungle palm squirrel	*I. (A.) ceylonensis*	UD	India	43, 57
		Heliosciurus gambianus	Gambian sun squirrel	*I. (A.) muniensis*	L	Ivory Coast	17, 26
		Heliosciurus rufobrachium rubicatus	Ruwenzori sun squirrel	*I. (A.) browningi*	F	DRC	6
		Heliosciurus ruwenzorii	Red-legged sun squirrel	*I. (A.) browningi*	F	Rwanda	6
		Paraxerus lucifer	Gambian sun squirrel	*I. (A.) latus*	F	Malawi	28, 35
		Paraxerus sp.	African bush squirrel	*I. (A.) oldi*	N	Kenya	35
		UD	Squirrel	*I. (A.) browningi*	F, M	DRC	6
				I. (A.) latus	UD	Africa	5
	Spalacidae	*Tachyoryctes ruandae*	Rwanda african mole-rat	*I. (A.) rotundatus*	F, N, L	DRC	6
		Tachyoryctes sp.	Mole-rat	*I. (A.) oldi*	N	Kenya	35
				I. (A.) rotundatus	F	Kenya	9, 21, 35
	Thryonomyidae	*Thryonomys gregorianus*	Lesser cane rat	*I. (A.) ampullaceus*	F	Malawi	49
		Thryonomys swinderianus	Greater cane rat / grasscutter	*I. (A.) ampullaceus*	F	Mozambique, Tanzania, South Africa, Zimbabwe	49
				I. (A.) aulacodi	A, N, L	Benin, Cameroon, DRC, Gabon, Ghana, Ivory Coast, South Africa	5, 6, 8, 9, 17, 22, 23, 24, 25, 26, 68, 69
				I. (A.) cumulatimpunctatus	A, N, L	Ghana	23, 24
				I. (A.) moreli	A	Ghana	24
				I. (A.) muniensis	A	Ghana	23, 24
				I. (A.) rasus	A, N, L	DRC, Ghana	19, 23, 24
				I. (A.) ugandanus	F	South Africa	5, 8, 9, 28, 31, 49

Order	Family	Host species (scientific name)	Host species (common name)	*Ixodes* (*Afrixodes*) species	Life stage	Locality	Reference
		Thryonomys sp.	Cane rat	*I. (A.) ampullaceus*	F	Kenya, South Africa	49
				I. (A.) ugandanus	F	Uganda	49
	UD	*UD*	Rats, mice, porcupine	*I. (A.) minutae*	UD	Africa	5
				I. (A.) pilosus	N, L	Africa	9, 18
				I. (A.) spinae	F	DRC	7, 9
**Soricomorpha**	Soricidae	*Crocidura flavescens*	Greater red musk shrew	*I. (A.) nchisiensis*	UD	Center Africa	9, 27
				I. (A.) zairensis	F, N	DRC	5, 27
		Crocidura flavescens occidentalis	Greater red musk shrew	*I. (A.) nchisiensis*	UD	Ivory Coast, RC	26
				I. (A.) oldi	F, M	DRC	6
		Crocidura hirta	Lesser red musk shrew	*I. (A.) rhabdomysae*	UD	Zambia	52
		Crocidura mariquensis	Swamp musk shrew	*I. (A.) bakeri*	UD	South Africa	8
		Crocidura miya	Sri Lankan long-tailed shrew	*I. (A.) ceylonensis*	L	Sri Lanka	47
		Crocidura munissii	Munissi's shrew	*I. (A.) zairensis*	UD	DRC	5, 27
		Crocidura occidentalis	Shrew	*I. (A.) aulacodi*	UD	Ivory Coast	26
				I. (A.) auriculaelongae	UD	Zambia	9, 52
				I. (A.) nchisiensis	UD	CAR	26
		Crocidura sp.	Shrew	*I. (A.) auriculaelongae*	F	DRC	6, 9
				I. (A.) ceylonensis	N, L	India	28
				I. (A.) nairobiensis	N, F	DRC	6, 9
				I. (A.) oldi	F, M, N, L	DRC	6, 9
				I. (A.) zairensis	F	DRC	5, 27
		Myosorex varius	Forest shrew	*I. (A.) bakeri*	UD	South Africa	8
		Solisorex pearsoni	Pearson's long-clawed shrew	*I. (A.) ceylonensis*	L	Sri Lanka	47
		Suncus montanus	Asian higland shrew	*I. (A.) ceylonensis*	N, L	Sri Lanka	47
		Suncus murinus	Asian hous shrew	*I. (A.) ceylonensis*	UD	India	43, 57
		UD	Shrew	*I. (A.) cavipalpus*	A	Africa	9

Order	Family	Host species (scientific name)	Host species (common name)	*Ixodes* (*Afrixodes*) species	Life stage	Locality	Reference
				I. (A.) cumulatimpunctatus	UD	Africa	9
				I. (A.) elongatus	F	Africa	8, 9
				I. (A.) fynbosensis	N, L	South Africa	38
**Reptilia**							
**Testudines**	Testudinoidea	*Kinixys erosa*	Forest hinge-back tortoise	*I. (A.) rasus*	A	Gabon	22
**Aves**							
**Cuculiformes**	Cuculidae	*Centropus senegalensis*	Senegal coucal	*I. (A.) cumulatimpunctatus*	M	Ivory Coast	9, 17
		Centropus superciliosus	White-browed coucal	*I. (A.) cumulatimpunctatus*	F	Uganda	19
		UD	Cuckoo	*I. (A.) thomasae*	A	UD	28
**Galliformes**	Numididae	*Agelastes niger*	Black guineafowl	*I. (A.) cumulatimpunctatus*	L	Cameroon	25, 26
				I. (A.) rasus	N	Cameroon	25
		Guttera plumifera	Plumed guineafowl	*I. (A.) cumulatimpunctatus*	L	Cameroon	25, 26
				I. (A.) rasus	N	Cameroon	25
		Numida meleagris	Helmeted guineafowl	*I. (A.) ampullaceus*	F	South Africa	49
				I. (A.) aulacodi	UD, A, N	Cameroon, South Africa	8, 25, 26
				I. (A.) rasus	N	Cameroon	25
		UD	Guineafowl	*I. (A.) walkerae*	A	UD	28
	Phasianidae	*Gallus gallus*	Domestic chicken	*I. (A.) cumulatimpunctatus*	N	Ivory Coast	9, 17
		Pternistis bicalcaratus	Double-spurred spurfowl	*I. (A.) rasus*	N	Cameroon	25
	UD	UD	Gallinaceous	*I. (A.) arebiensis*	UD	UD	9
				I. (A.) aulacodi	UD	UD	9
				I. (A.) cumulatimpunctatus	UD	UD	9
				I. (A.) rasus	UD	UD	9
**Passeriformes**	Alaudidae	UD	Alaudidae	*I. (A.) ceylonensis*	N, L	India	28
	Cisticolidae	*Cisticola chiniana*	Rattling cisticola	*I. (A.) euplecti*	F	Zambia	71
		Cisticola natalensis	Croaking cisticola	*I. (A.) euplecti*	F	Ethiopia	71

Order	Family	Host species (scientific name)	Host species (common name)	*Ixodes* (*Afrixodes*) species	Life stage	Locality	Reference
		Cisticola tinniens	Tiny cisticola	*I. (A.) procaviae*	N	South Africa	70
		Schistolais leucopogon	White-chinned prinia	*I. (A.) aulacodi*	N	Cameroon	25, 68
	Fringillidae	*Crithagra mozambica*	Yellow-fronted canary	*I. (A.) euplecti*	F	Ethiopia	71
	Macrosphenidae	*Melocichla mentalis*	Moustached grass warbler	*I. (A.) heinrichi*	F	Angola	72
	Malaconotidae	*Tchagra senegalus*	Bushshrike	*I. (A.) euplecti*	F	Zambia	52, 71
		UD	Bushshrike	*I. (A.) thomasae*	A	UD	9, 28
	Motacillidae	*Anthus trivialis*	Tree pipit	*I. (A.) cumulatimpunctatus*	N	Malta	73
				I. (A.) euplecti	F	Egypt	71
				undescribed *Afrixodes*	F	DRC	7, 76
	Muscicapidae	*Oenanthe oenanthe*	Northern wheatear	*I. (A.) euplecti*	F	Egypt	71
	Ploceidae	*Euplectes afer*	Yellow-crowned bishop	*I. (A.) euplecti*	F	Zambia	71
				I. (A.) procaviae	N	South Africa	70
		Euplectes capensis	Yellow bishop	*I. (A.) euplecti*	F	Zambia	71
		Euplectes gierowii	Black bishop	*I. (A.) euplecti*	F	Ethiopia	71
		Euplectes hordeaceus	Black-winged red bishop	*I. (A.) euplecti*	F	Ethiopia	71
		Euplectes macroura	Yellow-mantled widowbird	*I. (A.) euplecti*	A	RC	35, 71
		Euplectes orix	Southern red bishop	undescribed *Afrixodes*	F	South Africa	70, 76
		Ploceus baglafecht	Baglafecht weaver	*I. (A.) euplecti*	F	Ethiopia	71
		UD	Bishop	*I. (A.) thomasae*	A	UD	9, 28
	Pycnonotidae	*Atimastillas flavicollis*	Yellow-throated leaflove	*I. (A.) euplecti*	F	Ethiopia	71
	Sylviidae	UD	Sylviid	*I. (A.) heinrichi*	UD	UD	28
	Turdidae	*Geokichla piaggiae*	Abyssinian ground thrush	*I. (A.) euplecti*	F	Ethiopia	71
		Stizorhina fraseri	Finsch's flycatcher-thrush	*I. (A.) cumulatimpunctatus*	L	Cameroon	25, 26
				I. (A.) rasus	L	Cameroon	25
		Turdus olivaceus	Olive thrush	*I. (A.) walkerae*	F	Kenya	74

Order	Family	Host species (scientific name)	Host species (common name)	*Ixodes* (*Afrixodes*) species	Life stage	Locality	Reference
		Turdus pelios	African thrush	*I. (A.) euplecti*	F, N	Ethiopia	71
		UD	Thrushes	*I. (A.) walkerae*	A	UD	28
	UD	UD	Passerine	*I. (A.) arebiensis*	UD	UD	9
				I. (A.) aulacodi	UD	UD	9
				I. (A.) cumulatimpunctatus	UD	UD	9
				undescribed *Afrixodes*	UD	UD	9, 76

Abbreviations: A, adults, female or male; F, female; M, male; N, nymph; L, larva; UD, undetermined; CAR, Central Africa Republic; DRC, Democratic Republic of Congo; RC, Republic of Congo.

Supplementary Table S4. Tick-host associations reported in the publications included in the review.

Species of *Ixodes* (*Afrixodes*)	Host order	Host family	Host species	Reference
I. (*A.*) *albignaci*	Afrosoricida	Tenrecidae	*Nesogale talazaci*	Uilenberg et al. (1979); Kolonin (2007); Apanaskevich and Goodman (2020)
	Rodentia	Muridae	*Rattus rattus*	Uilenberg et al. (1979)
I. (*A.*) *ambohitantelensis*	Afrosoricida	Tenrecidae	*Microgale majori*	Englert et al. (2023)
	Rodentia	Muridae	*Mus musculus*	Englert et al. (2023)
I. (*A.*) *ampullaceus*	Carnivora	Viverridae	*Civettictis civetta*	Apasnakevich et al. (2025)
			Genetta tigrina	Apasnakevich et al. (2025)
	Galliformes	Numididae	*Numida meleagris*	Apasnakevich et al. (2025)
	Rodentia	Nesomyidae	*Cricetomys* sp.	Apasnakevich et al. (2025)
		Thryonomyidae	*Thryonomys gregorianus*	Apasnakevich et al. (2025)
			Thryonomys sp.	Apasnakevich et al. (2025)
			Thryonomys swinderianus	Apasnakevich et al. (2025)
I. (*A.*) *arebiensis*	Carnivora	UD	UD	Cumming (1998)
	Galliformes	UD	UD	Cumming (1998)
	Passeriformes	UD	UD	Cumming (1998)
I. (*A.*) *aulacodi*	Artiodactyla	Bovidae	*Cephalophus niger*	Ntiamoa-Baidu et al. (2004); Ntiamoa-Baidu et al. (2005)
			Neotragus pygmaeus	Ntiamoa-Baidu et al. (2004); Ntiamoa-Baidu et al. (2005)
			Philantomba maxwellii	Ntiamoa-Baidu et al. (2004); Ntiamoa-Baidu et al. (2005)
			Tragelaphus scriptus	Ntiamoa-Baidu et al. (2004); Ntiamoa-Baidu et al. (2005)
	Carnivora	Herpestidae	*Herpestidae ichneumon*	Walker (1991); Cumming (1998)
		Viverridae	*Civettictis civetta*	Clifford and Anastos (1962); Cumming (1998); Ntiamoa-Baidu et al. (2005)
			Civettictis civetta schwarzi	Elbl and Anastos (1966); Cumming (1998)

Species of *Ixodes* (*Afrixodes*)	Host order	Host family	Host species	Reference
	Galliformes	Numididae	*Numida meleagris galeatus*	Morel and Mouchet (1965); Morel (1966); Walker (1991); Cumming (1998)
	Passeriformes	Cisticolidae	*Schistolais leucopogon*	Morel and Mouchet (1965); Cumming (1998); Chitimia-Dobler et al. (2016)
	Rodentia	Hystricidae	*Atherurus africanus*	Aeschliman (1967)
		Muridae	*Tatera* sp.	Morel and Mouchet (1965); Morel (1966)
			Uranomys ruddi	Morel (1966)
		Nesomyidae	*Cricetomys* sp.	Ntiamoa-Baidu et al. (2004); Ntiamoa-Baidu et al. (2005)
		Thryonomyidae	*Thryonomys swinderianus*	Morel and Mouchet (1965); Aeschliman (1967); Elbl and Anastos (1966); Morel (1966); Walker (1991); Cumming (1998); Ntiamoa-Baidu et al. (2004); Ntiamoa-Baidu et al. (2005); Kolonin (2007); Pourrut et al. (2011); Chitimia-Dobler et al. (2016); Yessinou et al. (2022)
	Soricomorpha	Soricidae	*Crocidura olivieri occidentalis*	Morel (1966)
I. (*A.*) *auriculaelongae*	Rodentia	Muridae	*Mastomys coucha ugandanus*	Elbl and Anastos (1966); Cumming (1998)
			Montemys delectorum	Colbo and MacLeod (1976); Cumming (1998)
			Mastomys natalensis	Colbo and MacLeod (1976); Cumming (1998)
			Mus sp.	Arthur (1956); Cumming (1998)
	Soricomorpha	Soricidae	*Crocidura occidentalis*	Colbo and MacLeod (1976); Cumming (1998)
			Crocidura sp.	Elbl and Anastos (1966); Cumming (1998)
I. (*A.*) *bakeri*	Rodentia	Muridae	*Otomys irroratus*	Petney et al. (2004); Matthee et al. (2010)
			Otomys sp.	Walker (1991)
			Praomys sp.	Petney et al. (2004)
			Rhabdomys pumilio	Matthee et al. (2007); Matthee et al. (2010)
	Soricomorpha	Soricidae	*Crocidura mariquensis*	Walker (1991)
			Myosorex varius	Walker (1991)
I. (*A.*) *bedfordi*	Rodentia	Muridae	*Otomys* sp.	Walker (1991); Cumming (1998)

Species of *Ixodes* (*Afrixodes*)	Host order	Host family	Host species	Reference
			Rhabdomys sp.	Walker (1991); Cumming (1998)
I. (*A.*) *brewsterae*	Carnivora	Felidae	*Felis catus*	Keirans et al. (1982)
		Herpestidae	*Atilax paludinosus*	Keirans et al. (1982); Kolonin (2007)
			Atilax paludinosus robustus	Keirans et al. (1982); Kolonin (2007)
			Herpestidae ichneumon	Keirans et al. (1982); Kolonin (2007)
			Herpestidae sp.	Keirans et al. (1982); Kolonin (2007)
I. (*A.*) *browningi*	Rodentia	Muridae	*Lophuromys* sp.	Elbl and Anastos (1966); Cumming (1998)
			Praomys sp.	Elbl and Anastos (1966); Cumming (1998)
		Sciuridae	*Funisciurus anerythrus*	Elbl and Anastos (1966)
			Heliosciurus rufobrachium rubicatus	Elbl and Anastos (1966)
			Heliosciurus ruwenzorii	Elbl and Anastos (1966)
			UD	Elbl and Anastos (1966); Kolonin (2007)
I. (*A.*) *brumpti*	Hyracoidea	Procaviidae	*Heterohyrax brucei*	Morel (1965); Kolonin (2007)
I. (*A.*) *calcarhebes*	Rodentia	Muridae	*Mastomys natalensis*	Arthur (1980)
I. (*A.*) *catherienei*	Lagomorpha	Leporidae	*Lepus saxatilis*	Keirans et al. (1982); Walker (1991); Cumming (1998); Kolonin (2007)
I. (*A.*) *cavipalpus*	Artiodactyla	Bovidae	*Alcelaphus lichtensteinii*	Elbl and Anastos (1966)
			Bos taurus	Clifford et al. (1975); Cumming (1998)
			Capra hircus	Elbl and Anastos (1966); Cumming (1998)
			Cephalophus sp.	; Elbl and Anastos (1966)
			Oreotragus oreotragus	Clifford and Anastos (1962); Elbl and Anastos (1966)
			Ovis aries	Elbl and Anastos (1966); Cumming (1998) ;
			UD Antilopinae	Elbl and Anastos (1966)
		Giraffidae	UD	Cumming (1998)
	Carnivora	Canidae	*Canis familiaris*	Cumming (1998)
		Felidae	*Felis catus*	Cumming (1998)

Species of *Ixodes* (*Afrixodes*)	Host order	Host family	Host species	Reference
		Viverridae	*Civettictis civetta congica*	Hoogstraal (1956); Elbl and Anastos (1966)
	Lagomorpha	Leporidae	*Lepus saxatilis*	Clifford et al. (1975); Cumming (1998)
	Primates	Cercopithecidae	*Papio cynocephalus*	Hoogstraal and Theiler (1959)
		Galagidae	UD	Cumming (1998)
		Hominoidea	UD	Cumming (1998)
	Soricomorpha	Soricidae	UD	Cumming (1998)
I. (*A.*) *ceylonensis*	Artiodactyla	Bovidae	UD	Kolonin (2007), Guglielmone et al. (2020)
		Giraffidae	UD	Shobana and Gunasekaran (2018)
	Carnivora	Felidae	*Felis catus*	Bhat and Srinivasan (1981); Kolonin (2007); Muraleedharan (2017); Guglielmone et al. (2020)
		Herpestidae	*Herpestidae smithii zeylanicus*	Kohls (1947); Yamathramullage et al. (2014)
			Herpestidae sp.	Bhat and Srinivasan (1981); Kolonin (2007); Muraleedharan (2017); Guglielmone et al. (2020)
		Viverridae	*Paradoxurus hermaphroditus*	Shobana and Gunasekaran (2018)
	Passeriformes	Alaudidae	UD	Guglielmone et al. (2020)
	Primates	Cercopithecidae	UD	Guglielmone et al. (2020)
	Rodentia	Hystricidae	*Hystrix indica*	Rajagopalan et al. (1968); Muraleedharan (2017)
		Muridae	*Bandicota* sp.	Rajagopalan et al. (1968); Muraleedharan (2017); Guglielmone et al. (2020)
			Golunda ellioti	Rajagopalan et al. (1968); Muraleedharan (2017)
			Madromys blanfordi	Rajagopalan et al. (1968); Muraleedharan (2017)
			Mus sp.	Rajagopalan et al. (1968); Muraleedharan (2017)
			Rattus rattus	Yamathramullage et al. (2018)
			Rattus rattus kandianus	Kohls (1947); Yamathramullage et al. (2014)
			Rattus rattus rufescens	Rajagopalan et al. (1968); Muraleedharan (2017)
			Rattus rattus wroughtoni	Rajagopalan et al. (1968); Muraleedharan (2017)

Species of *Ixodes* (*Afrixodes*)	Host order	Host family	Host species	Reference
		Sciuridae	*Funambulus tristriatus*	Rajagopalan et al. (1968); Muraleedharan (2017); Guglielmone et al. (2020)
	Soricomorpha	Soricidae	*Crocidura miya*	Yathramullage et al. (2014)
			Crocidura sp.	Guglielmone et al. (2020)
			Solisorex pearsoni	Yathramullage et al. (2014)
			Suncus montanus	Yathramullage et al. (2014)
			Suncus murinus	Rajagopalan et al. (1968); Muraleedharan (2017)
I. (*A.*) *colasbelcouri*	Afrosoricida	Tenrecidae	*Nesogale talazaci*	Uilenberg et al. (1979)
			Tenrec ecaudatus	Uilenberg et al. (1979); Apanaskevich and Goodman (2020)
	Rodentia	Muridae	*Rattus rattus*	Uilenberg et al. (1979); Kolonin (2007); Apanaskevich and Goodman (2020)
		Nesomyidae	*Brachyuromys betsileoensis*	Uilenberg et al. (1979)
			Nesomys rufus	Uilenberg et al. (1979)
I. (*A.*) *corwini*	Carnivora	Canidae	*Canis familiaris*	Keirans et al. (1982); Horak et al. (1987); Kolonin (2007)
		Herpestidae	*Herpestidae pulverulentus*	Keirans et al. (1982); Walker (1991); Kolonin (2007)
			Herpestidae sanguineus	Keirans et al. (1982); Walker (1991)
		Mustelidae	*Aonyx capensis*	Keirans et al. (1982); Horak et al. (1987); Walker (1991); Kolonin (2007)
		Viverridae	*Genetta genetta*	Keirans et al. (1982); Walker (1991); Kolonin (2007)
			Genetta tigrina	Keirans et al. (1982); Walker (1991)
I. (*A.*) *cumulatimpunctatus*	Artiodactyla	Bovidae	*Bos taurus*	Arthur and Burrow (1957); Aeschliman (1967); Cumming (1998)
			Capra hircus	Arthur and Burrow (1957); Cumming (1998)
			Cephalophus dorsalis	Ntiamoa-Baidu et al. (2004); Pourrut et al. (2011)
			Cephalophus niger	Aeschliman (1967)Aeschliman (1967); Ntiamoa-Baidu et al. (2005); Morel (1966)
			Cephalophus nigrifrons nigrifrons	Elbl and Anastos (1966)

Species of *Ixodes* (*Afrixodes*)	Host order	Host family	Host species	Reference
			Cephalophus sp.	Arthur and Burrow (1957); Morel and Mouchet (1965); Cumming (1998)
			Cephalophus zebra	Aeschliman (1967)
			Neotragus moschatus akeleyi	Arthur and Burrow (1957)
			Neotragus pygmaeus	Aeschliman (1967); Morel (1966); Cumming (1998)
			Ourebia ourebi	Aeschliman (1967)
			Ovis aries	Cumming (1998)
			Philantomba maxwellii	Aeschliman (1967); Ntiamoa-Baidu et al. (2004); Ntiamoa-Baidu et al. (2005)
			Philantomba monticola	Pourrut et al. (2011)
			Philantomba sp.	Morel and Mouchet (1965)
			Syncerus caffer	Arthur and Burrow (1957); Elbl and Anastos (1966)
			Tragelaphus eurycerus	Arthur and Burrow (1957); Aeschliman (1967); Cumming (1998)
			Tragelaphus scriptus	Arthur and Burrow (1957); Aeschliman (1967); Ntiamoa-Baidu et al. (2004); Ntiamoa-Baidu et al. (2005)
			Tragelaphus sp.	Cumming (1998); Cumming (1999)
		Suidae	*Potamochoerus porcus*	Arthur and Burrow (1957); Aeschliman (1967); Cumming (1998); Ntiamoa-Baidu et al. (2004); Ntiamoa-Baidu et al. (2005); Pourrut et al. (2011)
			Potamochoerus sp.	Morel and Mouchet (1965); Cumming (1998); Cumming (1999)
			Sus domesticus	Cumming (1998); Ngoy et al. (2021)
		Trangulidae	*Hyemoschus aquaticus*	Morel and Mouchet (1965); Morel (1966); Cumming (1998)
			Tragulus sp.	Arthur and Burrow (1957)
	Carnivora	Canidae	*Canis familiaris*	Arthur and Burrow (1957); Aeschliman (1967); Morel (1966): Cumming (1998); Pourrut et al. (2011)

Species of *Ixodes* (*Afrixodes*)	Host order	Host family	Host species	Reference
		Felidae	*Felis catus*	Aeschliman (1967); Cumming (1998)
			Panthera pardus	Arthur and Burrow (1957); Aeschliman (1967); Cumming (1999)
		Herpestidae	*Atilax paludinosus*	Morel and Mouchet (1965); Aeschliman (1967); Morel (1966)
			Crossarchus obscurus	Aeschliman (1967); Morel (1966)
			Mungos melanurus	Arthur and Burrow (1957)
		Nandiniidae	*Nandinia binotata*	Elbl and Anastos (1966)
		Viverridae	*Civettictis civetta*	Clifford and Anastos (1962); Aeschliman (1967); Ntiamoa-Baidu et al. (2004); Ntiamoa-Baidu et al. (2005)
			Civettictis civetta schwarzi	Elbl and Anastos (1966)
			Genetta genetta hintoni	Clifford and Anastos (1962); Elbl and Anastos (1966)
	Cuculiformeses	Cuculidae	*Centropus senegalensis*	Aeschliman (1967); Cumming (1998)
			Centropus superciliosus	Arthur and Burrow (1957)
	Galliformes	Numididae	*Agelastes niger*	Morel and Mouchet (1965); Morel (1966)
			Guttera plumifera	Morel and Mouchet (1965); Morel (1966)
		Phasianidae	*Gallus gallus*	Aeschliman (1967); Cumming (1998)
	Hyracoidea	Procaviidae	*Dendrohyrax dorsalis*	Aeschliman (1967); Cumming (1998)
			Procavia sp.	Arthur and Burrow (1957); Elbl and Anastos (1966)
	Passeriformes	Turdidae	*Stizorhina fraseri*	Morel and Mouchet (1965); Morel (1966); Cumming (1998)
		Motacillidae	*Anthus trivialis*	Hornok et al. (2022); Cumming (1998)
	Perissodactyla	Equidae	UD	Cumming (1998)
		Rhinocerotidae	UD	Cumming (1998)
	Pholidota	Manidae	*Phataginus tetradactyla*	Aeschliman (1967)
			Phataginus tricuspis	Pourrut et al. (2011)
	Primates	Cercopithecidae	*Cercopithecus diana*	Aeschliman (1966); Morel (1966)

Species of *Ixodes* (*Afrixodes*)	Host order	Host family	Host species	Reference
		Hominoidea	*Homo sapiens*	Arthur and Burrow (1957); Aeschliman (1966); Elbl and Anastos (1966); Cumming (1998)
	Rodentia	Hystricidae	*Atherurus africanus*	Aeschliman (1967); Pourrut et al. (2011)
		Muridae	*Dasymys* sp.	Morel (1966)
			Lemniscomys striatus	Morel (1966)
			Lophuromys sikapusi	Morel (1966)
			Montemys delectorum	Oguge et al. (2009)
			Mus sp.	Morel and Mouchet (1965); Morel (1966); Cumming (1998)
			Tatera sp.	Morel and Mouchet (1965); Morel (1966)
		Nesomyidae	*Cricetomys gambianus*	Arthur and Burrow (1957); Clifford and Anastos (1962); Morel and Mouchet (1965); Aeschliman (1966); Elbl and Anastos (1966); Morel (1966); Colbo and MacLeod (1976);
			Cricetomys sp.	Arthur and Burrow (1957); Ntiamoa-Baidu et al. (2004); Ntiamoa-Baidu et al. (2005)
		Sciuridae	*Funisciurus pyrropus leucostigma*	Aeschliman (1966); Morel (1966)
		Thryonomyidae	*Thryonomys swinderianus*	Ntiamoa-Baidu et al. (2004); Ntiamoa-Baidu et al. (2005)
	Soricomorpha	Soricidae	UD	Cumming (1998)
I. (*A.*) *dawesi*	Afrosoricida	Potamogalidae	*Micropotamogale ruwenzorii*	Elbl and Anastos (1966)
			Potamogale velox	Clifford and Anastos (1962); Elbl and Anastos (1966); Kolonin (2007)
I. (*A.*) *djaronensis*	Carnivora	Viverridae	*Genetta tigrina*	Morel (1965); Kolonin (2007); Guglielmone et al. (2020)
I. (*A.*) *drakensbergensis*	Artiodactyla	Bovidae	*Bos taurus*	Clifford et al. (1975)
			Capra hircus	Clifford et al. (1975)
			Taurotragus oryx	Clifford et al. (1975); Kolonin (2007)
I. (*A.*) *elongatus*	Proboscidea	Elephantidae	UD	Walker (1991)
	Rodentia	Muridae	*Otomys irroratus*	Walker (1991); Cumming (1998); Matthee et al. (2010)
	Soricomorpha	Soricidae	UD	Walker (1991); Cumming (1998)

Species of *Ixodes* (*Afrixodes*)	Host order	Host family	Host species	Reference
I. (*A.*) *euplecti*	Passeriformes	Cisticolidae	*Cisticola chiniana*	Hoogstraal and Wassef (1983)
			Cisticola natalensis	Hoogstraal and Wassef (1983)
		Fringillidae	*Crithagra mozambica*	Hoogstraal and Wassef (1983)
		Malaconotidae	*Tchagra senegalus*	Hoogstraal and Wassef (1983)
			Tchagra sp.	Colbo and MacLeod (1976)
		Motacillidae	*Anthus trivialis*	Hoogstraal and Wassef (1983)
		Muscicapidae	*Oenanthe oenanthe*	Hoogstraal and Wassef (1983)
		Ploceidae	*Euplectes afer*	Hoogstraal and Wassef (1983)
			Euplectes capensis	Hoogstraal and Wassef (1983)
			Euplectes gierowii	Hoogstraal and Wassef (1983)
			Euplectes hordeaceus	Hoogstraal and Wassef (1983)
			Euplectes macroura	Arthur (1956); Hoogstraal and Wassef (1983)
			Ploceus baglafecht	Hoogstraal and Wassef (1983)
		Pycnonotidae	*Atimastillas flavicollis*	Hoogstraal and Wassef (1983)
		Turdidae	*Geokichla piaggiae*	Hoogstraal and Wassef (1983)
			Turdus pelios	Hoogstraal and Wassef (1983)
I. (*A.*) *evansi*	Artiodactyla	Bovidae	*Tragelaphus scriptus*	Cumming (1998); Kolonin (2007)
I. (*A.*) *fynbosensis*	Carnivora	Canidae	*Canis familiaris*	Apanaskevich et al. (2011)
	Rodentia	Muridae	*Rhabdomys pumilio*	Matthee et al. (2010); Apanaskevich et al. (2011)
	Soricomorpha	Soricidae	UD	Apanaskevich et al. (2011)
I. (*A.*) *heinrichi*	Passeriformes	Macrosphenidae	*Melocichla mentalis*	Arthur (1962)
		Sylviidae	UD	Guglielmone et al. (2020)
	Rodentia	Muridae	*Bandicota* sp.	Kolonin (2007)
I. (*A.*) *hyracis*	Hyracoidea	Procaviidae	*Procavia capensis*	Apanaskevich et al. (2025)
I. (*A.*) *latus*	Artiodactyla	Bovidae	UD	Cumming (1998)

Species of *Ixodes* (*Afrixodes*)	Host order	Host family	Host species	Reference
	Hyracoidea	Procaviidae	*Paraxerus lucifer*	Arthur (1956); Guglielmone et al. (2020)
	Rodentia	Sciuridae	UD	Guglielmone et al. (2020)
I. (*A.*) *lemuris*	Primates	Cheirogaleidae	*Cheirogaleus major*	Uilenberg et al. (1979)
			Microcebus sp.	Blanco et al. (2013)
		Indriidae	UD	Kolonin (2007)
		Lemuridae	*Eulemur rufifrons*	Arthur (1956); Arthur (1958); Uilenberg et al. (1979)
	Rodentia	Muridae	*Rattus rattus*	Uilenberg et al. (1979)
I. (*A.*) *lewisi*	Artiodactyla	Bovidae	*Capra hircus*	Cumming (1998)
			Ovis aries	Cumming (1998)
			UD Antilopinae	Cumming (1998); Guglielmone et al. (2020)
			UD Cephalophinae	Cumming (1998)
			UD Neotraginae	Cumming (1998)
			UD Tragelaphinae	Cumming (1998)
		Giraffidae	UD	Cumming (1998)
		Reduncinae	UD	Cumming (1998)
	Carnivora	Canidae	*Canis familiaris*	Cumming (1998)
		Felidae	UD	Cumming (1998); Guglielmone et al. (2020)
	Hyracoidea	Procaviidae	*Heterohyrax brucei*	Colbo and MacLeod (1976); Cumming (1998); Guglielmone et al. (2020)
I. (*A.*) *loveridgei*	Rodentia	Muridae	UD	Cumming (1998); Kolonin (2007)
		Nesomyidae	*Cricetomys gambianus viator*	Arthur (1956)
			Cricetomys sp.	Ntiamoa-Baidu et al. (2004)
I. (*A.*) *lunatus*	Afrosoricida	Tenrecidae	*Hemicentetes semispinosus*	Uilenberg et al. (1979)
			Setifer setosus	Uilenberg et al. (1979)
			Tenrec ecaudatus	Uilenberg et al. (1979)

Species of *Ixodes* (*Afrixodes*)	Host order	Host family	Host species	Reference
			Tenrec sp.	Kolonin (2007); Apanaskevich and Goodman (2020)
	Rodentia	Nesomyidae	*Nesomys audeberti*	Uilenberg et al. (1979)
I. (*A.*) *macfarlanei*	Artyodactyla	Bovidae	*Cephalophus silvicultor*	Keirans et al. (1982); Guglielmone et al. (2020)
	Carnivora	Viverridae	*Genetta genetta*	Keirans et al. (1982); Guglielmone et al. (2020)
			Genetta maculata	Keirans et al. (1982); Guglielmone et al. (2020)
			Genetta tigrina	Keirans et al. (1982); Guglielmone et al. (2020)
I. (*A.*) *matopi*	Artyodactyla	Bovidae	*Capra hircus*	Spickett et al. (1981)
			Oreotragus oreotragus	Colborne et al. (1981); Spickett et al. (1981); Cumming (1998); Kolonin (2007)
			UD Neotraginae	Cumming (1998);Kolonin (2007)
	Hyracoidea	Procaviidae	*Heterohyrax brucei*	Colborne et al. (1981); Cumming (1998)
			Procavia capensis	Colborne et al. (1981); Cumming (1998)
	Lagomorpha	Leporidae	*Pronolagus crassicaudatus*	Colborne et al. (1981); Cumming (1998)
I. (*A.*) *microgalei*	Afrosoricida	Tenrecidae	*Microgale parvula*	Apanaskevich et al. (2013)
			Microgale soricoides	Apanaskevich et al. (2013)
			Nesogale dobsoni	Apanaskevich et al. (2013); Apanaskevich and Goodman (2020)
I. (*A.*) *minutae*	Rodentia	UD	UD	Kolonin (2007)
I. (*A.*) *moreli*	Artyodactyla	Bovidae	*Cephalophus dorsalis*	Aeschliman (1966); Ntiamoa-Baidu et al. (2004); Ntiamoa-Baidu et al. (2005)
			Cephalophus niger	Aeschliman (1966); Ntiamoa-Baidu et al. (2004); Ntiamoa-Baidu et al. (2005)
			Cephalophus rufilatus	Ntiamoa-Baidu et al. (2004)
			Cephalophus sp.	Cumming (1998); Cumming (1999)
			Cephalophus zebra	Aeschliman (1966)
			Neotragus pygmaeus	Cumming (1998); Ntiamoa-Baidu et al. (2005)
			Philantomba maxwellii	Ntiamoa-Baidu et al. (2004); Ntiamoa-Baidu et al. (2005)

Species of *Ixodes* (*Afrixodes*)	Host order	Host family	Host species	Reference
			Tragelaphus scriptus	Aeschliman (1966); Elbl and Anastos (1966); Ntiamoa-Baidu et al. (2004)
			Tragelaphus sp.	Cumming (1998); Cumming (1999)
	Carnivora	Felidae	*Felis catus*	Aeschliman (1966); Cumming (1998)
			Panthera pardus	Aeschliman (1966); Cumming (1998)
		Nandiniidae	*Nandinia binotata*	Aeschliman (1966)
		Viverridae	*Civettictis civetta*	Aeschliman (1966); Ntiamoa-Baidu et al. (2005)
	Hyracoidea	Hyracoidea	UD	Cumming (1998)
	Rodentia	Thryonomyidae	*Thryonomys swinderianus*	Ntiamoa-Baidu et al. (2005)
I. (*A.*) *muniensis*	Artiodactyla	Bovidae	*Bos taurus*	Aeschliman (1966)
			Capra hircus	Aeschliman (1966); Elbl and Anastos (1966); Cumming (1998)
			Cephalophus dorsalis	Aeschliman (1966); Ntiamoa-Baidu et al. (2005)
			Cephalophus dorsalis castaneus	Morel and Mouchet (1965)
			Cephalophus niger	Aeschliman (1966); Elbl and Anastos (1966); Ntiamoa-Baidu et al. (2004)
			Cephalophus nigrifrons nigrifrons	Elbl and Anastos (1966)
			Cephalophus rufilatus	Ntiamoa-Baidu et al. (2005)
			Cephalophus sp.	Arthur and Burrow (1957); Morel and Mouchet (1965); Aeschliman (1966); Elbl and Anastos (1966); Cumming (1998); Pourrut et al. (2011)
			Neotragus batesi	Morel and Mouchet (1965); Cumming (1998)
			Neotragus pygmaeus	Arthur and Burrow (1957); Aeschliman (1966); Morel (1966); Cumming (1998); Ntiamoa-Baidu et al. (2004); Ntiamoa-Baidu et al. (2005)
			Ovis aries	Aeschliman (1966); Cumming (1998)
			Philantomba maxwellii	Aeschliman (1966); Morel (1966); CNtiamoa-Baidu et al. (2004); Ntiamoa-Baidu et al. (2005)
			Philantomba sp.	Morel and Mouchet (1965)

Species of *Ixodes* (*Afrixodes*)	Host order	Host family	Host species	Reference
			Tragelaphus eurycerus	Arthur and Burrow (1957); Aeschliman (1966); Cumming (1998)
			Tragelaphus scriptus	Aeschliman (1966); Elbl and Anastos (1966); Cumming (1998); Ntiamoa-Baidu et al. (2004); Ntiamoa-Baidu et al. (2005)
		Giraffidae	*Okapia* sp.	Elbl and Anastos (1966); Cumming (1998)
		Suidae	*Potamochoerus porcus*	Elbl and Anastos (1966)
			Sus domesticus	Elbl and Anastos (1966); Cumming (1998)
			UD	Cumming (1998)
	Carnivora	Canidae	*Canis familiaris*	Aeschliman (1966); Morel (1966); Cumming (1998)
		Felidae	*Felis catus*	Arthur and Burrow (1957); Morel and Mouchet (1965); Aeschliman (1966); Cumming (1998)
			Panthera pardus	Arthur and Burrow (1957); Morel and Mouchet (1965); Aeschliman (1966); Elbl and Anastos (1966); Morel (1966)
		Herpestidae	*Crossarchus obscurus*	Aeschliman (1966); Morel (1966)
		Viverridae	*Civettictis civetta*	Aeschliman (1966); Ntiamoa-Baidu et al. (2004); Ntiamoa-Baidu et al. (2005)
	Hyracoidea	Procaviidae	*Dendrohyrax* sp.	Aeschliman (1966); Cumming (1998)
	Primates	Hominoidea	*Homo sapiens*	Aeschliman (1966); Lacroux et al. (2023)
	Rodentia	Gliridae	*Graphiurus microtis*	Oguge et al. (2009)
		Muridae	*Uranomys ruddi*	Morel (1966); Cumming (1998)
		Nesomyidae	*Cricetomys gambianus*	Aeschliman (1966); Elbl and Anastos (1966)
		Sciuridae	*Heliosciurus gambianus*	Aeschliman (1966); Morel (1966)
		Thryonomyidae	*Thryonomys swinderianus*	Ntiamoa-Baidu et al. (2004); Ntiamoa-Baidu et al. (2005)
I. (*A.*) *myotomys*	Carnivora	Felidae	UD	Guglielmone et al. (2020)
	Rodentia	Muridae	*Otomys unisulcatus*	Walker (1991); Cumming (1998); Kolonin (2007)
I. (*A.*) *nairobiensis*	Artiodactyla	Suidae	*Phacochoerus aethiopicus*	Arthur (1959); Elbl and Anastos (1966); Cumming (1998)

Species of *Ixodes* (*Afrixodes*)	Host order	Host family	Host species	Reference
	Carnivora	Canidae	*Canis familiaris*	Nuttall (1916); Elbl and Anastos (1966); Cumming (1998)
		Viverridae	*Civettictis civetta schwarzi*	Arthur (1959); Elbl and Anastos (1966); Cumming (1998)
	Rodentia	Muridae	*Micaelamys namaquensis*	Arthur (1959); Elbl and Anastos (1966); Cumming (1998); Kolonin (2007)
			Arvicanthis niloticus	Clifford and Anastos (1962); Elbl and Anastos (1966); Cumming (1998); Kolonin (2007)
			Mastomys natalensis ismailiae	Hoogstraal (1956); Elbl and Anastos (1966); Cumming (1998); Kolonin (2007)
			Mastomys sp.	Hoogstraal (1956); Elbl and Anastos (1966); Cumming (1998); Kolonin (2007)
			Pelomys fallax concolor	Arthur (1959); Elbl and Anastos (1966); Cumming (1998); Kolonin (2007)
	Soricomorpha	Soricidae	*Crocidura* sp.	Elbl and Anastos (1966); Cumming (1998)
I. (*A.*) *nchisiensis*	Macroscelidea	Macroscelididae	*Petrodromus tetradactylus*	Arthur (1956); Kolonin (2007)
	Rodentia	Muridae	*Arvicanthis niloticus*	Elbl and Anastos (1966); Theiler (1962); Keirans et al. (1982); Cumming (1998)
	Soricomorpha	Soricidae	*Crocidura flavescens*	Keirans et al. (1982); Cumming (1998)
			Crocidura flavescens occidentalis	Morel (1966)
I. (*A.*) *neitzi*	Artiodactyla	Bovidae	*Aepyceros melampus*	Walker (1991); Cumming (1998); Kolonin (2007)
			Oreotragus oreotragus	Rechav et al. (1978); Cumming (1998); Kolonin (2007)
			Redunca fulvorufula	Clifford, Walker, Keirans (1977); Cumming (1998); Kolonin (2007)
	Carnivora	Felidae	*Panthera pardus*	Baauw et al. (2019); Ledwaba et al. (2022)
		Hyaenidae	*Hyaena brunnea*	Baauw et al. (2019); Ledwaba et al. (2022)
I. (*A.*) *nesomys*	Rodentia	Muridae	UD	Kolonin (2007); Apanaskevich and Goodman (2020)
		Nesomyidae	*Nesomys rufus*	Uilenberg and Hoogstraal (1969); Uilenberg et al. (1979); Apanaskevich and Goodman (2020)
I. (*A.*) *nicolasi*	Artiodactyla	Bovidae	UD	Guglielmone et al. (2023)
I. (*A.*) *okapiae*	Artiodactyla	Giraffidae	*Okapia johnstoni*	Arthur (1956); Elbl and Anastos (1966); Cumming (1998); Kolonin (2007)
I. (*A.*) *oldi*	Artiodactyla	Bovidae	*Bos taurus*	Aeschliman (1966); Cumming (1998)

Species of *Ixodes* (*Afrixodes*)	Host order	Host family	Host species	Reference
			Cephalophus niger	Aeschliman (1966); Cumming (1998)
	Carnivora	Canidae	*Canis familiaris*	Aeschliman (1966); Cumming (1998); Cumming (1999)
		Felidae	*Felis catus*	Aeschliman (1966); Keirans et al. (1982); Cumming (1998);
			Leptailurus serval	Arthur (1956); Keirans et al. (1982)
			Panthera pardus	Aeschliman (1966); Cumming (1999)
		Nandiniidae	*Nandinia binotata*	Keirans et al. (1982)
		Viverridae	*Civettictis civetta*	Arthur (1956); Aeschliman (1966); Keirans et al. (1982); Ntiamoa-Baidu et (2004); Ntiamoa-Baidu et al. (2005)
			Genetta genetta	Keirans et al. (1982)
			Genetta genetta neumanni	Arthur (1956); Keirans et al. (1982)
			Genetta maculata	Aeschliman (1966)
			Genetta sp.	Arthur (1956); Aeschliman (1966); Keirans et al. (1982); Cumming (1998); Kolonin (2007)
	Macroscelidea	Macroscelididae	*Petrodromus tetradactylus*	Keirans et al. (1982)
	Pholidota	Manidae	*Phataginus longicaudata*	Aeschliman (1966); Cumming (1998)
	Rodentia	Muridae	*Mus musculoides*	Elbl and Anastos (1966); Cumming (1998)
		Nesomyidae	*Cricetomys gambianus*	Aeschliman (1966)
		Sciuridae	*Paraxerus* sp.	Arthur (1956)
		sp.alacidae	*Tachyoryctes* sp.	Arthur (1956)
	Soricomorpha	Soricidae	*Crocidura flavescens occidentalis*	Elbl and Anastos (1966)
			Crocidura sp.	Elbl and Anastos (1966); Cumming (1998)
I. (*A.*) *pilosus*	Artiodactyla	Bovidae	*Bos taurus*	Clifford et al. (1975); Horak et al. (1987); Cumming (1998)
			Capra hircus	Cumming (1998)
			Ovis aries	Cumming (1998)
			Pelea capreolus	Horak et al. (1987); Walker (1991)

Species of *Ixodes* (*Afrixodes*)	Host order	Host family	Host species	Reference
			Redunca sp.	Cumming (1998); Cumming (1999)
			Sylvicapra grimmia	Walker (1991)
			Tragelaphus scriptus	Walker (1991)
			Tragelaphus sp.	Horak et al. (1987); Cumming (1998); Cumming (1999)
	Carnivora	Canidae	*Canis familiaris*	Clifford et al. (1975); Horak et al. (1987); Cumming (1998)
			Lupulella mesomelas	Walker (1991)
			Otocyon megalotis	Walker (1991)
		Felidae	*Caracal caracal*	Walker (1991)
			Felis sp.	Cumming (1999)
			Leptailurus serval	Bedford (1936)
		Herpestidae	*Herpestidae* sp.	Cumming (1999)
	Lagomorpha	Leporidae	*Lepus saxatilis*	Horak et al. (1987); Walker (1991); Cumming (1998); Cumming (1999)
	Primates	Galagidae	UD	Cumming (1998)
		Hominoidea	UD	Cumming (1998)
	Rodentia	Muridae	UD	Horak et al. (1987); Cumming (1998)
I. (*A.*) *procaviae*	Artiodactyla	Bovidae	*Antidorcas marsupiali*	Golezardy and Horak (2007); Horak et al. (2007); Ledwaba et al. (2022)
			Connochaetes gnou	Golezardy and Horak (2007); Ledwaba et al. (2022)
			Damaliscus pygargus dorcas	Golezardy and Horak (2007); Horak et al. (2007); Ledwaba et al. (2022)
			Oryx gazella	Golezardy and Horak (2007); Ledwaba et al. (2022)
			Pelea capreolus	Golezardy and Horak (2007); Ledwaba et al. (2022)
			Redunca fulvorufula	Golezardy and Horak (2007); Ledwaba et al. (2022)
			Taurotragus oryx	Cumming (1998); Golezardy and Horak (2007); Ledwaba et al. (2022)
			Tragelaphus sp.	Horak et al. (2007); Ledwaba et al. (2022)

Species of *Ixodes* (*Afrixodes*)	Host order	Host family	Host species	Reference
		Suidae	*Phacochoerus* sp.	Arthur and Burrow (1957); Cumming (1998)
	Carnivora	Canidae	*Canis familiaris*	Horak et al. (1987)
			Lupulella mesomelas	Horak et al. (1987)
			Otocyon megalotis	Horak et al. (1987)
		Felidae	*Caracal caracal*	Horak et al. (2000); Viljoen et al. (2020); Ledwaba et al. (2022)
			Felis silvestris lybica	Horak et al. (2000)
			Panthera leo	Horak et al. (2000); Ledwaba et al. (2022)
		Herpestidae	*Herpestes sanguinea*	Horak et al. (2000); Ledwaba et al. (2022)
		Hyaenidae	*Crocuta crocuta*	Horak et al. (2000); Ledwaba et al. (2022)
			Proteles cristatus	Horak et al. (1987)
		Viverridae	*Civettictis civetta*	Horak et al. (2000); Ledwaba et al. (2022)
			Genetta genetta	Horak et al. (1987); Horak et al. (2000); Ledwaba et al. (2022)
			Genetta sp.	Horak et al. (2000); Ledwaba et al. (2022)
			Genetta tigrina	Horak et al. (2000); Ledwaba et al. (2022)
	Hyracoidea	Procaviae	*Dendrohyrax arboreus*	Arthur and Burrow (1957); Morel (1965); Cumming (1998); Kolonin (2007)
			Procavia capensis	Walker (1991); Golezardy and Horak (2007); Ledwaba et al. (2022)
			Procavia capensis johnstoni	Elbl and Anastos (1966)
	Lagomorpha	Leporidae	*Pronolagus rupestris*	Golezardy and Horak (2007); Ledwaba et al. (2022)
	Passeriformes	Cisticolidae	*Cisticola tinniens*	Hasle et al. (2009)
		Ploceidae	*Euplectes afer*	Hasle et al. (2009)
	Rodentia	Muridae	*Otomys irroratus*	Matthee et al. (2010); Ledwaba et al. (2022)
			Rhabdomys pumilio	Matthee et al. (2010); Ledwaba et al. (2022)
I. (*A.*) *radfordi*	Carnivora	Herpestidae	UD	Bhat and Srinivasan (1981); Muraleedharan (2017)

Species of *Ixodes* (*Afrixodes*)	Host order	Host family	Host species	Reference
	Rodentia	Muridae	*Rattus rattus rufescens*	Kohls (1947); Guglielmone et al. (2020)
I. (*A.*) *rageaui*	Primates	Cercopithecidae	*Cercopithecus cephus*	Arthur (1956); Hoogstraal and Theiler (1959); Morel and Mouchet (1965); Kolonin (2007); Pourrut et al. (2011)
			Cercopithecus mitis	Hoogstraal and Theiler (1959); Elbl and Anastos (1966); Kolonin (2007)
		Hominoidea	*Homo sapiens*	Morel and Mouchet (1965); Cumming (1998); Cumming (1999); Kolonin (2007)
I. (*A.*) *randrianasoloi*	Afrosoricida	Tenrecidae	*Nesogale talazaci*	Uilenberg et al. (1979); Kolonin (2007); Apanaskevich and Goodman (2020); Guglielmone et al. (2020)
	Rodentia	Muridae	*Rattus rattus*	Uilenberg et al. (1979); Kolonin (2007); Apanaskevich and Goodman (2020); Guglielmone et al. (2020)
I. (*A.*) *rasus*	Artiodactyla	Bovidae	*Capra hircus*	Aeschliman (1966); Cumming (1998)
			Cephalophus dorsalis	Cumming (1998); Pourrut et al. (2011)
			Cephalophus leucogaster	Cumming (1998); Arthur and Burrow (1957)
			Cephalophus nigrifrons nigrifrons	Cumming (1998); Pourrut et al. (2011)
			Philantomba maxwellii	Aeschliman (1966)
			Philantomba monticola	Pourrut et al. (2011)
			Neotragus batesi	Morel and Mouchet (1965); Cumming (1998)
			Neotragus pygmaeus	Arthur and Burrow (1957); Aeschliman (1966); Elbl and Anastos (1966); Cumming (1998)
			Ovis aries	Cumming (1998)
			Tragelaphus eurycerus	Morel and Mouchet (1965); Cumming (1998)
		Giraffidae	*UD*	Cumming (1998)
		Trangulidae	*Hyemoschus aquaticus*	Aeschliman (1966); Cumming (1998)
		Suidae	*Potamochoerus porcus*	Cumming (1998); Pourrut et al. (2011)
			Potamochoerus sp.	Arthur and Burrow (1957); Morel and Mouchet (1965); Cumming (1998)
	Carnivora	Canidae	*Canis familiaris*	Morel and Mouchet (1965); Aeschliman (1966); Cumming (1998)
		Felidae	*Panthera pardus*	Arthur and Burrow (1957)

Species of *Ixodes* (*Afrixodes*)	Host order	Host family	Host species	Reference
		Herpestidae	*Atilax paludinosus*	Morel and Mouchet (1965); Pourrut et al. (2011)
			Crossarchus obscurus	Aeschliman (1966)
			Herpestidae sp.	Arthur and Burrow (1957)
			Ichneumia albicauda	Arthur and Burrow (1957)
		Nandiniidae	*Nandinia binotata*	Arthur and Burrow (1957); Aeschliman (1966); Pourrut et al. (2011)
		Viverridae	*Civettictis civetta*	Arthur and Burrow (1957); Ntiamoa-Baidu et al. (2004); Ntiamoa-Baidu et al. (2005); Pourrut et al. (2011)
			Genetta tigrina	Arthur and Burrow (1957)
	Galliformes	Numididae	*Agelastes niger*	Morel and Mouchet (1965); Cumming (1998)
			Guttera plumifera	Morel and Mouchet (1965); Cumming (1998)
			Numida meleagris	Morel and Mouchet (1965); Cumming (1998)
		Phasianidae	*Pternistis bicalcaratus*	Morel and Mouchet (1965); Cumming (1998)
	Hyracoidea	Procaviidae	*Dendrohyrax arboreus*	; Elbl and Anastos (1966)
			Dendrohyrax dorsalis	Aeschliman (1966)
			UD	Arthur and Burrow (1957); Cumming (1998)
	Lagomorpha	Leporidae	*Lepus victoriae microtis*	Hoogstraal (1956)
	Macroscelidea	Macroscelididae	*Rhynchocyon cirnei*	Colbo and MacLeod (1976)
	Rodentia	Hystricidae	*Atherurus africanus*	Elbl and Anastos (1966); Ntiamoa-Baidu et al. (2004); Ntiamoa-Baidu et al. (2005); Pourrut et al. (2011)
		Muridae	*Dasymys* sp.	Elbl and Anastos (1966); Cumming (1998)
			Lophuromys aquilus	Arthur and Burrow (1957); Cumming (1998)
		Nesomyidae	*Cricetomys gambianus*	Aeschliman (1966)
		Thryonomyidae	*Thryonomys swinderianus*	Arthur and Burrow (1957); Ntiamoa-Baidu et al. (2004); Ntiamoa-Baidu et al. (2005)
	Passeriformes	Turdidae	*Stizorhina fraseri*	Morel and Mouchet (1965)
	Pholidota	Manidae	*Phataginus tricuspis*	Arthur and Burrow (1957); Morel and Mouchet (1965); Cumming (1998); Pourrut et al. (2011)

Species of *Ixodes* (*Afrixodes*)	Host order	Host family	Host species	Reference
	Primates	Cercopithecidae	*Cercocebus albigena*	Arthur and Burrow (1957); Hoogstraal and Theiler (1959); Cumming (1998)
			Cercopithecus ascanius	Elbl and Anastos (1966); Cumming (1998)
			Piliocolobus badius	Arthur and Burrow (1957);Hoogstraal and Theiler (1959); Cumming (1998)
		Hominoidea	*Homo sapiens*	Aeschliman (1966); Cumming (1998)
	Testudines	Testudinoidea	*Kinixys erosa*	Pourrut et al. (2011)
I. (*A.*) *rhabdomysae*	Macroscelidea	Macroscelididae	*Petrodromus tetradactylus*	Colbo and MacLeod (1976)
	Rodentia	Muridae	*Aethomys chrysophilus*	Colbo and MacLeod (1976); Cumming (1998)
			Aethomys kaiseri walambae	Colbo and MacLeod (1976); Cumming (1998)
			Grammomys dolichurus	Colbo and MacLeod (1976); Cumming (1998)
			Mastomys natalensis	Colbo and MacLeod (1976); Cumming (1998)
			Mus triton	Colbo and MacLeod (1976); Cumming (1998)
			Pelomys fallax	Colbo and MacLeod (1976); Cumming (1998)
			Rhabdomys pumilio	Walker (1991); Cumming (1998)
			Zelotomys hildegardeae	Colbo and MacLeod (1976); Cumming (1998)
		Nesomyidae	*Saccostomus campestris*	Colbo and MacLeod (1976); Cumming (1998)
	Soricomorpha	Soricidae	*Crocidura hirta*	Colbo and MacLeod (1976)
I. (*A.*) *rotundatus*	Artiodactyla	Bovidae	*Tragelaphus scriptus*	Arthur (1956); Cumming (1998)
	Rodentia	sp.alacidae	*Tachyoryctes ruandae*	Elbl and Anastos (1966)
			Tachyoryctes sp.	Arthur (1956); Cumming (1998); Cumming (1999)
I. (*A.*) *rubicundus*	Artiodactyla	Bovidae	*Alcelaphus buselaphus caama*	Tonetti et al. (2009); Ledwaba et al. (2022)
			Antidorcas marsupialis	Horak et al. (1987); Tonetti et al. (2009); Berggoetz et al. (2014); Ledwaba et al. (2022)
			Bos indicus	Berggoetz et al. (2014)
			Bos taurus	Berggoetz et al. (2014)

Species of *Ixodes* (*Afrixodes*)	Host order	Host family	Host species	Reference
			Capra hircus	Clifford et al. (1975); Cumming (1998); Cumming (1999)
			Connochaetes gnou	Horak et al. (1987)
			Damaliscus pygargus phillipsi	Cumming (1998); Tonetti et al. (2009); Ledwaba et al. (2022)
			Ovis aries	Clifford et al. (1975); Cumming (1998); Cumming (1999); Berggoetz et al. (2014); Horak et al. (2015); Ledwaba et al. (2022)
			Redunca fulvorufula	Horak et al. (1987); Walker (1991); Cumming (1998)
			Taurotragus oryx	Walker (1991); Cumming (1998); Horak et al. (2005); Berggoetz et al. (2014); Ledwaba et al. (2022)
			Tragelaphus strepsiceros	Tonetti et al. (2009); Horak et al. (2015); Ledwaba et al. (2022)
			Tragelaphus sp.	Horak et al. (2005); Ledwaba et al. (2022)
	Carnivora	Canidae	*Canis familiaris*	Cumming (1998)
			Lupulella mesomelas	Horak et al. (2000); Ledwaba et al. (2022)
		Felidae	*Caracal caracal*	Horak et al. (1987); Walker (1991); Cumming (1999); Horak et al. (2005); Berggoetz et al. (2014); Ledwaba et al. (2022)
			Felis catus	Clifford et al. (1975); Cumming (1999)
			Felis silvestris lybica	Cumming (1999); Horak et al. (2000); Viljoen et al. (2020); Ledwaba et al. (2022)
	Lagomorpha	Leporidae	*Lepus saxatilis*	Horak et al. (1987); Cumming (1999)
			Pronolagus rupestris	Horak et al. (1987); Walker (1991); Cumming (1999)
	Macroscelidea	Macroscelididae	*Elephantulus myurus*	Walker (1991); Horak et al. (2000); Horak et al. (2015); Ledwaba et al. (2022)
	Perissodactyla	Equidae	*Equus zebra zebra*	Cumming (1998); Horak et al. (2007); Ledwaba et al. (2022)
		Rhinocerotidae	*Ceratotherium simum*	Cumming (1998); Tonetti et al. (2009); Ledwaba et al. (2022)
	Rodentia	Muridae	*Aethomys namaquensis*	Horak et al. (1987); Cumming (1998); Kolonin (2007)
			Otomys irroratus	Cumming (1998); Kolonin (2007); Horak et al. (2005); Matthee et al. (2007)

Species of *Ixodes* (*Afrixodes*)	Host order	Host family	Host species	Reference
			Otomys unisulcatus	Horak et al. (1987); Cumming (1998); Kolonin (2007)
			Rhabdomys pumilio	Horak et al. (1987); Cumming (1998); Kolonin (2007)
		Nesomyidae	*Saccostomus campestris*	Horak et al. (1987)
I. (*A.*) *schillingsi*	Primates	Cercopithecidae	*Colobus caudatus*	Arthur (1956); Guglielmone et al. (2020)
			Colobus polykomos caudatus	Hoogstraal and Theiler (1959); Guglielmone et al. (2020)
			Colobus polykomos dodingae	Hoogstraal (1956); Hoogstraal and Theiler (1959); Guglielmone et al. (2020)
			Colobus polykomos matschei	Hoogstraal and Theiler (1959); Guglielmone et al. (2020)
			Colobus sp.	Arthur (1956); Hoogstraal and Theiler (1959); Kolonin (2007); Guglielmone et al. (2020)
		Galagidae	*Otolemur crassicaudatus*	Hoogstraal and Theiler (1959); Guglielmone et al. (2020)
		Hominoidea	*Homo sapiens*	Hoogstraal and Theiler (1959); Guglielmone et al. (2020)
	Rodentia	Cricetidae	*Lophiomys imhausi*	Hoogstraal and Theiler (1959); Guglielmone et al. (2020)
I. (*A.*) *soarimalalae*	Afrosoricida	Tenrecidae	*Hemicentetes nigriceps*	Apanaskevich and Goodman (2020)
			Microgale cowani	Apanaskevich and Goodman (2020)
			Microgale soricoides	Apanaskevich and Goodman (2020)
			Nesogale dobsoni	Apanaskevich and Goodman (2020)
			Oryzorictes hova	Apanaskevich and Goodman (2020)
I. (*A.*) *spinae*	Hyracoidea	Procaviae	*Procavia capensis*	Arthur (1956); Clifford and Anastos (1962); Morel (1965); Cumming (1998); Kolonin (2007)
	Passeriformes	Motacillidae	*Anthus trivialis*	Clifford and Anastos (1962); Cumming (1998)
		Ploceidae	*Euplectes orix*	Cumming (1998); Hasle et al. (2009)
	Rodentia	UD	UD	Clifford and Anastos (1962); Cumming (1998)
I. (*A.*) *thomasae*	Cuculiformes	Cuculidae	UD	Guglielmone et al. (2020)
	Passeriformes	Malaconotidae	UD	Cumming (1998); Guglielmone et al. (2020)
		Ploceidae	UD	Cumming (1998); Guglielmone et al. (2020)
	Rodentia	Muridae	*Arvicanthis abyssinicus*	Arthur and Burrow (1957); Cumming (1998); Kolonin (2007)
			Mastomys natalensis	Colbo and MacLeod (1976); Cumming (1998); Kolonin (2007)

Species of *Ixodes* (*Afrixodes*)	Host order	Host family	Host species	Reference
			Mastomys sp.	Elbl and Anastos (1966); Cumming (1998); Kolonin (2007)
			Otomys tropicalis elgonis	Arthur and Burrow (1957); Cumming (1998); Kolonin (2007)
			Pelomys fallax	Colbo and MacLeod (1976); Cumming (1998); Kolonin (2007)
I. (*A.*) *transvaalensis*	Rodentia	Muridae	*Aethomys namaquensis*	Walker (1991); Kolonin (2007)
I. (*A.*) *ugandanus*	Artiodactyla	Bovidae	*Capra hircus*	Cumming (1998)
			Ovis aries	Cumming (1998)
			UD	Cumming (1998); Guglielmone et al. (2020)
	Carnivora	Herpestidae	UD	Guglielmone et al. (2020)
		Viverridae	UD	Cumming (1998); Guglielmone et al. (2020)
	Primates	Lorisidae	UD	Cumming (1998); Guglielmone et al. (2020)
	Rodentia	Thryonomyidae	*Thryonomys swinderianus*	Clifford, Walker, Keirans (1977); Walker (1991); Cumming (1998); Kolonin (2007); Guglielmone et al. (2020)
I. (*A.*) *uilenbergi*	Afrosoricida	Tenrecidae	*Hemicentetes nigriceps*	Apanaskevich and Goodman (2020)
			Microgale soricoides	Apanaskevich and Goodman (2020)
			Nesogale dobsoni	Apanaskevich and Goodman (2020)
			Oryzorictes hova	Apanaskevich and Goodman (2020)
I. (*A.*) *uncus*	Afrosoricida	Tenrecidae	*Microgale soricoides*	Apanaskevich and Goodman (2020)
			Nesogale dobsoni	Apanaskevich and Goodman (2020)
I. (*A.*) *vanidicus*	Artiodactyla	Bovidae	UD	Cumming (1998); Guglielmone et al. (2020)
	Carnivora	Canidae	*Canis familiaris*	Morel and Mouchet (1965); Cumming (1998); Guglielmone et al. (2020)
		Herpestidae	*Atilax paludinosus*	Morel and Mouchet (1965); Keirans et al. (1982); Guglielmone et al. (2020)
		Viverridae	*Civettictis* sp.	Morel and Mouchet (1965); Keirans et al. (1982); Cumming (1998); Kolonin (2007)
	Macroscelidea	Macroscelididae	UD	Guglielmone et al. (2020)
	Primates	Hominoidea	*Homo sapiens*	Guglielmone et al. (2020)

Species of *Ixodes* (*Afrixodes*)	Host order	Host family	Host species	Reference
I. (*A.*) *walkerae*	Carnivora	Herpestidae	UD	Guglielmone et al. (2020)
		Viveriidae	UD	Guglielmone et al. (2020)
	Galliformes	Numididae	UD	Guglielmone et al. (2020)
	Passeriformes	Turdiadae	*Turdus olivaeus*	Clifford, Kohls, Hoogstraal (1968); Guglielmone et al. (2020)
I. (*A.*) *zairensis*	Soricomorpha	Soricidae	*Crocidura flavescens*	Keirans et al. (1982); Kolonin (2007)
			Crocidura munissii	Keirans et al. (1982); Kolonin (2007)
			Crocidura sp.	Keirans et al. (1982); Kolonin (2007)

Abbreviation: UD, undetermined.

References

1 Apanaskevich, D. and Goodman, S. M. Description of three new species of *Ixodes* Latreille, 1795 (Acari: Ixodidae), parasites of tenrecs (Afrotheria: Tenrecidae) on Madagascar. Systematic Parasitology, 97, 623-637 (2020).

2 Uilenberg, G., Hoogstraal, H. and Klein, J.M. Les tiques (Ixodidae) de Madagascar et leur rôle vecteur. Archives de l'Institut Pasteur de Madagascar. Numéro spécial, 154pp (1979).

3 Englert, M.C., Goodman, S.M. and Apanaskevich, D.A. Description of a new species of *Ixodes* Latreille, 1795 (Acari: Ixodidae), parasite of shrew tenrecs (Afrotheria:Tenrecidae) and rodents (Rodentia: Muridae) on Madagascar. Systematic Parasitology, 100: 745-750 (2023).

4 Apanaskevich, D., Soarimalala, V. and Goodman, S. A new Ixodes species (Acari: Ixodidae), parasite of shrew tenrecs (Afrosoricida: Tenrecidae) in Madagascar. Journal Parasitology, 99(6), 970-972 (2013).

5 Kolonin, G.V. Mammals as Hosts of Ixodid Ticks (Acarina, Ixodidae). Entomological Review, 87(4), 401-412 (2007).

6 Elbl, A. and Anastos, G. Ixodid ticks (Acarina, Ixodidae) of Central Africa. Musée Royal de l'Afrique Centrale, Tervuen, Belgique annales, Serie in-8°, sciences zoologiques, n°146, 209 pp (1966).

7 Clifford, C.M. and Anastos, G. Exploration du parc national de l'Upembe, Mission GF de White. Institut des parcs nationaux du Congo et du Rwanda. IBERSOM, Ticks (66), 72pp (1962).

8 Walker, J.B. A review of the ixodid ticks (Acari, Ixodidae) occurring in southern Africa. Onderstepoort Journal Veterinary Research, 58, 81-105 (1991).

9 Cumming, G.S. Host preference in African ticks (Acari: Ixodida): a quantitative data set. Bulletin of Entomological Research, 88, 379-406 (1998).

10 Tonetti, N., et al. Ticks and Ticks-Borne pathogens from wildlife in the free state province, South Africa. Journal of Wildlife Diseases, 45(2), 437-446 (2009).

11 Ledwaba, M.B., et al. Distribution and prevalence of ticks and tick-borne pathogens of wild animals in South Africa: A systematic review. Current Research in Parasitology & Vector-Borne Diseases, 2, 100088 (2022).

12 Golezardy, H. and Horak, I.G. Ticks (Acari: Ixodidae) collected from animals in three western, semi-arid nature reserves in South Africa. Onderstepoort Journal of Veterinary Research, 74:81–85 (2007).

13 Horak, I.G., Golezardy, H. and Uys, A.C. Ticks associated with the three largest wild ruminant species in southern Africa. Onderstepoort Journal of Veterinary Research, 74, 231-242 (2007).

14 Horak, I.G., Moolman, L.C. and Fourie, L.J. Some wild hosts of the Karoo paralysis tick, *Ixodes rubicundus* Neumann 1904 (Acari: Ixodidae). Onderstepoort journal of Veterinary Research, 54, 49-51 (1987).

15 Berggoetz, M., et al. Protozoan and bacterial pathogens in tick salivary glands in wild and domestic animal environments in South Africa. Ticks and Tick-Borne Diseases, 5, 176-185 (2014).

16 Clifford,C.M., Theiler, G. and Baker, M. *Ixodes* (*Afrixodes*) *drakensbergensis* n. sp. from domestic and wild animals in Natal, Republic of South Africa. Onderstepport Journal of Veterinary Research Institute, 42(1), 33-40 (1975).

17 Aeschlimann, A. Biologie et écologie des tiques (Ixodidae) de Côte d'Ivoire. Acta Tropica, 24, 281-405 (1967).

18 Horak, I.G., Jacot Guillarmod, A., Moolman, L.C. and De Vos, V. Parasites of domestic and wild animals in South Africa. XXII. Ixodid ticks on domestic dogs and on wild carnivores. Onderstepoort Journal of Veterinary Research, 54, 573-580 (1987).

19 Arthur, D.R. and Burrow, C. The *Ixodes rasus* group of African tick with descriptions of four new species (Ixodoidea, Ixodidae). Bulletin of the Museum of Comparative Zoology, 116-9, 48pp (1957).

20 Spickett, A.M., Keirans, J.E., Norval, R.A.I and Clifford, C.M. *Ixodes* (*Afrixodes*) *matopi* n. sp. (Acarina: Ixodidae): a tick found aggregating on pre-orbital gland scent marks of the klipspringer in Zimbabwe. Onderstepoort journal of Veterinary Research, 48, 23-30 (1981).

21 Cumming, G.S. Host distribution do not limit the species ranges of most african ticks (Acari: Ixodidae). Bulletin of Entomological Research, 89, 303-327 (1999).

22 Pourrut, X., et al. Contribution to the knowledge of ticks (Acarina: Ixodidae) in Gabon. Acarologia, 51(4), 465-471 (2011).

23 Ntiamoa-Baidu, Y. et al. An updated list of the ticks of Ghana and an assessment of the distribution of the ticks of Ghanaian wild mammals in different vegetation zones. Bulletin of Entomological Research, 94, 245-260 (2004).

24 Ntiamoa-Baidu, Y., et al. Ticks associated with wild mammals in Ghana. Bulletin of Entomological Research,95, 205-219 (2005).

25 Morel, P.C. and Mouchet, J. Les Tiques du Cameroun (Ixodidae et Argasidae). Annales de Parasitologie, 40(4), 477-496 (1965).

26 Morel, P.C. Sur quelques larves d'*Ixodes* Latreille, 1746, d'Afrique (Acariens : Ixodidae). Acarologia, VIII, (2), 14pp (1966).

27 Keirans, J.E, Clifford, C.M and Walker J.B. The *Ixodes* (*Afrixodes*) *oldi* group (Acari:Ixodidae) from Sub-Saharan Africa with descriptions of five new species. Journal of Medical Entomology, 19(3), 309-329 (1982).

28 Guglielmone, A.A., Petney, T.N. and Robbins, R.G. Ixodidae (Acari: Ixodoidea): descriptions and redescriptions of all known species from 1758 to December 31, 2019. Zootaxa, 4871(1), 001-322 (2020).

29 Rechav, Y. Attraction of the tick *Ixodes neitzi* to twigs marked by the klipspringer antelope. Nature, 275, 310-311 (1978).

30 Horak, I.G et al. Distribution of endemic and introduced tick species in Free State Province, South Africa. Journal of South African Veterinary Associations, 86(1), 1255 (2015).

31 Clifford, C.M., Walker, J.B. and Keirans, J.E. *Ixodes* (*Afrixodes*) *neitzi* n. sp. (Acarina: Ixodidae) from the mountain reedbuck in South Africa. Onderstepoort Journal of Veterinary Research Institute, 44 (3), 143-150 (1977).

32 Arthur, D.R. The *Ixodes schillingsi* group: ticks of Africa and Madagascar, parasitic on primates, with descriptions of two new species (Ixodoidea, Ixodidae). Parasitology, 544-560 (1956).

33 Horak, I.G, Fourie, L.J. and Braack, L.E.O. Small mammals as hosts of immature ixodid ticks. Onderstepoort Journal of Veterinary Research, 72, 255-261 (2005).

34 Guglielmone, A.A., Nava, S. and Robbins, R.G. Geographic distribution of the hard ticks (Acari: Ixodida: Ixodidae) of the world by countries and territories. Zootaxa, 5251(1): 001-247. (2023).

35 Arthur, D.R. New species of *Ixodes* ticks from Eastern Africa, with a description of the male and nymph of *Ixodes oldi* Nuttall, 1913. Parasitology, 38-69 (1956).

36 Shobana, G. and Gunasekaran, C. A survey of ectoparasites on Mammals in Arignar Anna Zoological Park, Chennai, South India. International Journal of Current Research in Life Science, 7(3), 1285-1288 (2018).

37 Ngoy, S., et al. Using MALDI-TOF mass spectrometry to identify ticks collected on domestic and wild animals from the Democratic Republic of the Congo. Experimental and Applied Acarology, 84 (3), 637-657 (2021).

38 Apanaskevich, D., et al. A new species of *Ixodes* (Acari: Ixodidae) from South African mammals. Journal of Parasitology, 97 (3), 389-398 (2011).

39 Nuttall, G.H.F. Notes on ticks. IV. Relating to the genus *Ixodes* and including a description of three new species and two new varieties. Parasitology, 8(3), 294-337 (1916).

40 Horak,I.G., Braack, L.E.O., Fourie, L.J. and Walker, J.B. Parasites of domestic and wild animals in South Africa. XXXVIII. Ixodid ticks collected from 23 wild carnivore species. Onderstepoort Journal of Veterinary Research, 67, 239-250 (2000).

41 Viljoen, S., et al. Molecular detection of tick-borne pathogens in caracals (*Caracal caracal*) living in human-modified landscapes of South Africa. Parasites & Vectors, 13:220 (2020).
42 Bhat, H.R. and Sreenivasan, M.A. Further records of the ticks of some Reptilian and Mammalian hosts in the Kyasanur Forest Disease area, Karnataka, India. Indian Journal of Parasitology, 5: 207-210 (1981).
43 Muraleedharan, K. Wildlife Arthropods of Karnataka with Special Reference to KFD Endemic Area of Shivamogga District: Part 2 - Those Parasitic on Smaller Mammals, Reptiles and Birds. Veterinary Research International, 5(2), 39-49 (2017).
44 Bedford, G.A.H. A synoptic check-list and host-list of the ectoparasites found on south african Mammalia, Aves, and Reptilia. Onderstepoort Journal of Veterinary Science and Animal Industry, 7(1), 422pp (1936).
45 Baauw, A.H., et al. First records of *Hyalomma rufipes* and *Ixodes neitzi* (Acari: Ixodidae) found on large carnivores in South Africa. Ticks and Tick-Borne Diseases, 10, 128-131 (2019).
46 Kohls, G.M. *Ixodes radfordi*, a new species of tick from rats in Eastern India (Acarina: Ixodidae). The journal of Parasitology, 33(6), 497-498 (1947).
47 Yamathramullage, S. et al. Record of five new endemic small mammal hosts for four ectoparasite species from Sri Lanka. Journal of Asia-Pacific Entomology, 17, 473-476 (2014).
48 Ntiamoa-Baidu, Y. et al. An updated list of the ticks of Ghana and an assessment of the distribution of the ticks of Ghanaian wild mammals in different vegetation zones. Bulletin of Entomological Research, 94, 245-260 (2004).
49 Apanaskevich, D.D, et al. Identity of *Ixodes ugandanus* Neumann, 1906 (Acari:Ixodidae), reinstatement of *I. ampullaceus* Warburton, 1933 as a valid species and redescription of *I. aulacodi* Arthur, 1956, parasites of rodents and other animals in sub-Saharan Africa. Systematic Parasitology, 102: 49 (2025).
50 Hoogstraal H. African Ixodoidea. Volume 1. Ticks of the Sudan. 1-1105 (1956).
51 Morel, P.C. Redescription de *Ixodes djaronensis* Neumann, 1907 (Acariens, Ixodoidea). Acarologia, 7(2), 274-280 (1965).
52 Colbo, M.H. and MacLeod, J. Ecological studies of ixodid ticks (Acari, Ixodidae) in Zambia. II. Ticks found on small mammals and birds. Bulletin of Entomology Research, 66, 489-500 (1976).
53 Colborne, J., Norval, R.A.I. and Spickett, A.M. Ecological studies on *Ixodes* (*Afrixodes*) *matopi* Spickett, Keirans, Norval & Clifford, 1980 (Acarina: Ixodidae). Onderstepoort, Journal of Veterinary Research, 48, 31-35 (1981).
54 Hoogstraal, H. and Theiler, G. Ticks (Ixodoidea, Ixodidae) parasitizing lower primates in Africa, Zanzibar, and Madagascar. The journal of Parasitology, 45(2), 217-222 (1959).
55 Blanco, M.B., et al. Genetic diversity of Ixodid ticks parasitizing Eastern mouse and dwarf lemurs in Madagascar, with descriptions of the larva, nymph, and male of *Ixodes lemuris* (Acari: Ixodidae). Journal of Parasitology, 99(1), 11-18 (2013).
56 Lacroux, C., et al. Survey of ticks and tick-borne pathogens in wild chimpanzee habitat in Western Uganda. Parasites & Vectors, 16-22 (2023).
57 Rajagopalan, P.K., Patil, A.P. and Boshell, M.J. Ixodid ticks on their mammalian hosts in the Kyasanur Forest Disease area of Mysore State, India, 1961-1964. Indian Journal of Medical Research, 56 (Supplement), 510- 526 (1968).
58 Oguge, N.O., et al. Ectoparasites (sucking lice, fleas and ticks) of small mammals in southeastern Kenya. Medical and Veterinary Entomology, 23, 387-392 (2009).
59 Theiler G. The Ixodoidea parasites of vertebrates in Africa south of the Sahara (Ethiopian region). VIII, 280pp (1962).
60 Arthur, D.R and Zulu, F. *Ixodes calcarhebes* n.sp from Zambia. Systematic Parasitology, 1(3/4), 241-244 (1980).
61 Halajian A., et al. Hard ticks (Acari: Ixodidae) parasitizing bushbabies (Mammalia: Galagidae) in a biodiversity hotspot of northern South Africa. Ticks and Tick Borne Diseases, 15(2), 102313 (2024).

62 Matthee, S., et al. Ectoparasite diversity on rodents at De Hoop Nature Reserve, Western Cape Province. African zoology, 45(2), 213-224 (2010).

63 Petney, T.N., et al. Striped mice, *Rhabdomys pumilio*, and other murid rodents as hosts for immature ixodid ticks in the Eastern Cape Province. Onderstepoort journal of Veterinary Research, 71, 313-318 (2004).

64 Matthee, S., et al. Epifaunistic arthropod parasites of the four-striped mouse, *Rhabdomys pumilio*, in the Western Cape Province, South Africa. The journal of Parasitology, 93(1), 47-59 (2007).

65 Yamathramullage, S., et al. *Rickettsiae* reservoirs among small mammals (rats, mice and shrews) and their arthropod vectors in Sri Lanka. Ceylon Journal of Science, 47(2), 175-183 (2018).

66 Kohls, G.M. *Ixodes radfordi*, a new species of tick from rats in Eastern India (Acarina: Ixodidae). The Journal of Parasitology, 33(6), 497-498 (1947).

67 Uilenberg, G. and Hoogstraal, H. *Ixodes nesomys* sp. n. (Ixodoidea, Ixodidae) parasite d'un Rongeur malgache. Annales de Parasitologie, 44, 97-100 (1969).

68 Chitimia-Dobler, L., et al. Description of the male, redescription of the female and 16S rDNA sequence of *Ixodes aulacodi* Arthur, 1956 (Ixodidae). Ticks and Tick-Borne Diseases, 7, 433-438 (2016).

69 Yessinou, R.E., et al. Geographical distribution of hard ticks (Acari: Ixodidae) and tick-host associations in Benin, Burkina-Faso, Ivory-Coast and Togo. Acta Tropica, 232,106510 (2022).

70 Hasle, G., et al. Ticks collected from birds in the northern provinces of South Africa, 2004–2006. Onderstepoort Journal of Veterinary Research, 76, 167-175 (2009).

71 Hoogstraal, H. and Wassef, H. Y. Hosts and distribution of the bird-parasitizing tick *Ixodes (Ixodes) euplecti* in Africa. The Journal of Parasitology, 69(6), 1179-1181 (1983).

72 Arthur, D.R. *Ixodes heinrichi*, sp. nov. from Angola. Parasitology, 52, 207-209 (1962).

73 Hornok, S., et al. On the way between Africa and Europe: Molecular taxonomy of ticks collected from birds in Malta. Ticks and Tick-Borne Diseases, 13, 102001 (2022).

74 Clifford, C.M, Kohls, G.M. and Hoogstraal, H. *Ixodes walkerae*, n. sp., from a bird in Kenya (Agarina: Ixodidae). Journal of Medical Entomology, 5(4), 513-514 (1968).

75 Arthur, D. R. Re-Description of *Ixodes lunatus* Neumann 1907 and of a new species confused therewith, from Madagascar. The Journal of Parasitology, 43(4), 474–483 (1957).

76 Apanaskevich, D.A., Drew, C.E. and Pienaar, R. Description of a new species of *Ixodes* Latreille, 1795 (Acari: Ixodidae) and notes on *I. spinae* Arthur, 1958, parasites of the rock hyrax, *Procavia capensis* (Pallas) (Hyracoidea: Procaviidae) in South Africa. Systematic Parasitology 102, 53 (2025).
